# New Theonellapeptolides from Indonesian Marine Sponge *Theonella swinhoei* as Anti-Austerity Agents

**DOI:** 10.3390/md20110661

**Published:** 2022-10-25

**Authors:** Jabal Rahmat Haedar, Agustinus Robert Uria, Subehan Lallo, Dya Fita Dibwe, Toshiyuki Wakimoto

**Affiliations:** 1 Faculty of Pharmaceutical Sciences, Hokkaido University, Kita 12, Nishi 6, Sapporo 060-0812, Japan; 2 Global Station for Biosurfaces and Drug Discovery, Global Institution for Collaborative Research and Education (GI-CoRE), Hokkaido University, Kita 12, Nishi 6, Sapporo 060-0812, Japan; 3 Faculty of Pharmacy, Hasanuddin University, JI. Perintis Kemerdekaan Km. 10, Makassar 90245, Indonesia; 4 Faculty of Health Sciences, Hokkaido University, Kita-12, Nishi-5, Kita-Ku, Sapporo 060-0812, Japan; 5 Faculty of Sciences, Department of Chemistry, University of Kinshasa, Kinshasa P.O. Box 190 KIN XI, Congo; 6 Department of Biotechnology and Natural Products, Research Center for Applied Sciences and Technologies, 106 Boulevard 30 Juin, Kinshasa P.O. Box 8401 Kinshasa-1, Congo

**Keywords:** marine sponge, *Theonella swinhoei*, depsipeptide, preferential cytotoxicity, theonellapeptolide-type compounds, anti-pancreatic cancer agents, anti-austerity agents

## Abstract

We reported three new members of the theonellapeptolide family from theonellapeptolide II series, namely theonellapeptolides IIb (**1**), IIa (**2**), IIc (**3**), and three known members—IId (**4**), IIe (**5**), and Id (**6**)—from Kodingarengan marine sponge *Theonella swinhoei* collected in Makassar, Indonesia. The structures of tridecadepsipeptides **1**–**3**, including the absolute configurations of their amino acids, were determined by the integrated NMR and tandem MS analyses followed by Marfey’s analysis. To the best of our knowledge, **1** and **2** are the first theonellapeptolide-type compounds to have a valine residue with _D_ configuration at residue position 6. The isolated theonellapeptolide-type compounds **1**–**6** showed selective cytotoxic activity against human pancreatic MIA PaCa-2 cancer cells in a nutrient-deprived medium. Among them, the most potent preferential cytotoxicity was observed in new theonellapeptolide IIc (**3**) and known IId (**4**), IIe (**5**), and Id (**6**).

## 1. Introduction

Marine sponges are a rich source of a diverse array of complex natural products with potent biological activities and unique properties [1,2]. Among sponges, members of the order Tetractinellida (previously called Lithistida) [3], including *Theonella* sp., are known to contain structurally diverse substances, such as polyketides, peptides, alkaloids, and sterols [4,5]. A number of cytotoxic cyclic depsipeptides with unusual amino acid residues have been isolated from the lithistid sponge *Theonella swinhoei* collected worldwide. For example, theopapuamide A containing a high degree of _D_-amino acid residues has been demonstrated to significantly inhibit the growth of the CEM-TART and HCT-116 cell lines [6]. In addition, koshikamide B, a 17-membered peptide macrocycle with 7 *N*-methylated amino acids, showed an inhibitory activity against the P388 and HCT-116 cell lines [7]. A different example is swinhopeptolide A with a 3,4-dimethylglutamine residue, which targeted the Ras-Raf-MEK-ERK signaling pathway [8]. Another interesting class of cyclic depsipeptides reported in *Theonella* sp. is theonellapeptolides, a group of tridecapeptide lactones characterized by the presence of many *N*-methyl amino acids, _D_-amino acids, and *β*-amino acids [9,10,11,12,13,14]. Theonellapeptolide Id, for instance, is composed of three *β*-Ala, three _D_-Leu (one *N*-methylated), two _L_-Val (one *N*-methylated), one *N*-Me-_L_-Ala, one _L_-Thr, one *N*-Me-_L_-Ile, two _D_-*allo*-Ile (one *N*-methylated), and a methoxy acetyl moiety at the N-terminus. Its C terminus is connected to the hydroxyl group of threonine via an ester bond thereby forming a depsipeptide [12,15]. Peptides structurally related to theonellapeptolides include barangamides A–D reported from Indonesian *Theonella* sp. [13]. Biological activities associated with this theonellapeptolide family include moderate cytotoxic activity against the cell line L1210 [15], ion-transport activity for Na^+^, K^+^, and Ca^2+^ ions [16], and immunosuppressive activity [10]. However, there has not been even a single study on theonellapeptolide-type metabolites as a potential source of anti-pancreatic cancer agents. So far, medicinal plants and synthetic approaches furnish most discoveries on anti-pancreatic cancer agents using the anti-austerity strategy [17,18,19,20,21,22,23]. Only a few reports on marine natural products targeting nutrient deprivation in cancer cells were reported to date [24,25].

**Figure 1:**
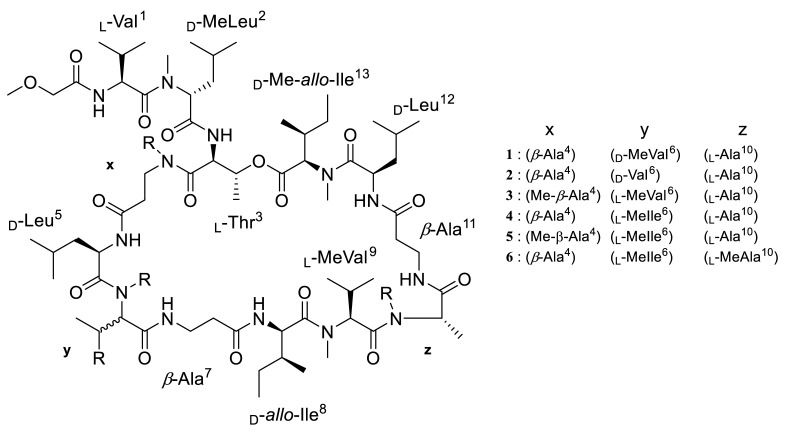
The structure of theonellapeptolides IIb (**1**), IIa (**2**), and IIc (**3**), and known compounds IId (**4**), IIe (**5**), and Id (**6**).

In our search of sponge-derived biologically active compounds, we have recently isolated three new theonellapeptolides IIb (**1**), IIa (**2**) and IIc (**3**) along with two known members of theonellapeptolide II series, IId (**4**) [10] and IIe (**5**) [13], as well as a major theonellapeptolide Id (**6,** I series) [12,26] from the organic extract of *T. swinhoei* collected in Makassar, Indonesia. Based on a combination of NMR and tandem MS analyses followed by Marfey’s analysis, we showed the presence of _D_-Val, _D_-MeVal, or a combination of Me-*β*-Ala and _L_-MeVal in the three new theonellapeptolide-type compounds. We describe here the isolation and structure elucidation of **1**, **2**, and **3** as new theonellapeptolide-type compounds along with two previously reported theonellapeptolides **4** [10] and **5** [13] (Figure 1). All these theonellapeptolide-type I and II series of compounds were found to exhibit cytotoxic activity against pancreatic cancer cells in a nutrient-deprived media condition. The present study was aimed at identifying, characterizing the theonellapeptolides type II series of metabolites from Indonesia sponges and determining their potential effects on pancreatic cancer and antibacterial activities.

## 2. Results and Discussion

### 2.1. Structure Elucidation of Theonellapeptolides **1**–**3**

Three new members of theonellapeptolide II series (**1**–**3**) and known theonellapeptolides IId (**4**) and IIe (**5**) were successfully isolated in this study through a series of separation and purification techniques. The ^1^H-NMR spectra of **1**–**3** in CDCl_3_ showed several broad *N*-methyl protons (*δ*_H_ 2.80 to 3.20), α- protons (*δ*_H_ 3.50 to 5.50), and amide protons (*δ*_H_ 6.90 to 7.80), suggesting the peptidic nature of **1**–**3** (Supporting Figures S20, S26, and S32). However, the broad signals in some key regions in ^1^H-NMR spectra were not improved using other deuterated solvents and/or different temperatures, suggesting the difficulty in determining the planar structures of individual analogues solely based on the NMR spectra. In particular, the ^1^H-^1^H COSY correlations in CDCl_3_ indicated the presence of several rotamers within the purified compounds, clearly exemplified by the signals for threonine and alanine residues (Supporting Figure S2).

**Figure 2:**
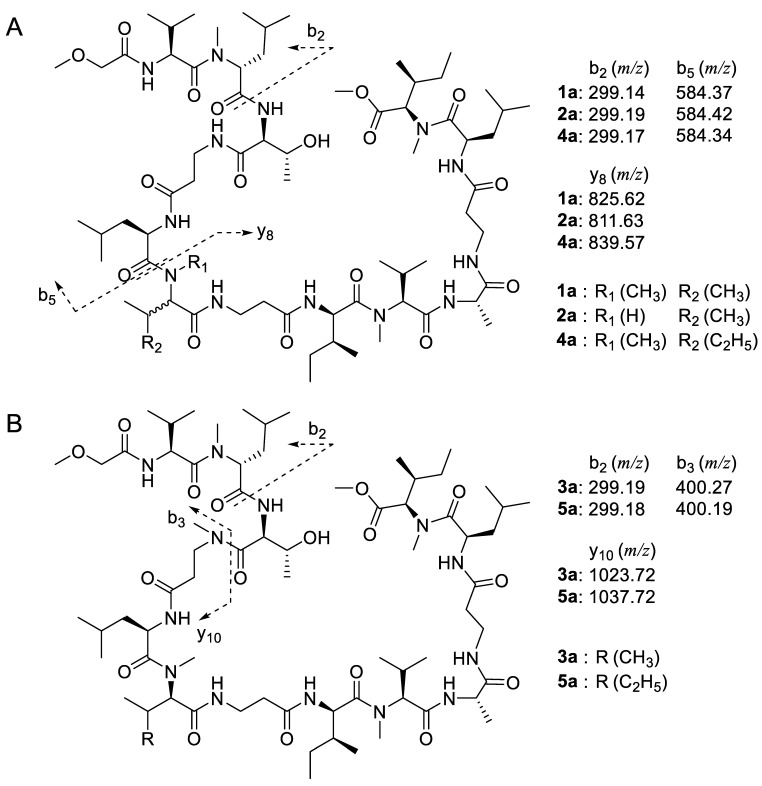
Tandem mass analyses of the *seco*-acid methyl ester peptide of **1**, **2**, and **4** (**A**); **3** and **5** (**B**) showed their three predominant fragment ions.

To overcome the difficulty posed by these broad and overlapped NMR signals, we performed tandem mass (MS2) and MS/MS/MS (MS3) analyses of fragment ions of *seco*-acid methyl esters (**1a**–**5a**) (Figure 2). The *seco*-acid methyl esters (**1a**–**5a**) were generated by the treatment of the cyclic depsipeptides (**1**–**5**) with 7 M ammonia in methanol solution to facilitate ring-opening methyl esterification of the macrolactones. Fragment ions generated from these peptides can be referred to as the b and y series for the N-terminal and the C-terminal fragments, respectively [27,28]. The fragmentation profiles of these linear peptides (**1a**–**3a**) were analyzed in comparison with the *seco*-acid methyl esters (**4a** and **5a**) derived from known compounds (**4** and **5**). The amino acid sequence could be determined, except for amino acid residues with the same molecular weights, such as Ile and Leu. The amino acid residues unidentified by MS were assigned based on NMR data and thus the sequences of new compounds **1**–**3** were determined.

Based on HRESIMS data, the molecular formula of compound **1** is C_68_H_121_O_16_N_13_ (found: *m*/*z* 1398.89294 [M + Na]^+^, calcd. for C_68_H_121_N_13_O_16_Na, 1398.89519), corresponding to the loss of one methylene group of **4** (C_69_H_123_O_16_N_13_) [10]. Tandem MS/MS analysis of the *seco*-acid methyl ester of **1a** showed three predominant fragment ion peaks in the spectrum (Figure 2 and Supporting Figure S3). Two high-intensity fragment ions at *m*/*z* 299.14 and *m*/*z* 584.37 designated as b_2_ and b_5_ ions, respectively, which were also detected in the fragmentation of ring-opened peptide (**4a**) of theonellapeptolide IId (**4**) (Supporting Figure S3), suggesting that the predominant b_2_ and b_5_ fragment ions were corresponding to the N-terminal moieties, *N*-methoxyacetyldipeptide and *N*-methoxyacetylpentapeptide, respectively. The third high-intensity fragment ion peaks were different between **1a**, and **4a**, and appeared at *m*/*z* 825.62, and *m*/*z* 839.57, which were assignable to the remaining C-terminus sequences of **1a** and **4a**, respectively and the 14 mass unit difference suggested the loss of methyl or methylene group at these units (Figure 2, Supporting Figures S3, S4, and S14). We then implemented the structure confirmation of the individual fragment ions (b_2_, b_5_ and y_8_) based on the MS3 fragmentation profiles in conjunction with the NMR data of the intact compound.

**Figure 3:**
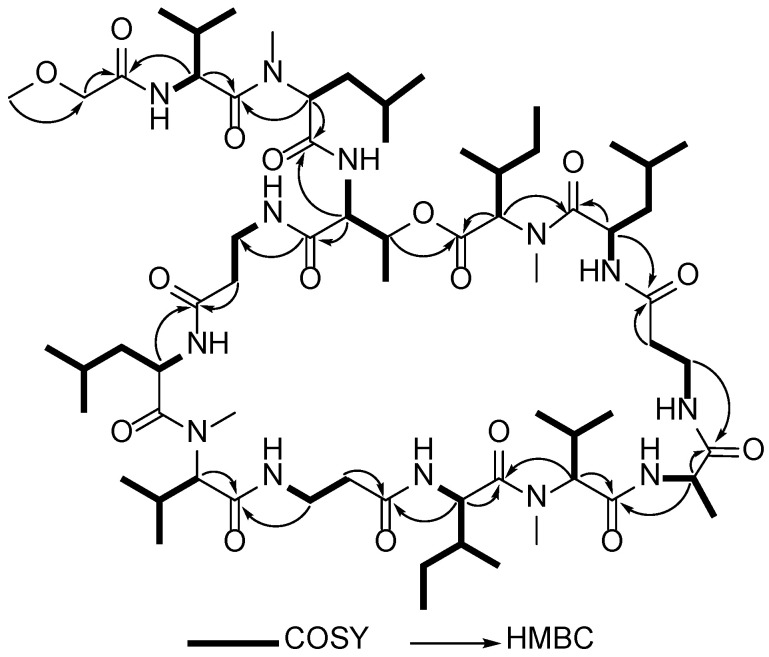
Analysis of ^1^H-^1^H and ^1^H-^13^C key correlations for compound **1**.

The analyses to confirm the amino acid sequence of the b_2_ ion were carefully conducted based on the complicated NMR signals, as listed in Table 1 (Figure 3). The presence of an ester linkage between the C-terminus of the peptide to the hydroxyl group of Thr^3^ was confirmed by HMBC correlation showing the cross-peak between the *β*-CH of Thr^3^ and the carbonyl carbon of a *N*-MeIle^13^. The HMBC spectrum of **1** showed correlation of the Hα of Val^1^ (*δ*_H_ 4.72) to the amide carbonyl position of the methoxy acetyl (MeO-Ac) unit (*δ*_C_ 170.1) (Figure 3 and Table 1), indicating that the N-terminus was capped by MeO-Ac moiety. The presence of a methoxy group on the b_2_ fragment ion of **1a** was supported by its typical ^1^H and ^13^C chemical shifts (*δ*_H_ 3.59 and *δ*_C_ 70.2). Furthermore, the Hα of *N*-MeLeu^2^ (*δ*_H_ 5.08) was connected to the amide carbonyl at *δ*_C_ 173.1 assigned to Val^1^ in **1**. This was supported by the MS3 profile of the b_2_ ion showing the presence of an ion peak at *m*/*z* 172.07 corresponding with the loss of 127 mass units due to the removal of *N*-MeLeu^2^ residue (Supporting Figure S7). The combined tandem MS and HMBC data established the amino acid sequence of the b_2_ ion (*m*/*z* 299.14) as MeO-Ac-Val^1^-*N*-MeLeu^2^.

The MS3 profile of the b_5_ fragment ion (Supporting Figure S6) indicated the presence of smaller fragment ions at *m*/*z* 471.26 (corresponding to the loss of Leu^5^), at *m*/*z* 400.24 (due to the additional removal of *β*-Ala^4^), and at *m*/*z* 299.20 (due to the additional loss of Thr^3^). This was supported by the 2D NMR spectra of **1** showing correlations from the Hα of Thr^3^ (*δ*_H_ 4.39) to the carbonyl signal of *N*-MeLeu^2^ at *δ*_C_ 171.2, and additionally the Hα of *β*-Ala^4^ to the carbonyl of Thr^3^ at *δ*_C_ 168.2. The presence of a *β*-Ala at residue position 4 was confirmed by the COSY and HMBC analyses showing ^1^H-^1^H COSY correlation between the NH at *δ*_H_ 7.25 and H*β* of *β*-Ala^4^ (*δ*_H_ 3.47 and *δ*_H_ 3.26) and subsequently ^1^H-^13^C correlation of the Hα of *β*-Ala^4^ to the carbonyl of Thr^3^ (Figure 3). The combined tandem MS data of b_2_ and COSY/HMBC analyses suggests that the b_5_ fragment ion at *m*/*z* 584.37 covers the N-terminal sequence, MeO-Ac-Val^1^-*N*-MeLeu^2^-Thr^3^-*β*-Ala^4^-Leu^5^.

The MS3 profile of the y_8_-ions derived from **1a** (Supporting Figure S5) and **4a** (Supporting Figure S15) showed similar fragments generated toward C-termini (from *β*-Ala^7^ to *N*-MeIle^13^) and different by 14 mass unit toward N-termini, implying that the variation among these derivatives occurred at amino acid residue position 6. The fragmentation pattern of the y_8_ fragment ion of **1a** in conjunction with its COSY and HMBC data (Figure 3) established the extended amino acid sequence to C-terminus as *N*-MeVal^6^-*β*-Ala^7^-Ile^8^-*N*-MeVal^9^-Ala^10^-*β*-Ala^11^-Leu^12^-*N*-MeIle^13^.

The molecular formula of compound **2** was assigned as C_67_H_119_O_16_N_13_ according to the HRESIMS data (found: *m*/*z* 1384.88153 [M + Na]^+^, calcd. for C_67_H_119_N_13_O_16_Na, 1384.87954) suggesting less one methylene group compared to that of **1**. The ^1^H and ^13^C NMR spectra of **2** showed high similarity to those of **1** with several overlapped signals of methylated nitrogen (*δ*_H_ 2.70 to 3.30 ppm) and amide protons (*δ*_H_ 7.00 to 7.60 ppm). Unfortunately, we were unable to assign several key correlations in its 2D spectra due to the broadening signals and scarce amount. Tandem mass of a methanolysate of **2**, named **2a**, showed the presence of three predominant fragment ions including b_2_ (*m*/*z* 299.19), b_5_ (*m*/*z* 584.42), and y_8_ (*m*/*z* 811.63), which are mimicking the fragmentation pattern profile to those of **1a** and **4a** (Supporting Figure S3). Interestingly, the fragment ion y_8_ of **2a** (*m*/*z* 811.63) showed difference by 14 mass units to y_8_ of **1a** (*m*/*z* 825.59) indicating that the variation of amino acid residue is located at this fragment ion.

Fragment ion b_5_ of **2a** is calculated to cover *N*-methoxyacetylheptapeptide as suggested by MS3 analyses. Fragmentation of b_5_ generated several smaller ions at *m*/*z* 471.25, 400.24, 299.20 indicating loss of Leu^5^, *β*-Ala^4^, and Thr^3^, respectively (Supporting Figure S10). In addition, fragment ion b_2_ (*m*/*z* 299.19) is proposed to cover MeO-Ac-Val^1^ and Me-Leu^2^ moiety similar to **1a** and **4a**. The presence of MeO-Ac in b_2_ is supported by the HMBC analysis of **2** that showed correlations of a methoxy proton at *δ*_H_ 3.59 to a methylene carbon at *δ*_C_ 71.5 that adjacent to a carbonyl amide at *δ*_C_ 170.1. Moreover, correlation of an Hα (*δ*_H_ 4.72) of Val^1^ to the previous carbonyl amide suggesting that it is located next to MeO-Ac moiety. Based on these data, we proposed the amino acid sequence of **2a** as MeO-Ac-Val^1^-MeLeu^2^-Thr^3^-*β*-Ala^4^-and Leu^5^. Furthermore, the MS3 spectrum of y_8_ (Supporting Figure S9 and Table S4) displayed several fragment ions that implying gradually loss of MeIle^13^ to MeVal^9^. Combining the fragment ions generated from MS2 and MS3 analyses of **2a** in addition to several data from complicated NMR spectra of intact compound, allowing us to determine the amino acid sequence of y_8_ of **2a** as Val^6^-*β*-Ala^7^-Ile^8^-*N*-MeVal^9^-Ala^10^-*β*-Ala^11^-Leu^12^ and *N*-MeIle^13^ (Figure 1 and Supporting Figure S9). Thus, compound **2** was determined to have Val at position 6, while **1** contain *N*-MeVal at the same position.

The HRESIMS data showed that compound **3** (C_69_H_123_O_16_N_13_) (found: *m*/*z* 1412.91000 [M + Na]^+^, calcd. for C_69_H_123_N_13_O_16_Na, 1412.91084) lost one methylene (CH_2_) group from **5** (C_70_H_125_O_16_N_13_) [13]. Further tandem MS analysis of the *seco*-acid methyl ester of **3**, designated as **3a**, showed three high-intensity fragment ions, namely b_2_ at *m*/*z* 299.19, b_4_ at *m*/*z* 400.27, and y_10_ at *m*/*z* 1023.72 (Supporting Figure S11 and Table S5). The fragmentation profile of **3a** was apparently similar with that of the ring-opened derivative of known theonellapeptolide IIe (**5a**); b_2_ *m*/*z* 299.18, b_4_ *m*/*z* 400.19, and b_10_ *m*/*z* 1037.72 (Figure 2B, Supporting Figure S17, and Table S10). The b_4_ fragment ions of **3a** and **5a** at *m*/*z* 400.10 were assigned as MeO-Ac-Val^1^-*N*-MeLeu^2^-Thr^3^ in order from N-terminus (Figures S13 and S19). The y_10_ fragment ion peak at *m*/*z* 1023.72 was subsequently subjected to tandem MS/MS/MS analysis (Figure S12; Table S6, Supplementary), allowing us to assign it as *N*-Me-*β*-Ala^4^-Leu^5^-*N*-MeVal^6^-*β*-Ala^7^-Ile^8^-*N*-MeVal^9^-Ala^10^-*β*-Ala^11^-Leu^12^-*N*-MeIle^13^ in order from the N-terminus. The y_10_ fragment ion of **3a** (*m*/*z* 1023.72) differs from that of **5a** (*m*/*z* 1037.72) only in the substitution of *N*-MeVal^6^ for *N*-MeIle^6^ (Figure 1).

**Figure 4:**
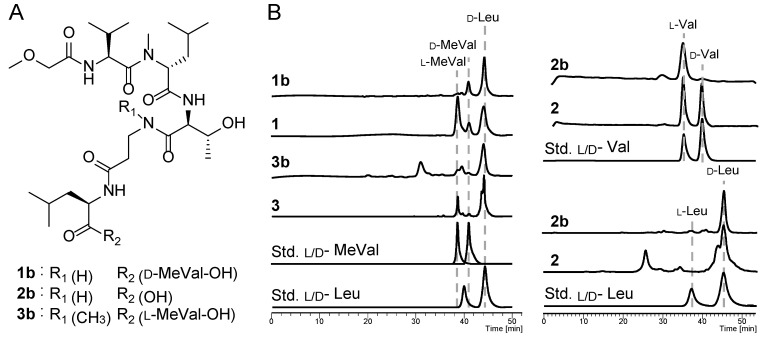
Isolated peptide fragment for determining the amino acid configuration of repeated residues (**A**). Determination of absolute configuration of repeated amino acids (**B**).

Upon determining the amino acid sequences of the isolated compounds, we deduced the configurations of the amino acid residues by means of Marfey’s analysis [29]. Compounds **1**–**3** consisted of several duplicated amino acids such as methyl valine (duplicated in **1** and **3**), valine (duplicated in **2**), and leucine (duplicated in **1**–**3**) (Figure 1). Hence, to unambiguously determine the configuration of each residue, we performed partial hydrolysis of **1**–**3** to obtain a peptide fragment containing one of each duplicated residue (Figure 4A) followed by Marfey’s analysis. We then compared the results to those of the obtained from the totally hydrolyzed of intact compounds.

Targeted fragment peptide of **1** corresponding to N-terminus, designated as fragment methoxy acetyl hexapeptide **1b** as confirmed by MS/MS analysis (Supporting Figure S38A). Subsequent Marfey’s analysis of **1b** (Supporting Figure S38B) enabled us to deduce the absolute configuration of the first five residues at the N-terminus as _L_-Val^1^, _D_-*N*-MeLeu^2^, _L_-Thr^3^, _D_-Leu^5^, and _D_-MeVal^6^, respectively. In addition, Marfey’s analysis of the total hydrolysate of **1** (Supporting Figure S39) exhibited only one residue of _D_-Leu and enantiomeric mixture of _D_ and _L_ -*N*-MeVal. Comparison of data obtained from **1b** and **1** allowed us to determine the absolute configuration of the *N*-MeVal^9^ in **1** as _L_-configuration (Figure 4B).

Using similar strategy, the peptide fragment **2b** was purified from the hydrolysate of **2** after treatment with mild acid condition. Upon confirmation through tandem MS analysis (Supporting Figure S40A), the pentapeptide **2b** and the intact compound **2** were further subjected to Marfey’s analysis (Supporting Figures S40B and S41). As the result, the absolute configuration of the N-terminal four amino acid residues was determined as _L_-Val^1^, _D_-*N*-MeLeu^2^, _L_-Thr^3^, and _D_-Leu^5^. In addition, the absolute configuration of Val^6^ and Leu^12^ was deduced to be _D_-configuration based on the comparison of the obtained Marfey’s analysis data of both compounds, **2b** and **2** (Figure 4B).

Finally, the hexapeptide **3b** was purified from partial hydrolysate of **3** as confirmed by tandem MS analysis (Supporting Figure S42A). Subjecting **3b** to Marfey’s analysis enabled determination of the absolute configuration of its amino acid residues as _L_-Val^1^, _D_-*N*-MeLeu^2^, _L_-Thr^3^, and _D_-Leu^5^ (Supporting Figure S42B). It was difficult to determine the absolute configuration of *N*-MeVal in this step due to a low peak-resolution generated from small amount of sample (Figure 4B). However, Marfey’s analysis of the total hydrolysate of **3** (Supporting Figure S43) showed only one residue of _D_-Leu and _L_-*N*-MeVal, suggesting the _D_-configuration of both Leu residues and the _L_-configuration of both *N*-MeVal residues (Figure 4B).

In summary, based on Marfey’s analysis results described above, we assigned the identity and absolute configuration of the amino acid at residue position 6 as _D_-MeVal in **1**, _D_-Val in **2**, and _L_-MeVal in **3**. These data placed **1** and **2** as the first members of theonellapeptolide family to have a valine residue with _D_ configuration at the position 6. We therefore propose **1** and **2** as new theonellapeptolide analogues, designated here as theonellapeptolides IIb and IIa. Furthermore, the presence of a _L_-*N*-MeVal at residue position 6 and a *N*-Me-*β*-alanine at residue position 4 in **3** places this compound as a new analogue designated here as theonellapeptolide IIc.

### 2.2. Investigation of Biological Activities

#### 2.2.1. Antibacterial Activity

Previously, three theonellapeptolide members capped with a unique acyl group at their N-termini such as methylsulfinylacetyl, methylsulfanylacetyl, and acetyl were isolated by Tsuda et al. from Okinawan marine sponge *Theonella* sp. [14]. Furthermore, the antibacterial activity of these variants was evaluated against four bacterial and six fungal strains. Theonellapeptolide variant with a methylsulfinylacetyl or an acetyl group exhibited growth inhibition against *Trichophyton memtagrophytes*, *Aspergillus niger*, *Micrococcus luteus*, and *Bacillus subtillis.* In contrast, the other variant with methylsulfanylacetyl group showed no antimicrobial activity [14]. To the best of our knowledge, there is no report on the antibacterial activity of theonellapeptolides with methoxyacetyl group. This encouraged us to investigate the antibacterial activity of **1**–**6** against the Gram-negative bacterium *Escherichia coli* JW5503 and the Gram-positive bacteria *Bacillus cereus* NBRC 15305 and *Kocuria rhizophila* NBRC 12708. In this study, all tested theonellapeptolide variants (**1**–**6**) contain methoxyacetyl group blocked at their N-termini with similar amino acid residues. The assay results indicated that none of the tested compounds (**1**–**6**) showed any antibacterial activity even at the highest given concentration (66 μg/mL) (Table S11). These data suggest that the type of acyl group at the N-terminus among theonellapeptolide was crucial for their antibacterial activity.

#### 2.2.2. Anti-austerity Activity against the MIA PaCa-2 Cell Line

**Figure 5:**
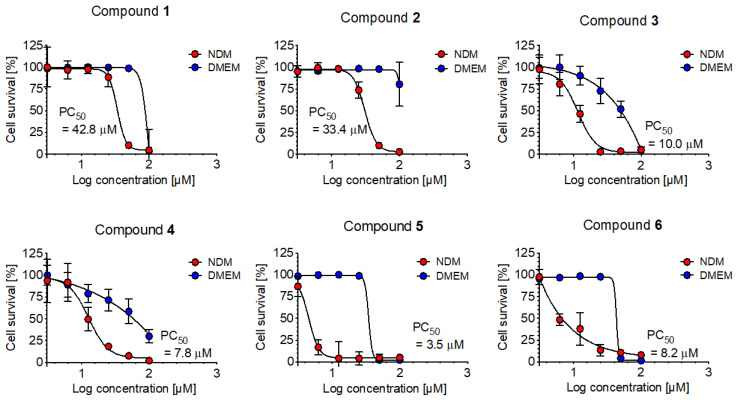
Cytotoxic and anti-austerity activity of the compounds **1**–**6** against the human pancreatic cancer cell line MIA PaCa-2. Compound **1**–**6**: theonellapeptolides IIb (**1**), IIa (**2**), and IIc (**3**), and known compounds IId (**4**), IIe (**5**), and Id (**6**).

Newly isolated compounds **1**–**3**, along with known **4, 5** (theonellapeptolides type II series), and **6** (theonellapeptolides type I) were subjected to cytotoxic activity against pancreatic cancer cells in a nutrient-deprived media condition using anti-austerity strategy [30]. The anti-pancreatic cancer cytotoxic activity of all isolated theonellapeptolide-type compounds (**1**–**6**) were evaluated against the MIA PaCa-2 cell line. Data are presented as preferential cytotoxicity (PC_50_) values, which represent the concentration that kills 50% of cancer cells in a nutrient-deprived medium (NDM) without any toxicity in a nutrient-rich medium DMEM (Dulbecco’s Modified Eagle Medium). Among the tested compounds, theonellapeptolides **3**, **4**, **5** and **6** exhibited the most potent preferential cytotoxicity, with PC_50_ value of 10.0, 7.8, 3.5 and 8.2 μM, respectively (Figure 5). The isolated members of theonellapeptolide II series (**1**–**5**) exhibited preferential cytotoxic activity under NMD condition. Those compounds (**1**–**5**) share similar amino acid residue in their structure. The structure–anti-austerity activity relationship of **1**–**5** revealed that the combination of *N*-Me-*β*-Ala^4^ and *N*-MeIle^6^ gave more potent activity with a PC_50_ value below 4 μM as shown by **5** (3.5 μM). The presence of only one of these residues in their structure showed potent preferential cytotoxicity at the range of 7.8–10 μM as observed in **4** containing a *N*-MeIle^6^ (7.8 μM) and **3** containing *N*-Me-*β*-Ala^4^ (10.0 μM). However, the absence of these two residues displayed a moderate activity at the range of 33.4–42.8 μM. On the other hand, the presence of *N*-MeIle^6^ in theonellapeptolide Id (**6**) showed activity similar to compound **4** and **3** with PC_50_ value of 8.2 μM (Figure 5). These data suggested that the presence of *N*-Me-*β*-Ala^4^ and/or *N*-MeIle^6^ is important feature for the preferential cytotoxic activity of theonellapeptolide-type compounds. Therefore, theonellapeptolides **1–6** and their derivatives represent excellent anti-cancer candidates under nutrient starvation condition.

**Figure 6:**
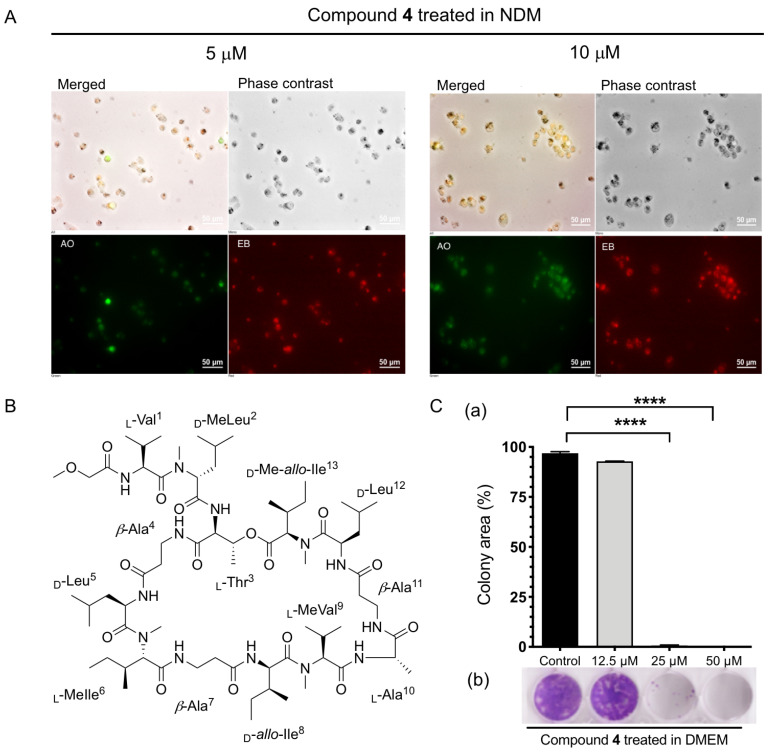
(**A**) Morphological changes of MIA PaCa-2 cells induced by **4** in a nutrient-deprived medium (NDM). MIA PaCa-2 tumor cells were treated with **4** at the indicated concentrations in NDM in a 24-well plate and incubated for 24 h. The cells were stained with ethidium bromide (EB) and acridine orange (AO), and photographed in the fluorescence (red and green) under phase contrast modes using a Nikon sCMOS camera oxford instrument, Hamamatsu Photonics/Ti-E. (**B**) Structure of bioactive compound **4**. (**C**) Effect of **4** on colony formation by MIA PaCa-2 cells in DMEM. (**a**) Graph showing the average values of the area occupied by MIA PaCa-2 cell colonies (three replicates). **** *p* < 0.0001 compared to the untreated control group. (**b**) Representative wells showing colonies of MIA PaCa-2 cells.

**Figure 7:**
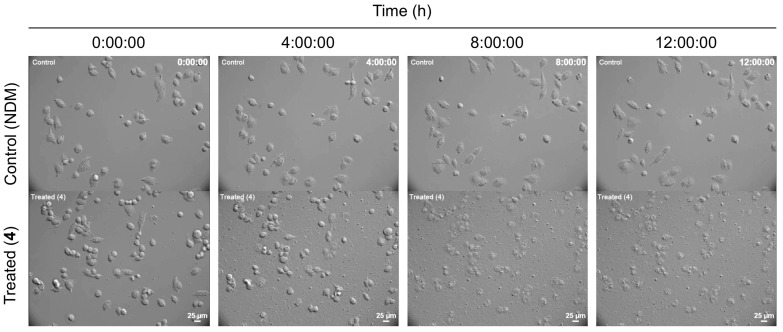
Captures of the live imaging of the effect of 10 μM of compound **4** (treated in NDM, below) compared to control (untreated in NDM, up) on MIA PaCa-2 cells at different intervals of time 4 h.

Among the isolated theonellapeptolide II series, the major compound **4** and minor compounds **3** and **5** were found to be the most active (PC_50_ 7.8, 10.0 and 3.5 μM, respectively). Thus, more detailed studies were performed on the major compound theonellapeptolide IId (**4**). Its effect on morphology status of MIA PaCa-2 cells under starvation condition was studied using a double-staining assay with EB (ethidium bromide) and AO (acridine orange) [17,18,19,20,21]. The membranes of dead or dying cells are penetrated by EB and staining in red while living cells are stained by AO, a cell-membrane-permeable dye emitting bright green fluorescence. Both AO and EB stains are known as DNA-intercalating agents. During cell death, EB and AO enter into the cell after the integrity of the cell membrane is disrupted, which facilitates the emission of orange fluorescence [17,18,19,20,21]. In this study, MIA PaCa-2 cells treated with 5 and 10 μM of theonellapeptolide IId (**4**) were incubated in starvation condition (NDM) for 24 h, and stained with the EB and AO dyes. In Figure 6A, treatment with **4** led to a dose-dependent increase in EB-stained cells emitting red fluorescence and showing drastic alteration of cell morphology. Incubation of MIA PaCa-2 cells with **4** at 5 and 10 μM even resulted in an exclusive red EB. To show the details of cell death in real time, live evidence of the anticancer potential of **4** was studied. A time-lapse live imaging anti-pancreatic cancer experiment under NDM condition was performed for the first time in a multi position in the dish, for monitoring the effect of **4**. Moreover, MIA PaCa-2 cells were treated with 10 μM of compound **4** in NDM and incubated at 37 °C at 5% CO_2_. Real-time anti-austerity potential tracking of **4** showing morphological changes in MIA PaCa-2 cells under NDM was monitored using a Nikon sCMOS camera oxford instrument, Hamamatsu Photonics/Ti-E, for 24 h. Images were captured every 10 min by rotating at several fixed positions in the dish under the phase contrast mode on a CCD Camera imaging system for 24 h. Treatment with **4** under NDM inhibited cell mobility immediately within 60 min and altered morphology of MIA PaCa-2 cells after 4 h was observed, then cell death started after 8 h, finally in 12 h complete cell death was observed (Figure 7, Movies S1 and S2, Supporting Information). In the control, MIA PaCa-2 cells survived 24 h until the end of the experiment in the NDM (Figure 7). Thus, the result of this study provided the first simultaneous multi-site live evidence of the anti-pancreatic cancer therapeutic potential of the compound theonellapeptolide IId (**4**) against MIA PaCa-2 cells as marine-based anti-austerity agents.

Pancreatic cancer cells have a great ability to migrate to surrounding tissues, growing and forming new colonies after metastasis in distant organs with sufficient nutrition. These processes are responsible for the majority of deaths from pancreatic cancer. The phenomenon is called “colonization”, and occurs during cancer metastasis. Migrating pancreatic cancer cells are likely to form small colonies (micrometastases), which then become large tumors [31]. The discovery of potential inhibitors of the colony-forming ability of pancreatic cancer represents a promising approach to prevent pancreatic tumor metastasis. Recently, various natural and synthetic compounds have been found to exhibit pancreatic cancer colony-forming inhibitory activities [19,20,21,22]. Therefore, theonellapeptolides isolated from marine origin were studied for their ability to inhibit MIA PaCa-2 colony formation. MIA PaCa-2 cells (500 cells/well) were placed in a 24-well plate and treated with **4** at 12.5, 25, and 50 μM (non-cytotoxic concentrations) in DMEM for 24 h.

Subsequently, the medium was replaced with fresh DMEM, and the cells were allowed to grow as colonies for 10 days in a humified CO_2_ incubator at 37 °C. Upon exposure to **4** at a non-cytotoxic concentration for 24 h in rich condition (DMEM), however, colony formation was found to be significantly inhibited. The untreated MIA PaCa-2 cells grew rapidly to form many numbers of colonies occupying 100% of the total well area as also observed with the treatment of 12.5 μM of compound **4**. A total inhibition of colony formation was observed at 25 and 50 μM for cells treated with compound **4**. (Figure 6C).

#### 2.2.3. Anti-Austerity Activity against the HepG2 (Human Liver Cancer Cells) and MCF-7 (Human Breast Cancer Cells)

Finally, the anti-austerity activity on two other different cancer cell lines was studied. Preferential cytotoxicity was assessed in a nutrient-deprived medium. Esumi et al. hypothesized that tolerance to nutrient deprivation as well as angiogenesis might be an important factor in tumor progression under hypovascular condition [30]. Cell death occurred within 36 h in liver cancer cell lines, including the HepG2 cell lines. MCF-7 cells (human breast cancer cells) survived for over 48 h under starvation condition in this study while pancreatic cancer cells survived for remarkably longer period up to 72 h [30]. In this study, all isolated theonellapeptolide compounds (**1**–**6**) were evaluated against the HepG2 (hepatoma human liver cancer cells) and MCF-7 (human breast cancer cells) cell lines using the anti-starvation strategy. The results also suggested that no significant preferential cytotoxicity for all the tested compounds on those two cell lines.

## 3. Materials and Methods

### 3.1. Chemicals and Materials

Optical rotations were measured on a Jasco P-1030 polarimeter (JASCO, Tokyo, Japan). Infrared spectra were measured on a Jasco FT/IR 4100 (JASCO, Japan). NMR spectra were recorded on a JEOL ECX 500 (500 MHz) or a Bruker DRX (500 MHz) spectrometer (Bruker, Billerica, MA, USA). Chemical shifts are denoted in *δ* (ppm) relative to residual solvent peaks as internal standard (CDCl_3_, ^1^H *δ* 7.24, ^13^C *δ* 77.0). ESI–MS spectra were recorded on a Thermo Scientific Exactive mass spectrometer (Thermo Fisher Scientific, Waltham, MA, USA) or a SHIMADZU LCMS-2020 spectrometer (Shimadzu, Kyoto, Japan). High performance liquid chromatography (HPLC) experiments were performed with a SHIMADZU HPLC system equipped with a LC-20AD intelligent pump. LC–MS experiments were performed with amaZon SL (Bruker Daltonics, Bremen, Germany). Cell density for cytotoxic and anti-microbial assay was recorded on Tecan infinite^®^ M200 plate reader (Tecan, Salzburg, Austria) at Drug Discovery Scientific Research and Education Center-Open Lab. facility, Faculty of Pharmaceutical Sciences, Hokkaido University. All reagents were used as supplied unless otherwise stated.

### 3.2. Animal Material

Marine Sponge *Theonella swinhoei* with voucher number of AK-I was collected by scuba diving in Kodingareng Keke Island near Makassar, Indonesia in August 2015. The specimen was immediately frozen after collection and kept until processed.

### 3.3. Extraction and Isolation

Approximately 1.0 kg (wet weight) of the marine sponge *Theonella swinhoei* collected in Kondingareng Island, South Sulawesi, Indonesia, the same collection site as reported previously [10], was extracted three times with 1 L methanol and concentrated under vacuo. The methanolic extract was separated with normal-phase thin-layer chromatography (TLC) using chloroform and methanol (9:1) as the mobile phase. After TLC analysis with Dragendorff’s reagent, three brown spots appeared at R_f_ values of 0.31, 0.49, and 0.71, respectively (Supporting Figure S1), suggesting the presence of relatively non-polar nitrogen-containing compounds such as alkaloids and/or peptides. Subsequent ESI–MS analyses indicated that the second spot may contain novel analogues. This motivated us to isolate and characterize them. Subsequently, the crude methanolic extract (13.0 g) was partitioned between ethyl acetate (300 mL × 3 times) and water (300 mL) to afford ethyl acetate soluble material (6.0 g). Subsequently, non-polar portion (ethyl acetate soluble material) was subjected to gel filtration chromatography (Sephadex LH-20) with methanol as the mobile phase to give 160 fractions. Furthermore, fractions containing target compound (fr. 62–78) were pooled and concentrated under vacuo. The residue (2.70 g) was further separated by silica gel open column chromatography [Silica gel 60N (spherical, neutral, 40–50 µm), Kanto Chemical Co., Inc.] using the step gradient of mobile-phase chloroform: methanol; [0%, 2.5%,5%, 7.25%, 10% (% methanol)], and washed with chloroform: methanol: water (4:5:1) to afford 112 fractions. It was found that the fractions eluted with 5% methanol contained compounds with new molecular weights based on ESI–MS analyses in comparison with those available in MarinLit—a database of the marine natural products literature (http://pubs.rsc.org/marinlit/, accessed on the 3 October 2016. Finally, fractions containing target compounds were purified through high pressure liquid chromatography equipped with Cosmosil^®^ 5C^18^-MS-II (10 mm ID × 250 mm) using 75% acetonitrile in water with additional 0.05% TFA to afford three new analogues **1** (9.0 mg), **2** (4.0 mg), and **3** (3.0 mg) (Figure 1 and Supporting Figure S1). In addition, two known theonellapeptolides IId (**4**, 40.0 mg) and IIe (**5**, 8.0 mg) were identified based on the comparison of their spectroscopic data with those previously reported by Kobayashi et al. (1994) [10] and Roy et al. (2000) [13]. Finally, a major theonellapeptolide Id (**6**) was also purified from different fractions and its spectra was compared to the previously reported [12,26].

Theonellapeptolide IIb (**1**): Colorless solid; [α]^25^_D_ −22.9622 (c 0.31, MeOH); ^1^H and ^13^C NMR data are shown in Table 1; HRESIMS *m*/*z* 1398.89294 [M + Na]^+^ (calcd. for C_68_H_121_N_13_O_16_Na, 1398.89519). IR: ν_max_ 3300, 2960, 1630, 1520 cm^−1^.

Theonellapeptolide IIa (**2**): Colorless solid; [α]^22^_D_ −35.500 (c 0.7, MeOH); ^1^H NMR (CDCl_3_) *δ* [MeOAc] 3.59 (3H, m, CH_3_), 3.85 (2H, m, CH_2_), [_L_-Val^1^] 7.18 (1H, m, NH), 4.72 (1H, m, Hα), 2.22 (1H, m, H*β*), 0.78 (3H, m, CH_3_), 0.93 (3H, m, CH_3_), [_D_-MeLeu^2^] 3.04 (3H, m, NCH_3_), 5.10 (1H, m, Hα), 2.01 (1H, m, H*β*), 1.39 (1H, m, H*β*), (Hγ: n.r.), 0.83 (3H, m, CH_3_), 0.90 (3H, m, CH_3_), [_L_-Thr^3^] 7.55 (1H, m, NH), 4.38 (1H, m, Hα), 5.10 (1H, m, H*β*), 1.07 (3H, m, CH_3_), [*β*-ala^4^] 7.20 (1H, m, NH), 2.40 (1H, m, Hα), 2.32 (1H, m, Hα), 3.43 (1H, m, H*β*), 3.37 (1H, m, H*β*), [_D_-Leu^5^] 7.61 (1H, m, NH), 4.93 (1H, m, Hα), 1.48 (1H, m, H*β*), 1.46 (1H, m, H*β*), (Hγ: n.r.), 0.90 (3H, m, CH_3_), 0.88 (3H, m, CH_3_), [_D_-Val^6^] 7.22 (1H, m, NH), 4.71 (1H, m, Hα), 2.00 (1H, m, H*β*), 0.85 (3H, m, CH_3_), 0.91 (3H, m, CH_3_), [*β*-ala^7^] 7.50 (1H, m, NH), 2.38 (1H, m, Hα), 2.42 (1H, m, Hα), 3.59 (1H, m, H*β*), 3.39 (1H, m, H*β*), [_D_-*allo*-Ile^8^] 7.25 (1H, m, NH), 4.75 (1H, m, Hα), 2.15 (1H, m, H*β*), 1.54 (2H, m, Hγ), 0.93 (3H, m, CH_3_), 0.78 (3H, m, CH_3_), [_L_-MeVal^9^] 2.98 (3H, m, NCH_3_), 4.65 (1H, m, Hα), 2.21 (1H, m, H*β*), 0.78 (3H, m, CH_3_), 0.95 (3H, m, CH_3_), [_L_-Ala^10^] 7.55 (1H, m, NH), 4.22 (1H, m, Hα), 1.15 (3H, m, CH_3_), [*β*-ala^11^] 7.55 (1H, m, NH), 2.48 (1H, m, Hα), 2.19 (1H, m, Hα), 3.52 (1H, m, H*β*), 3.26 (1H, m, H*β*), [_D_-Leu^12^] 7.25 (1H, m, NH), 4.88 (1H, m, Hα), 1.28 (1H, m, H*β*), 1.43 (1H, m, H*β*), (Hγ: n.r.), 0.83 (3H, m, CH_3_), 0.85 (3H, m, CH_3_), [_D_-Me-*allo*-Ile^13^] 2.99 (3H, m, NCH_3_), 5.15 (1H, m, Hα), 1.71 (1H, m, H*β*), 1.38 (1H, m, Hγ), 1.16 (1H, m, Hγ), 0.92 (3H, m, CH_3_), 0.82 (3H, m, CH_3_); ^13^C NMR (CDCl_3_) *δ* [MeOAc] 70.2 (CH_3_), 71.5 (CH_2_), 170.1 (CO), [_L_-Val^1^] 62.2 (CH, Cα), 26.8 (CH, C*β*), 18.7 (CH_3_), 19.8 (CH_3_), 173.2 (CO), [_D_-MeLeu^2^] 30.8 (NCH_3_), 55.0 (CH, Cα), 37.0 (CH_2_, C*β*), (Cγ: n.r.), 20.4 (CH_3_), 19.4 (CH_3_), (CO: n.r.), [_L_-Thr^3^] 55.9 (CH, Cα), 70.2 (CH, C*β*), 17.2 (CH_3_), 168.1 (CO), [*β*-ala^4^] 35.8 (CH_2_, Cα), 36.0 (CH_2_, C*β*), 171.4 (CO), [_D_-Leu^5^] 47.9 (CH, Cα), 41.2 (CH_2_, C*β*), (Cγ: n.r.), 20.0 (CH_3_), 21.7 (CH_3_), (CO: n.r.), [_D_-Val^6^] 54.0 (CH, Cα), 31.1 (CH, C*β*), 17.5 (CH_3_), 19.3 (CH_3_), 170.4 (CO), [*β*-ala^7^] 35.8 (CH_2_, Cα), 36.0 (CH_2_, C*β*), 172.5 (CO), [_D_-*allo*-Ile^8^] 53.9 (CH, Cα), (C*β:* n.r.), 25.4 (CH_2_ Cγ), 21.2 (CH_3_), 23.2 (CH_3_), (CO: n.r.), [_L_-MeVal^9^] 30.9 (NCH_3_), 62.6 (CH, Cα), 25.8 (CH, C*β*), 18.7 (CH_3_), 19.8 (CH_3_), 169.4 (CO), [_L_-Ala^10^] 48.7 (CH, Cα), 17.2 (CH_3_), 172.7 (CO), [*β*-ala^11^] 35.8 (CH_2_, Cα), 36.0 (CH_2_, C*β*), 171.5 (CO), [_D_-Leu^12^] 47.8 (CH, Cα), 41.9 (CH_2_, C*β*), (Cγ: n.r.), 21.3 (CH_3_), 20.3 (CH_3_), (CO: n.r.), [_D_-Me-*allo*-Ile^13^] 31.2 (NCH_3_), 54.8 (CH, Cα), (C*β:* n.r.), 24.7 (CH_2_ Cγ), 21.4 (CH_3_), 23.3 (CH_3_), (CO: n.r.); HRESIMS *m*/*z* 1384.88153 [M + Na]^+^ (calcd. for C_67_H_119_N_13_O_16_Na, 1384.87954). IR: ν_max_ 3300, 2960, 1630, 1530 cm^−1^.

Theonellapeptolide IIc (**3**): Colorless solid; [α]^23^_D_ −55.1241 (c 1.45, MeOH); ^1^H NMR (CDCl_3_) *δ* [MeOAc] 3.59 (3H, m, CH_3_), 3.89 (2H, m, CH_2_), [_L_-Val^1^] 7.19 (1H, m, NH), 4.78 (1H, m, Hα), 1.99 (1H, m, H*β*), 0.77 (3H, m, CH_3_), 0.95 (3H, m, CH_3_), [_D_-MeLeu^2^] (NCH_3_: n.r.), 5.10 (1H, m, Hα), 1.52 (1H, m, H*β*), 1.43 (1H, m, H*β*), 1.72 (1H, m, Hγ), 0.81 (3H, m, CH_3_), 0.92 (3H, m, CH_3_), [_L_-Thr^3^] 7.75 (1H, m, NH), 4.84 (1H, m, Hα), 5.17 (1H, m, H*β*), 1.07 (3H, m, CH_3_), [Me-*β*-ala^4^] 2.95 (3H, m, NCH_3_), 2.48 (1H, m, Hα), 2.30 (1H, m, Hα), 3.50 (1H, m, H*β*), 3.20 (1H, m, H*β*), [_D_-Leu^5^] 7.12 (1H, m, NH), 4.85 (1H, m, Hα), 1.35 (1H, m, H*β*), 1.47 (1H, m, H*β*), 1.99 (1H, m, Hγ), 0.93 (3H, m, CH_3_), 0.88 (3H, m, CH_3_), [_L_-MeVal^6^] 3.07 (3H, m, NCH_3_), 4.77 (1H, m, Hα), 2.01 (1H, m, H*β*), 0.88 (3H, m, CH_3_), 0.95 (3H, m, CH_3_), [*β*-ala^7^] 7.19 (1H, m, NH), 2.30 (1H, m, Hα), 2.48 (1H, m, Hα), 3.50 (1H, m, H*β*), 3.20 (1H, m, H*β*), [_D_-*allo*-Ile^8^] 7.89 (1H, m, NH), 4.84 (1H, m, Hα), 2.26 (1H, m, H*β*), 1.22 (1H, m, Hγ), 1.45 (1H, m, Hγ), 0.93 (3H, m, CH_3_), 0.85 (3H, m, CH_3_), [_L_-MeVal^9^] 3.05 (3H, m, NCH_3_), 4.66 (1H, m, Hα), 2.21 (1H, m, H*β*), 0.78 (3H, m, CH_3_), 0.96 (3H, m, CH_3_), [_L_-Ala^10^] 7.31 (1H, m, NH), 4.22 (1H, m, Hα), 1.25 (3H, m, CH_3_), [*β*-ala^11^] 7.20 (1H, m, NH), 2.37 (2H, m, Hα), 3.23 (1H, m, H*β*), 3.57 (1H, m, H*β*), [_D_-Leu^12^] 7.58 (1H, m, NH), 4.92 (1H, m, Hα), 1.22 (1H, m, H*β*), 1.45 (1H, m, H*β*), 1.59 (1H, m, Hγ), 0.86 (3H, m, CH_3_), 0.91 (3H, m, CH_3_), [_D_-Me-*allo*-Ile^13^] 3.08 (3H, m, NCH_3_), 4.57 (1H, m, Hα), 1.71 (1H, m, H*β*), 1.41 (1H, m, Hγ), 1.15 (1H, m, Hγ), 0.92 (3H, m, CH_3_), 0.87 (3H, m, CH_3_); ^13^C NMR (CDCl_3_) *δ* [MeOAc] 70.2 (CH_3_), 71.5 (CH_2_), 170.1 (CO), [_L_-Val^1^] 53.6 (CH, Cα), 26.0 (CH, C*β*), 18.7 (CH_3_), 19.4 (CH_3_), (CO: n.r.), [_D_-MeLeu^2^] 30.8 (NCH_3_), 55.2 (CH, Cα), 37.0 (CH_2_, C*β*), (Cγ: n.r.), 21.1 (CH_3_), 21.5 (CH_3_), 171.2 (CO), [_L_-Thr^3^] 55.1 (CH, Cα), 69.8 (CH, C*β*), 17.0 (CH_3_), (CO: n.r.), [Me-*β*-ala^4^] (NCH_3_: n.r.), 35.8 (CH_2_, Cα), 35.9 (CH_2_, C*β*), 171.3 (CO), [_D_-Leu^5^] (Cα: n.r.), 41.3 (CH_2_, C*β*), (Cγ: n.r.), 20.2 (CH_3_), 21.6 (CH_3_), (CO: n.r.), [_L_-MeVal^6^] (NCH_3_: n.r.), 53.7 (CH, Cα), 31.2 (CH, C*β*), 21.6 (CH_3_), 20.4 (CH_3_), (CO: n.r.), [*β*-ala^7^] 35.8 (CH_2_, Cα), 35.9 (CH_2_, C*β*), (CO: n.r.), [_D_-*allo*-Ile^8^] 60.3 (CH, Cα), (C*β:* n.r.), 24.5 (CH_2_ Cγ), 18.4 (CH_3_), 17.4 (CH_3_), (CO: n.r.), [_L_-MeVal^9^] (NCH_3_: n.r.), 62.4 (CH, Cα), 25.9 (CH, C*β*), 18.8 (CH_3_), 20.3 (CH_3_), (CO: n.r.), [_L_-Ala^10^] 48.8 (CH, Cα), 17.8 (CH_3_), (CO: n.r.), [*β*-ala^11^] 35.7 (CH_2_, Cα), 35.9 (CH_2_, C*β*), (CO: n.r.), [_D_-Leu^12^] (Cα: n.r.), 42.0 (CH_2_, C*β*), (Cγ: n.r.), 21.3 (CH_3_), 20.3 (CH_3_), (CO: n.r.), [_D_-Me-*allo*-Ile^13^] (NCH_3_: n.r.), 53.8 (CH, Cα), 36.1 (CH, C*β*), 26.1 (CH_2_ Cγ), 20.0 (CH_3_), 23.3 (CH_3_), (CO: n.r.); HRESIMS *m*/*z* 1412.91000 [M + Na]^+^ (calcd. for C_69_H_123_N_13_O_16_Na, 1412.91084). IR: ν_max_ 3300, 2960, 1630, 1520 cm^−1^.

### 3.4. Determination of Amino Acid Sequence by Tandem MS Analysis

The amino acid sequence of compounds **1**–**5** was determined by means of tandem mass analysis of *seco*-acid methyl ester peptides. Each compound (100 μg) was treated with 7 M ammonia solution in methanol at 100 °C for 1 h and concentrated in vacuo. The obtained products were individually subjected to LC–MS/MS analysis equipped with Inertsil ODS-4 (3.0 ID × 150 mm) using a MeCN-H_2_O gradient 10% to 100% over 10 min and keeping 100% MeCN for 10 min, with 0.1% formic acid.

### 3.5. Total Hydrolysis

Each (100 μg) of compounds **1**–**3** was hydrolyzed with 300 μL of 6 M HCl at 110 °C for 24 h. The hydrolysate was concentrated under inert gas and used as the starting material for amino acid analysis by Marfey’s method.

### 3.6. Partial Hydrolysis

Each (0.5 mg) of compounds **1**–**3** was treated with 300 μL of 30% TFA for 40 min at 110 °C in sealed tube. The residue was concentrated, and the target fragments were isolated through preparative HPLC equipped with Cosmosil^®^ 5C^18^-AR using a MeCN-H_2_O gradient from 10% to 100% over 70 min and hold 100% MeCN for 15 min. After confirmation using tandem mass analysis, the target fragments were further hydrolyzed with 300 μL of 6 M HCl at 110 °C for 24 h. The hydrolysates were concentrated under inert gas and used as the starting materials for amino acid analysis by Marfey’s method.

### 3.7. Amino Acid Analysis by Marfey’s Method

The obtained partial and total hydrolysates were individually mixed with H_2_O (160 μL), saturated NaHCO_3_ (200 μL), and 1% FDAA in acetone (160 μL). The mixture was heated at 40 °C for 30 min. Subsequently, the reaction was quenched with 1 M HCl (200 μL) and concentrated under vacuo. The residue was dissolved with DMSO (400 μL) and passed through membrane filter before injecting to LC–MS.

### 3.8. Antibacterial Activity

Compounds **1**–**6** were tested for antibacterial activity. The assay for all compounds has been performed in triplicate according to the Clinical Laboratory Standards Institute testing standard in a 96-well plate microbroth dilution assay. Compounds **1**–**6** were tested against Gram-negative bacterium (*Escherichia coli* JW5503) and Gram-positive bacteria (*Bacillus cereus* NBRC 15305 and *Kocuria rhizophila* NBRC 12708). After incubation for 22 h (30 °C), the obtained optical density was recorded using Tecan infinite^®^ M200 plate reader at 600 nm to determine percent growth inhibition.

### 3.9. Preferential Cytotoxic Activity Assay against MIA PaCa-2, HepG2, and MCF-7 Cell Lines

Preferential cytotoxicity of the isolated theonellapeptolide compounds **1**–**6** was determined according to the procedure described previously [18]. Briefly, MIA PaCa-2, HepG2 and MCF-7 Cells (2 × 10^4^ cells/well) were seeded in 96-well plates and incubated in fresh DMEM. The cells were washed with Dulbecco’s phosphate-buffered saline (PBS) before the medium was replaced with DMEM or NDM containing serial dilutions of the test samples. After 24 h of incubation, the cells were washed with PBS, and 100 μL of DMEM containing 10% WST-8 cell counting kit solution was added to each well. After 3 h of incubation, the absorbance was measured at 450 nm. Cell viability was calculated from the mean values for three wells. The preferential cytotoxicity was expressed as the concentration at which 50% of cells died preferentially in NDM (PC50). 

Pancreatic cancer cells (MIA PaCa-2 cells) were kindly provided by Dr. Suresh Awale, an Associate Professor at Institute of Natural Medicine, University Toyama, Toyama, Japan. MIA PaCa-2 (RBRC-RCB2094), human pancreatic cancer cell lines were purchased from Riken BRC cell bank.

### 3.10. Morphological Analysis

Morphological analysis of MIA PaCa-2 cells after treatment with **4** at different concentrations was determined based on a procedure described previously [18].

### 3.11. Colony Formation Assay

Colony Formation inhibitory effect of **4** at different concentrations against MIA PaCa-2 cells was determined as described previously [19,20].

### 3.12. Live Imaging

Real-time anti-austerity potential tracking anti-pancreatic cancer activity of **4** by showing morphological changes in MIA PaCa-2 cells under NDM was monitored using a Nikon sCMOS camera Oxford instrument, Hamamatsu Photonics/Ti-E, for 24 h. The Movies S1 and S2 (Supporting information) were created by capturing the images every 10 min by rotating at several fixed positions in the dish under the phase contrast mode on a sCMOS camera imaging system for 24 h. An inverted microscope (Ti-E, Nikon) equipped with a Plan Apo VC X 20 objective lens (NA 0.75, Nikon) and micro scanning stage (BI XY stage, Chuo Precision Industrial Co., Ltd.) was used to observe all events in lining cells maintained at 37 °C with a continuous supply of 95% air and 5% carbon dioxide by using a stage-top incubator (INUBG2TF-WSKM, Tokai Hit). Images were taken by scientific Complementary Metal Oxide Semiconductor (sCMOS) camera (Zyla 5.5, Oxford Instruments).

## 4. Conclusions

In conclusion, in this study, we have successfully isolated three new theonellapeptolide II members namely IIb (**1**), IIa (**2**), and IIc (**3**) from the marine sponge *Theonella swinhoei*. Compounds **1** and **2** are the first members of theonellapeptolide characterized by the present of _D_-amino acids in the position 6, whereas **3** contains *N*-Me-*β*-Ala^4^ that is exclusively found in the “E” version of theonellapeptolide-type compounds, such as Ie, IIe (**5**), and IIIe. The isolated compounds (**1**–**6**) did not show any antibacterial activity but displayed growth inhibition of the pancreatic cancer cell line MIA PaCa-2 cultured under nutrient-starvation condition. The structure–anti-austerity relationship revealed the presence of *N*-Me-*β*-Ala^4^ and/or *N*-MeIle^6^ residues were responsible for the observed anti-pancreatic activity under NDM as demonstrated in compounds **3**, **4**, **5** and **6**. Furthermore, theonellapeptolide IId (**4**) showed preferential cytotoxicity in live imaging against the MIA PaCa-2 cell line under NDM condition and inhibited colony formation in rich DMEM condition. This study discovers theonellapeptolide-type metabolites as new potential anti-pancreatic cancer agents with selective cytotoxic activity in starvation condition.

## Figures and Tables

**Table 1:** ^1^H (500 MHz) and ^13^C (125 MHz) NMR data of compound **1** (CDCl_3_, *δ* in ppm).

Pos.	*δ*_C_, Type	*δ*_H_, (Mult)	Pos.	*δ*_C_, Type	*δ*_H_, (Mult)	Pos.	*δ*_C_, Type	*δ*_H_, (Mult)
Methoxy acetyl			_D_-Leucine^5^			C2	62.3, CH	4.65, (m)
C1	170.1, C		C1	n.r., C		C3	26.7, CH	2.22, (m)
C2	71.5, CH_2_	3.89, (m)	C2	47.7, CH	4.89, (m)	C4	18.6, CH_3_	0.75, (m)
C3	70.2, CH_3_	3.59, (m)	C3	41.0, CH_2_	a: 1.32, (m)	C5	19.6, CH_3_	0.90, (m)
_L_-Valine^1^					b: 1.44, (m)	NMe	31.0, CH_3_	2.99, (m)
C1	173.1, C		C4	24.6, CH	1.49, (m)	_L_-Alanine^10^		
C2	53.9, CH	4.72, (m)	C5	23.0, CH_3_	0.85, (m)	C1	172.7, C	
C3	30.9, CH	1.99, (m)	C6	23.1, CH_3_	0.88, (m)	C2	48.7, CH	4.22, (m)
C4	19.3, CH_3_	0.90, (m)	NH		7.57, (m)	C3	17.2, CH_3_	1.15, (m)
C5	17.3, CH_3_	0.85, (m)	_D_-Methyl Valine^6^			NH		7.45, (m)
NH		7.20, (m)	C1	170.4, C		*β*-Alanine^11^		
_D_-Methyl Leucine^2^			C2	62.1, CH	4.72, (m)	C1	171.4, C	
C1	171.2, C		C3	25.7, CH	2.15, (m)	C2	34.9, CH_2_	a: 2.38, (m)
C2	54.9, CH	5.08, (m)	C4	19.8, CH_3_	0.91, (m)			b: 2.29, (m)
C3	37.1, CH_2_	a: 1.58, (m)	C5	18.8, CH_3_	0.75, (m)	C3	36.2, CH_2_	a: 3.43, (m)
		b: 1.72, (m)	NMe	31.3, CH_3_	3.01, (m)			b: 3.35, (m)
C4	24.8, CH	1.35, (m)	*β*-Alanine^7^			NH		7.52, (m)
C5	21.1, CH_3_	0.82, (m)	C1	172.5, C		_D_-Leucine^12^		
C6	21.5, CH_3_	0.87, (m)	C2	35.8, CH_2_	a: 2.36, (m)	C1	173.1, C	
NMe	30.8, CH_3_	3.05, (m)			b: n.r.	C2	47.8, CH	4.92, (m)
_L_-Threonine^3^			C3	36.0, CH_2_	a: 3.60, (m)	C3	41.6, CH_2_	a: 1.28, (m)
C1	168.2, C				b: 3.19, (m)			b: 1.43, (m)
C2	55.7, CH	4.39, (m)	NH		7.52, (m)	C4	24.6, CH	1.50, (m)
C3	70.2, CH	5.08, (m)	_D_-*allo* Isoleucine^8^			C5	23.1, CH_3_	0.83, (m)
C4	15.9, CH_3_	1.05, (m)	C1	173.7, C		C6	23.2, CH_3_	0.89, (m)
NH		7.59, (m)	C2	60.2, CH	4.80, (m)	NH		7.78, (m)
*β*-Alanine^4^			C3	24.8, CH	2.05, (m)	_D_-Methyl *allo* Isoleucine^13^		
C1	171.3, C		C4	24.6, CH	a: 1.24, (m)	C1	173.09, C	
C2	35.8, CH_2_	a: 2.46, (m)			b: 0.96, (m)	C2	54.7, CH	5.15, (m)
		b: 2.19, (m)	C5	15.2, CH_3_	0.91, (m)	C3	36.6, CH	1.71, (m)
C3	36.0, CH_2_	a: 3.47, (m)	C6	15.1, CH_3_	0.83, (m)	C4	26.2, CH	a: 1.38, (m)
		b: 3.26, (m)	NH		7.83, (m)			b: 1.16, (m)
NH		7.25, (m)	_L_-Methyl Valine^9^			C5	17.9, CH_3_	0.88, (m)
			C1	169.5, C		C6	17.2, CH_3_	0.85, (m)
						NMe	30.6, CH_3_	2.98, (m)

n.r.: not resolved due to overlapped signals.

## Data Availability

Data are contained within this article and supplementary files.

## Supplementary Materials

The supporting information can be downloaded at: https://www.mdpi.com/article/10.3390/md20110661/s1, Materials and Methods, Figure S1: The work scheme of isolation and purification of **1**–**5**; Figure S2: COSY spectrum of **1** showing the presence of conformers; Figure S3: Tandem mass analyses of *seco*-acid methyl ester peptide (**1a**, **2a**, and **4a**); Figure S4: MS/MS spectrum of *seco*-acid methyl ester peptide of **1a**; Figure S5: MS/MS/MS spectrum of fragment ion y_8_ of **1a**; Figure S6: MS/MS/MS spectrum of fragment ion b_5_ of **1a**; Figure S7: MS/MS/MS spectrum of fragment ion b_2_ of **1a**; Figure S8: MS/MS spectrum of *seco*-acid methyl ester peptide of **2a**; Figure S9: MS/MS/MS spectrum of fragment ion y_8_ of **2a**; Figure S10: MS/MS/MS spectrum of fragment ion b_5_ of **2a**; Figure S11: MS/MS spectrum of *seco*-acid methyl ester peptide of **3a**; Figure S12: MS/MS/MS spectrum of fragment ion y_10_ of **3a**; Figure S13: MS/MS/MS spectrum of fragment ion b_3_ of **3a**; Figure S14: MS/MS spectrum of *seco*-acid methyl ester peptide of **4a**; Figure S15: MS/MS/MS spectrum of fragment ion y_8_ of **4a**; Figure S16: MS/MS/MS spectrum of fragment ion b_5_ of **4a**; Figure S17: MS/MS spectrum of *seco*-acid methyl ester peptide of **5a**; Figure S18: MS/MS/MS spectrum of fragment ion y_10_ of **5a**; Figure S19: MS/MS/MS spectrum of fragment ion b_3_ of **5a**; Figure S20: ^1^H NMR spectrum (CDCl_3_, 500 MHz) of compound **1**; Figure S21: ^13^C NMR spectrum (CDCl_3_, 125 MHz) of compound **1**; Figure S22: COSY NMR spectrum (CDCl_3_, 500 MHz) of compound **1**; Figure S23: HSQC NMR spectrum (CDCl_3_, 500 MHz) of compound **1**; Figure S24: HMBC NMR spectrum (CDCl_3_, 500 MHz) of compound **1**; Figure S25: ROESY NMR spectrum (CDCl_3_, 500 MHz) of compound **1**; Figure S26: ^1^H NMR spectrum (CDCl_3_, 500 MHz) of compound **2**; Figure S27: ^13^C NMR spectrum (CDCl_3_, 125 MHz) of compound **2**; Figure S28: COSY NMR spectrum (CDCl_3_, 500 MHz) of compound **2**; Figure S29: HSQC NMR spectrum (CDCl_3_, 500 MHz) of compound **2**; Figure S30: HMBC NMR spectrum (CDCl_3_, 500 MHz) of compound **2**; Figure S31: ROESY NMR spectrum (CDCl_3_, 500 MHz) of compound **2**; Figure S32: ^1^H NMR spectrum (CDCl_3_, 500 MHz) of compound **3**; Figure S33: ^13^C NMR spectrum (CDCl_3_, 125 MHz) of compound **3**; Figure S34: COSY NMR spectrum (CDCl_3_, 500 MHz) of compound **3**; Figure S35: HSQC NMR spectrum (CDCl_3_, 500 MHz) of compound **3**; Figure S36: HMBC NMR spectrum (CDCl_3_, 500 MHz) of compound **3**; Figure S37: ROESY NMR spectrum (CDCl_3_, 500 MHz) of compound **3**; Figure S38: MS/MS analysis of **1b** and its Marfey’s analysis; Figure S39: Marfey’s analysis of **1**; Figure S40: MS/MS analysis of **2b** and its Marfey’s analysis; Figure S41: Marfey’s analysis of **2**; Figure S42: MS/MS analysis of **3b** and its Marfey’s analysis; Figure S43: Marfey’s analysis of **3**; Table S1: List of obtained fragment ions from MS/MS analysis of **1a**; Table S2: List of obtained fragment ions from MS/MS/MS analysis of y_8_-**1a**; Table S3: List of obtained fragment ions from MS/MS analysis of **2a**; Table S4: List of obtained fragment ions from MS/MS/MS analysis of y_8_-**2a**; Table S5: List of obtained fragment ions from MS/MS analysis of **3a**; Table S6: List of obtained fragment ions from MS/MS/MS analysis of y_10_-**3a**; Table S7: List of obtained fragment ions from MS/MS analysis of 4a; Table S8: List of obtained fragment ions from MS/MS/MS analysis of y_8_-**4a**; Table S9: List of obtained fragment ions from MS/MS analysis of **5a**; Table S10: List of obtained fragment ions from MS/MS/MS analysis of y_10_-**5a**; Table S11: Biological activity of **1**–**6**. Movie S1: Real-time movie on the effect of 10 μM of **4** compared to control (untreated) on MIA PaCa-2 cells with one frame per 15 min for 24 h (AVI). Movie S2: Multi positions real-time movie on the effect of 10 μM of **4** compared to control (untreated) on MIA PaCa-2 cells with one frame per 15 min for 24 h (AVI) (three representative positions).

## Abbreviations

Ac, acetyl; Ala, alanine; COSY, homonuclear correlation spectroscopy; DMSO, dimethyl sulfoxide; ESI/MS, electrospray ionization/mass spectrometry; FDAA, 1-fluoro-2-4-dinitrophenyl-5-_L_-alanine amide; HMBC, heteronuclear multiple bond correlation; HPLC, high-performance liquid chromatography; HRESIMS, high-resolution electrospray ionization mass spectrometry; HSQC, heteronuclear single-quantum coherence; Ile, isoleucine; LC/MS, liquid chromatography/mass spectrometry; Leu, leucine; M, molar concentration; MeO, methoxy; MeOH, methanol; NMR, nuclear magnetic resonance; ROESY, rotating frame overhauser enhancement spectroscopy; Thr, threonine; TLC, thin-layer chromatography; Tlpd, theonellapeptolide; Val, valine; *β*-Ala, *β*-alanine. EB, ethidium bromide; AO, acridine orange; NDM, nutrient-deprived medium; DMEM, Dulbecco’s Modified Eagle Medium.
