# Supplementary material for: New Theonellapeptolides from Indonesian Marine Sponge Theonella swinhoei as Anti-Austerity Agents
Source: Mar Drugs. 2022 Oct 25;20(11):661. doi: 10.3390/md20110661 (PMC9697519; doi:10.3390/md20110661)
Supplement: Supplementary file 1 [file marinedrugs-20-00661-s001.zip › marinedrugs-1963006-supplementary.pdf]

Supplementary Information

New Theonellapeptolides from Indonesian Marine Sponge *Theonella swinhoei* as Anti-austerity Agents

Jabal Rahmat Haedar [1], Agustinus Robert Uria [1,2], Subehan Lallo [3], Dya Fita Dibwe [4,5,6*], and Toshiyuki Wakimoto [1,2*]

[1] Faculty of Pharmaceutical Sciences, Hokkaido University, Kita 12, Nishi 6, Sapporo 060-0812, Japan
[2] Global Station for Biosurfaces and Drug Discovery, Global Institution for Collaborative Research and Education (GI-CoRE), Hokkaido University, Kita 12, Nishi 6, Sapporo 060-0812, Japan
[3] Faculty of Pharmacy, Hasanuddin University, JI. Perintis Kemerdekaan Km. 10, Makassar 90245, Indonesia
[4] Faculty of Health Sciences, Hokkaido University, Kita-12, Nishi-5, Kita-Ku, Sapporo 060-0812, Japan
[5] Faculty of Sciences, Department of Chemistry, University of Kinshasa, Kinshasa P.O. Box 190 KIN XI, Congo
[6] Department of Biotechnology and Natural Products, Research Center for Applied Sciences and Technologies, 106 Boulevard 30 Juin, Kinshasa P.O. Box 8401 Kinshasa-1, Congo
* Correspondence: dibwedf@hs.hokudai.ac.jp or eddy.dibwe@unikin.ac.cd (D.F.D.); wakimoto@pharm.hokudai.ac.jp (T.W.)

Table of content

**Figure S1**	The work scheme of isolation and purification of **1–5** ……………………………..	4
**Figure S2**	COSY spectrum of **1** showing the presence of conformers ………………………...	5
**Figure S3**	Tandem mass analyses of *seco*-acid methyl ester peptide (**1a**, **2a**, and **4a**) ………..	6
**Figure S4**	MS/MS spectrum of *seco*-acid methyl ester peptide of **1a** …………………………	7
**Figure S5**	MS/MS/MS spectrum of fragment ion y_8 of **1a** ……………………………………	8
**Figure S6**	MS/MS/MS spectrum of fragment ion b_5 of **1a** ……………………………………	9
**Figure S7**	MS/MS/MS spectrum of fragment ion b_2 of **1a** ……………………………………	10
**Figure S8**	MS/MS spectrum of *seco*-acid methyl ester peptide of **2a** …………………………	11
**Figure S9**	MS/MS/MS spectrum of fragment ion y_8 of **2a** ……………………………………	12
**Figure S10**	MS/MS/MS spectrum of fragment ion b_5 of **2a** ……………………………………	13
**Figure S11**	MS/MS spectrum of *seco*-acid methyl ester peptide of **3a** …………………………	14
**Figure S12**	MS/MS/MS spectrum of fragment ion y_{10} of **3a** …………………………………...	15
**Figure S13**	MS/MS/MS spectrum of fragment ion b_3 of **3a** …………………………………...	16
**Figure S14**	MS/MS spectrum of *seco*-acid methyl ester peptide of **4a** …………………………	17
**Figure S15**	MS/MS/MS spectrum of fragment ion y_8 of **4a** …………………………………...	18
**Figure S16**	MS/MS/MS spectrum of fragment ion b_5 of **4a** …………………………………...	19
**Figure S17**	MS/MS spectrum of *seco*-acid methyl ester peptide of **5a** …………………………	20
**Figure S18**	MS/MS/MS spectrum of fragment ion y_{10} of **5a** …………………………………...	21
**Figure S19**	MS/MS/MS spectrum of fragment ion b_3 of **5a** …………………………………...	22
**Figure S20**	^{1}H NMR spectrum ($CDCl_3$, 500 MHz) of compound **1** ……………………………	23
**Figure S21**	^{13}C NMR spectrum ($CDCl_3$, 125 MHz) of compound **1** …………………………...	23
**Figure S22**	COSY NMR spectrum ($CDCl_3$, 500 MHz) of compound **1** ………………………	24
**Figure S23**	HSQC NMR spectrum ($CDCl_3$, 500 MHz) of compound **1** ………………………..	24
**Figure S24**	HMBC NMR spectrum ($CDCl_3$, 500 MHz) of compound **1** ……………………….	25
**Figure S25**	ROESY NMR spectrum ($CDCl_3$, 500 MHz) of compound **1** ………………………	25
**Figure S26**	^{1}H NMR spectrum ($CDCl_3$, 500 MHz) of compound **2** ……………………………	26
**Figure S27**	^{13}C NMR spectrum ($CDCl_3$, 125 MHz) of compound **2** …………………………...	26
**Figure S28**	COSY NMR spectrum ($CDCl_3$, 500 MHz) of compound **2** ………………………..	27
**Figure S29**	HSQC NMR spectrum ($CDCl_3$, 500 MHz) of compound **2** ………………………..	27
**Figure S30**	HMBC NMR spectrum ($CDCl_3$, 500 MHz) of compound **2** ……………………….	28
**Figure S31**	ROESY NMR spectrum ($CDCl_3$, 500 MHz) of compound **2** ………………………	28
**Figure S32**	^{1}H NMR spectrum ($CDCl_3$, 500 MHz) of compound **3** ……………………………	29
**Figure S33**	^{13}C NMR spectrum ($CDCl_3$, 125 MHz) of compound **3** …………………………...	29
**Figure S34**	COSY NMR spectrum ($CDCl_3$, 500 MHz) of compound **3** ………………………..	30
**Figure S35**	HSQC NMR spectrum ($CDCl_3$, 500 MHz) of compound **3** ………………………..	30
**Figure S36**	HMBC NMR spectrum ($CDCl_3$, 500 MHz) of compound **3** ……………………….	31
**Figure S37**	ROESY NMR spectrum ($CDCl_3$, 500 MHz) of compound **3** ………………………	31
**Figure S38**	MS/MS analysis of **1b** and its Marfey's analysis …………………………………..	32
**Figure S39**	Marfey's analysis of **1** …………………………………………………………...	33
**Figure S40**	MS/MS analysis of **2b** and its Marfey's analysis …………………………………..	34
**Figure S41**	Marfey's analysis of **2** …………………………………………………………...	35
**Figure S42**	MS/MS analysis of **3b** and its Marfey's analysis …………………………………..	36
**Figure S43**	Marfey's analysis of **3** …………………………………………………………...	37
**Table S1**	List of obtained fragment ions from MS/MS analysis of **1a** ……………………….	38
**Table S2**	List of obtained fragment ions from MS/MS/MS analysis of y_8-**1a** ………………	39
**Table S3**	List of obtained fragment ions from MS/MS analysis of **2a** ……………………….	40
**Table S4**	List of obtained fragment ions from MS/MS/MS analysis of y_8-**2a** ………………	41
**Table S5**	List of obtained fragment ions from MS/MS analysis of **3a** ……………………….	42
**Table S6**	List of obtained fragment ions from MS/MS/MS analysis of y_{10}-**3a** ……………..	43

**Table S7**	List of obtained fragment ions from MS/MS analysis of **4a** ……………………….	44
**Table S8**	List of obtained fragment ions from MS/MS/MS analysis of y_8-**4a** ……………….	45
**Table S9**	List of obtained fragment ions from MS/MS analysis of **5a** ……………………….	46
**Table S10**	List of obtained fragment ions from MS/MS/MS analysis of y_{10}-**5a** ………………	47
**Table S11**	Biological activity of **1–6**……..………………………………………………………..	48

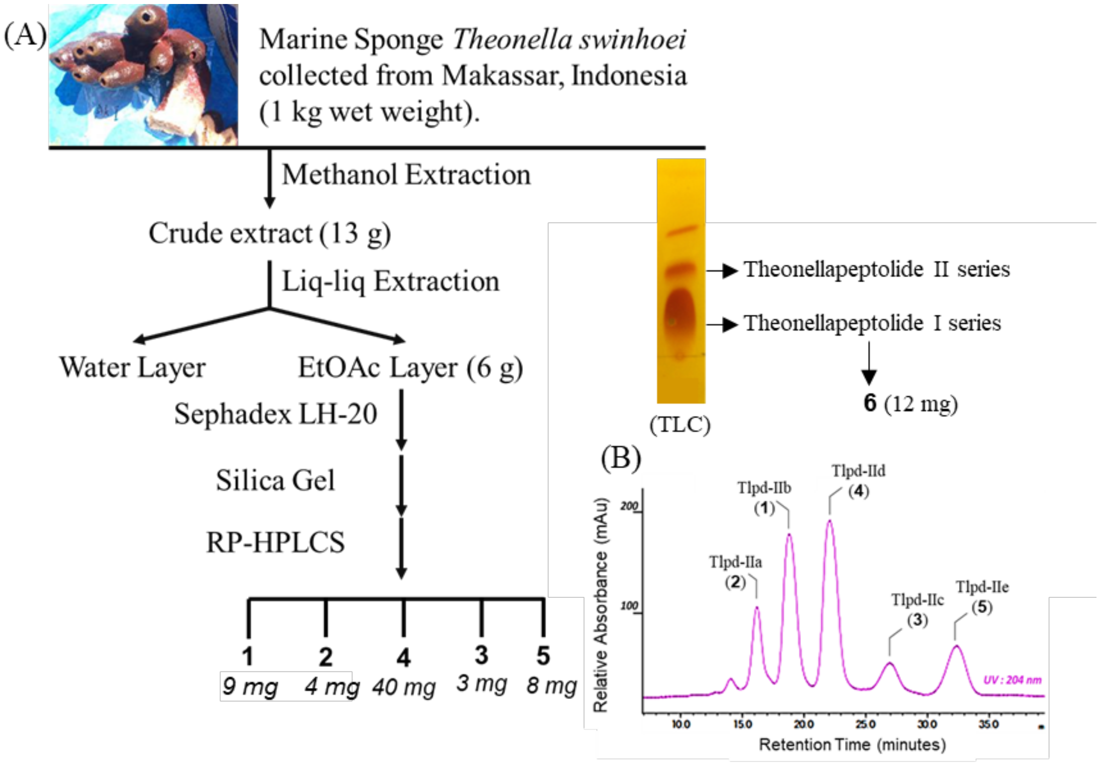

Figure S1. Isolation and purification of five theonellapeptolide-type compounds (**1**, **2**, **3**, **4**, and **5**) from Kondingarengan *Theonella swinhoei*. Thin Layer Chromatography (TLC) analysis of the sponge methanol extract using dragendorff's reagent showed three brown spots. Secondary metabolites corresponding to the second spot were purified, resulting in the isolation of five different compounds (A); HPLC profile of five purified compounds **1–5** (B), in which two of them (**4** and **5**) are known theonellapeptolide IId and IIe, respectively. Three new analogues IIb (**1**), IIa (**2**), and IIc (**3**) were subsequently characterized to determine their chemical structures.

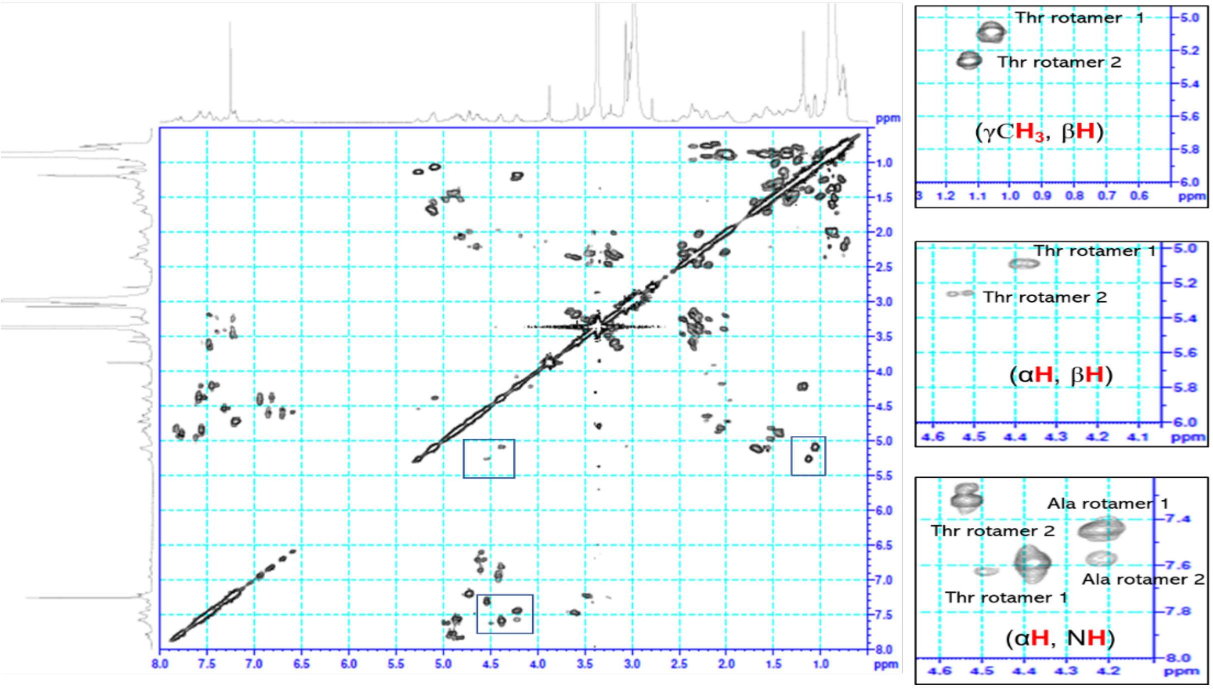

Figure S2. ^{1}H-^{1}H COSY of 2D NMR spectra (500 MHz) of compound **1** in $CDCl_3$ showing threonine and alanine conformers.

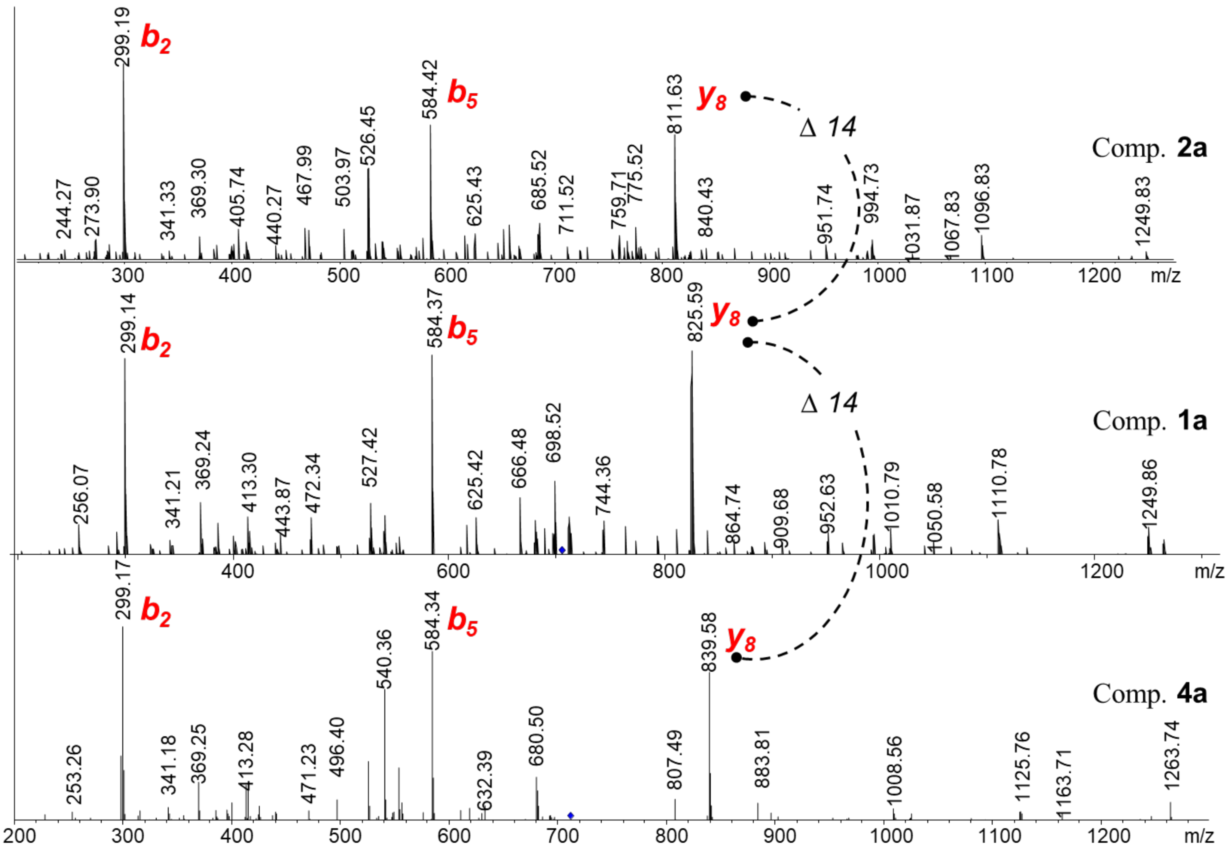

Figure S3. Tandem MS spectra of the *seco*-acid methyl ester peptides **1a** and **2a** compared with originally from known theonellapeptolide IId (**4a**).

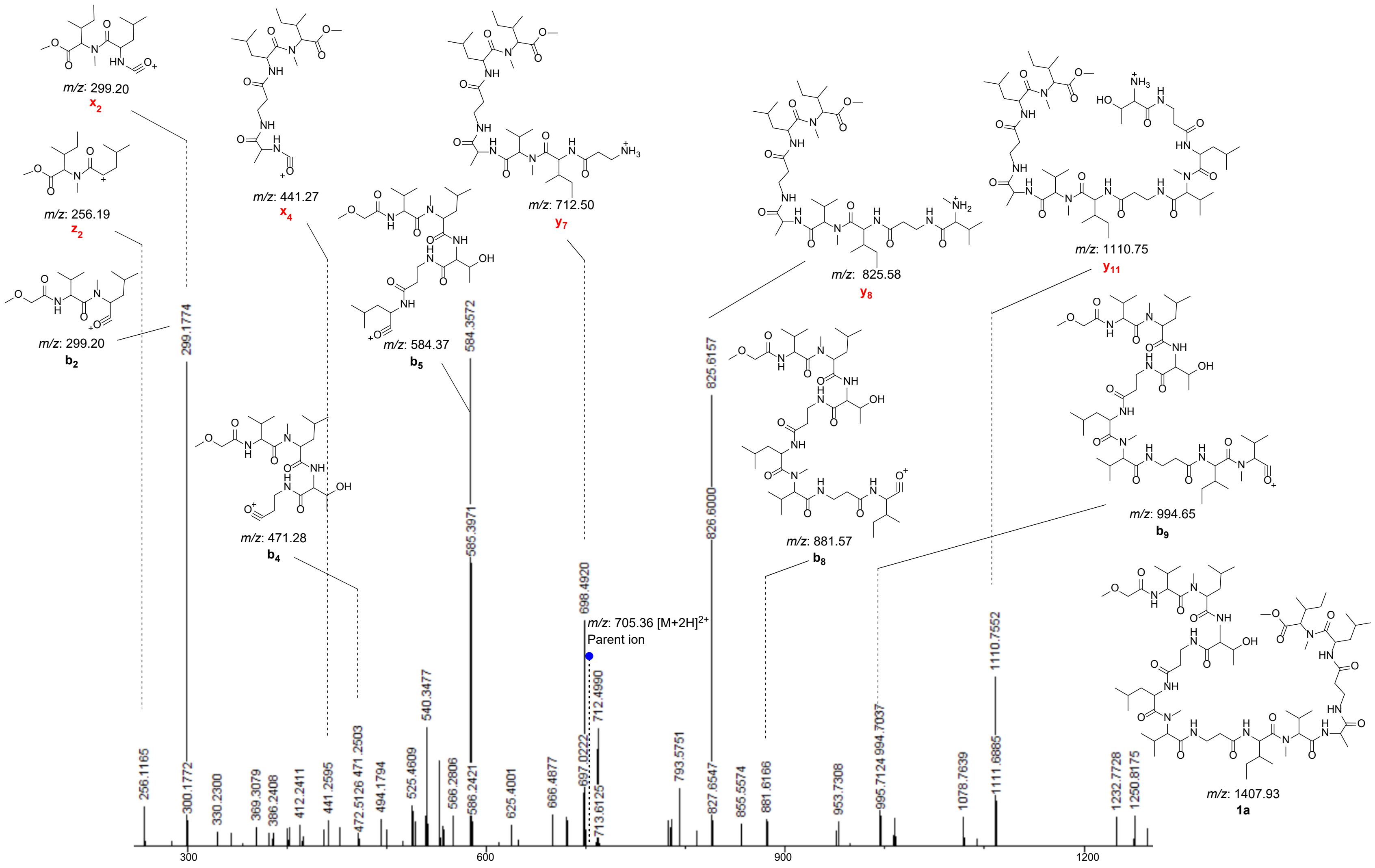

Figure S4. MS/MS spectrum of *seco*-acid methyl ester peptide **1a** *m/z* 705.36 $[M+2H]^{2+}$, (red colored letter denoted fragment ions generated toward C-terminus, black colored letter denoted fragment ions toward N-terminus). The other detected fragment ions are listed in Table S1.

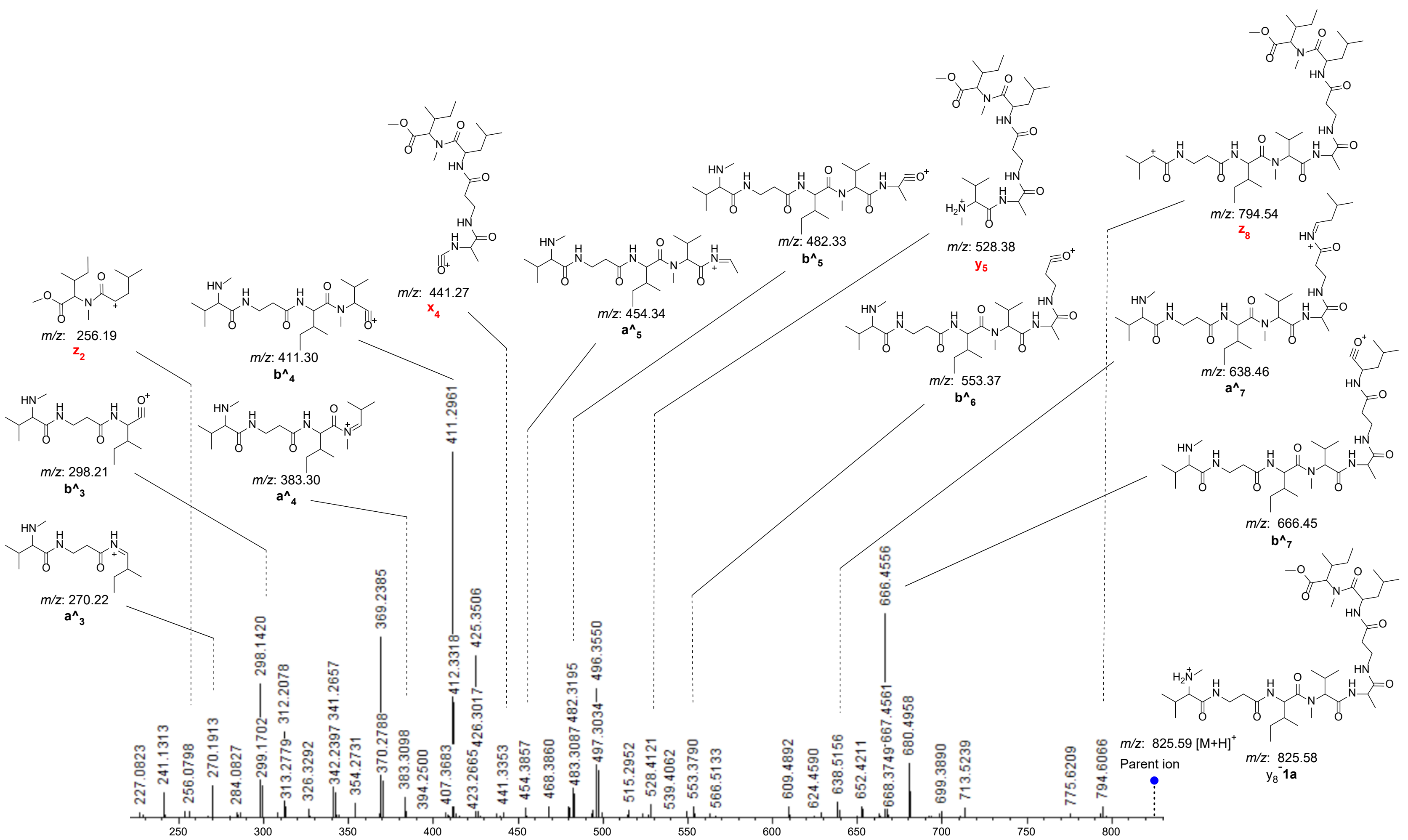

Figure S5. MS/MS/MS spectrum of fragment ion y_8 of **1a**, *m/z* 825.59 $[M+H]^+$ (red colored letter denoted fragment ions generated toward C-terminus, black colored letter denoted fragment ions toward N-terminus). The other detected fragment ions are listed in Table S2.

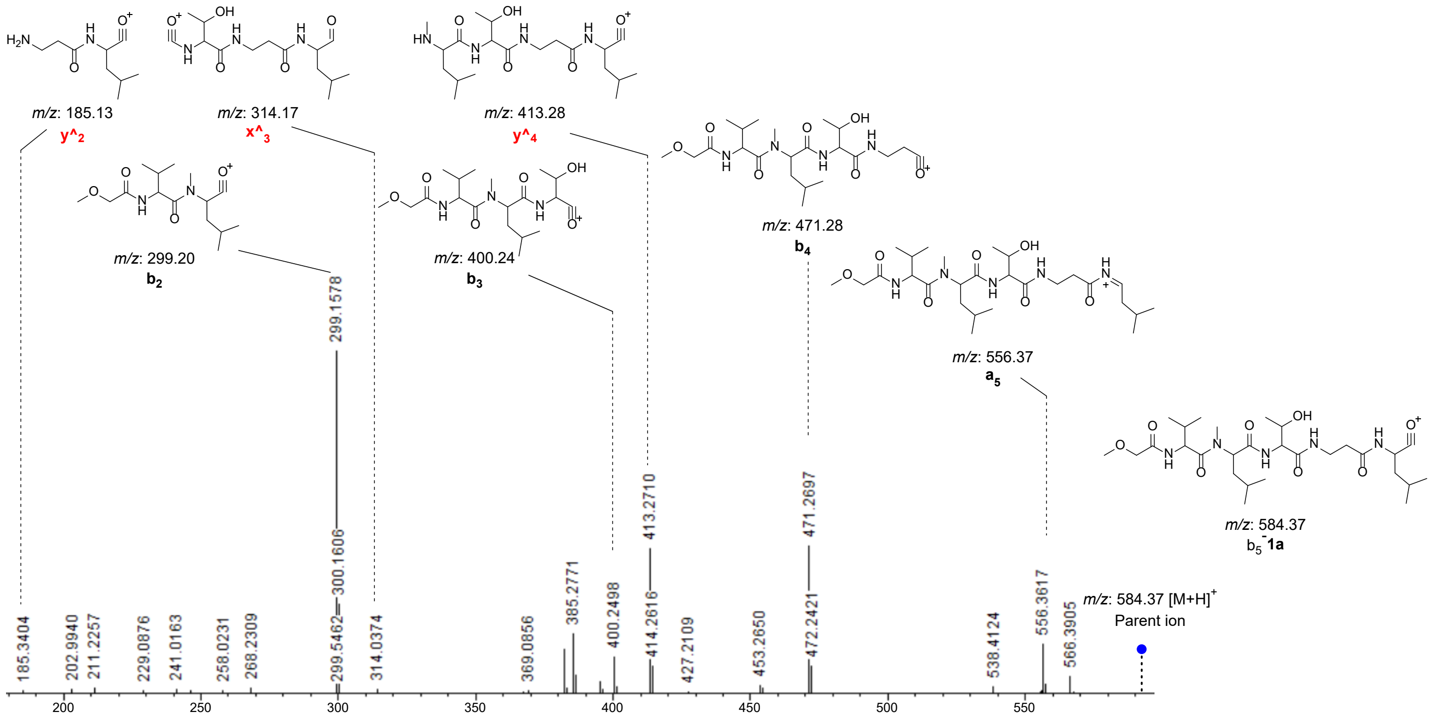

Figure S6. MS/MS/MS spectrum of fragment ion b_5 of **1a**, *m/z* 584.37 $[M+H]^+$ (red colored letter denoted fragment ions generated toward C-terminus; black colored letter denoted fragment ions toward N-terminus).

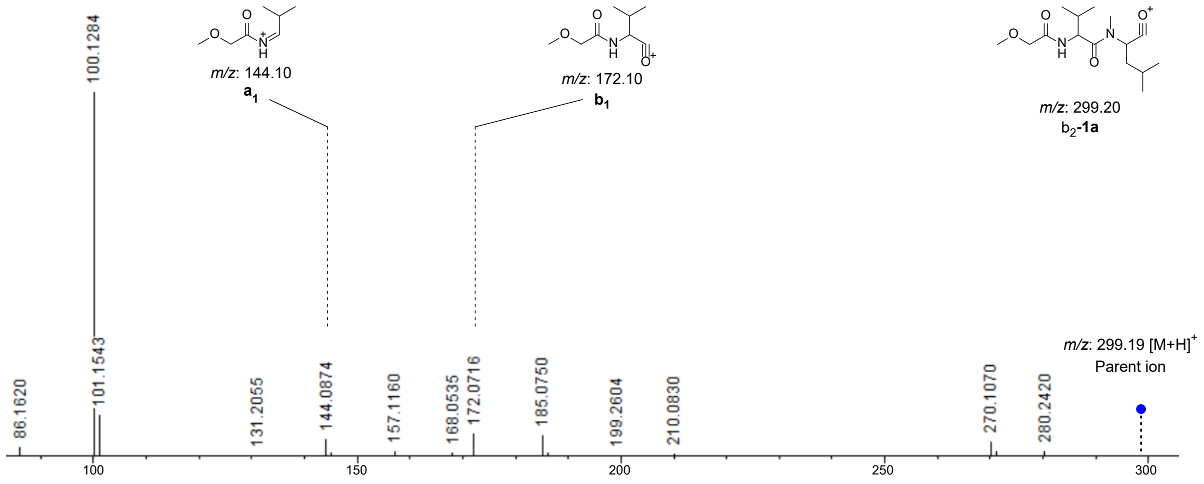

Figure S7. MS/MS/MS spectrum of fragment ion b_2 of **1a**, *m/z* 299.19 $[M+H]^+$ (fragment ions toward N-terminus).

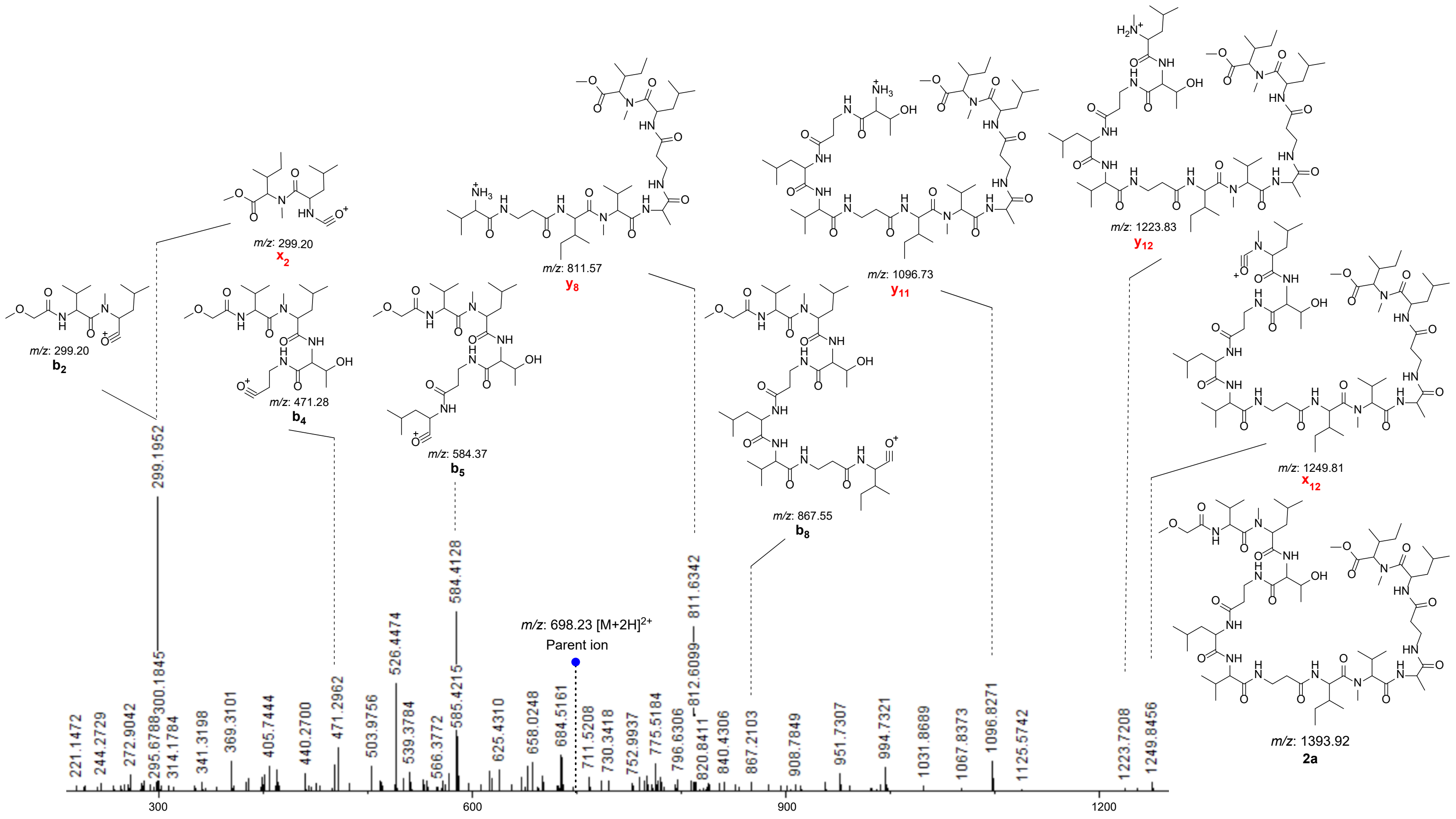

Figure S8. MS/MS spectrum of *seco*-acid methyl ester peptide **2a**, *m/z* 698.23 $[M+2H]^{2+}$, (red colored letter denoted fragment ions generated toward C-terminus, black colored letter denoted fragment ions toward N-terminus). The other detected fragment ions are listed in Table S3.

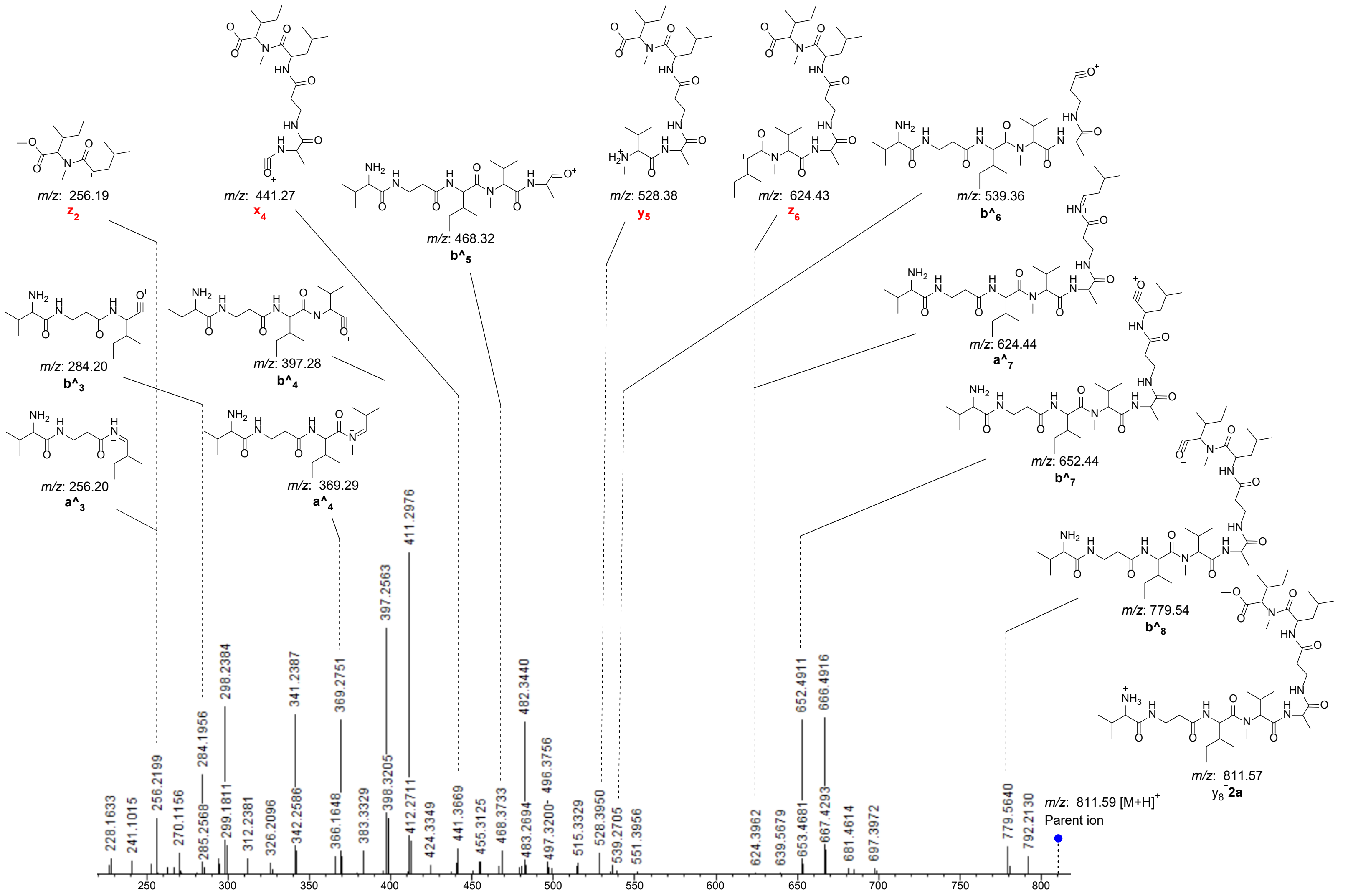

Figure S9. MS/MS/MS spectrum of fragment ion y_8 of **2a**, *m/z* 811.59 $[M+H]^+$, (red colored letter denoted fragment ions generated toward C-terminus, black colored letter denoted fragment ions toward N-terminus). The other obtained fragment ions are listed in Table S4.

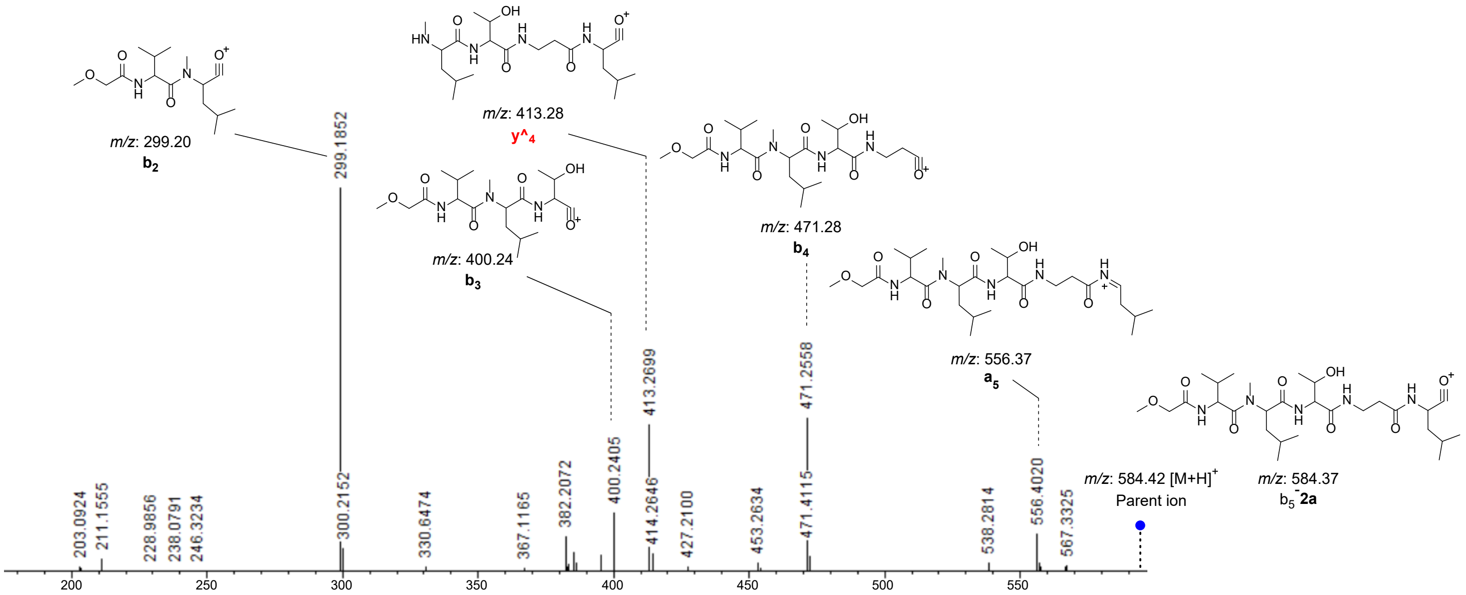

Figure S10. MS/MS/MS spectrum of fragment ion b_5 of **2a**, *m/z* 584.42 $[M+H]^+$, (red colored letter denoted fragment ions generated toward C-terminus, black colored letter denoted fragment ions toward N-terminus).

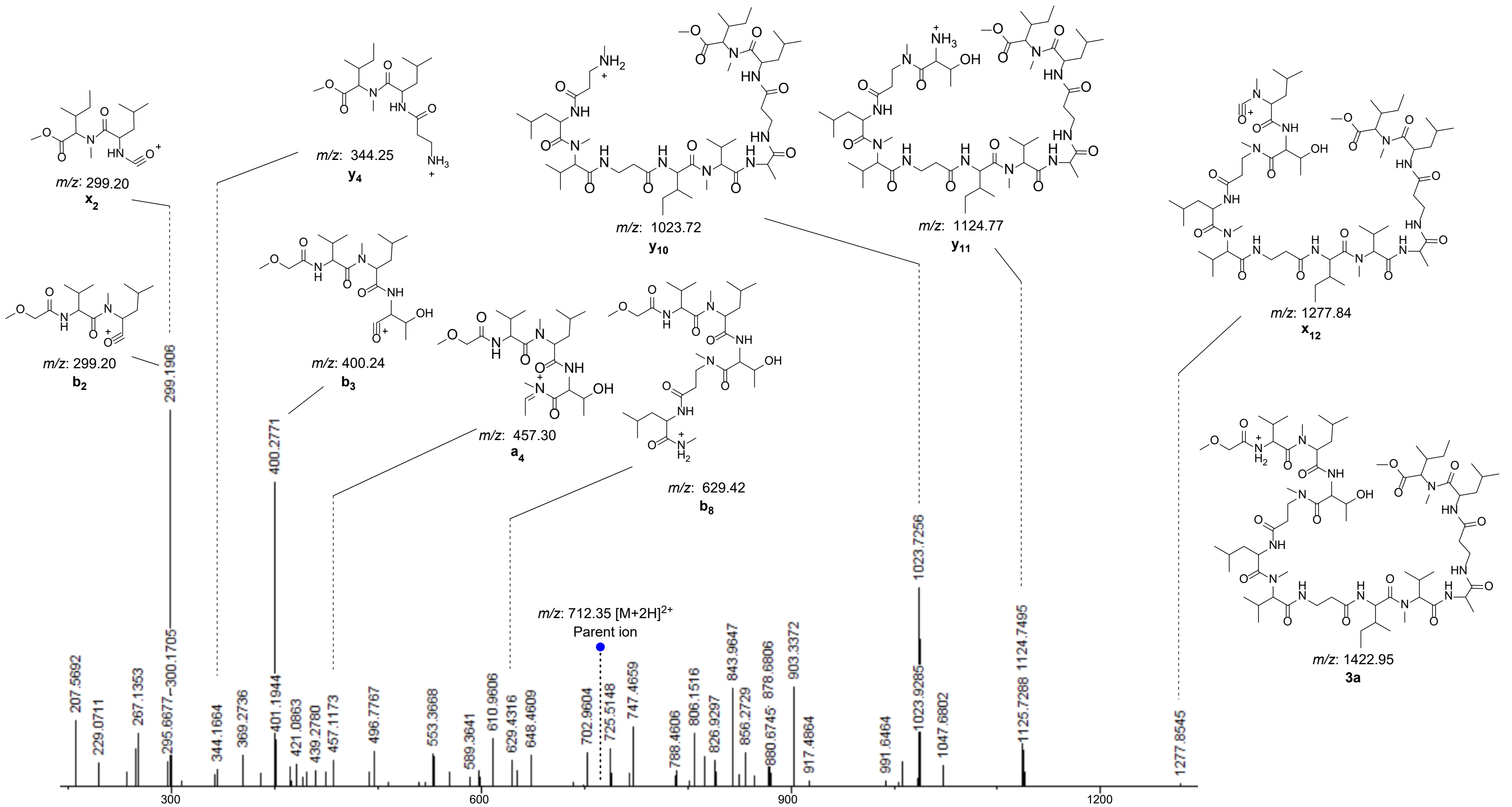

Figure S11. MS/MS spectrum of *seco*-acid methyl ester peptide **3a**, *m/z* 712.35 $[M+2H]^{2+}$, (red colored letter denoted fragment ions generated toward C-terminus, black colored letter denoted fragment ions toward N-terminus). The other detected fragment ions are listed in Table S5.

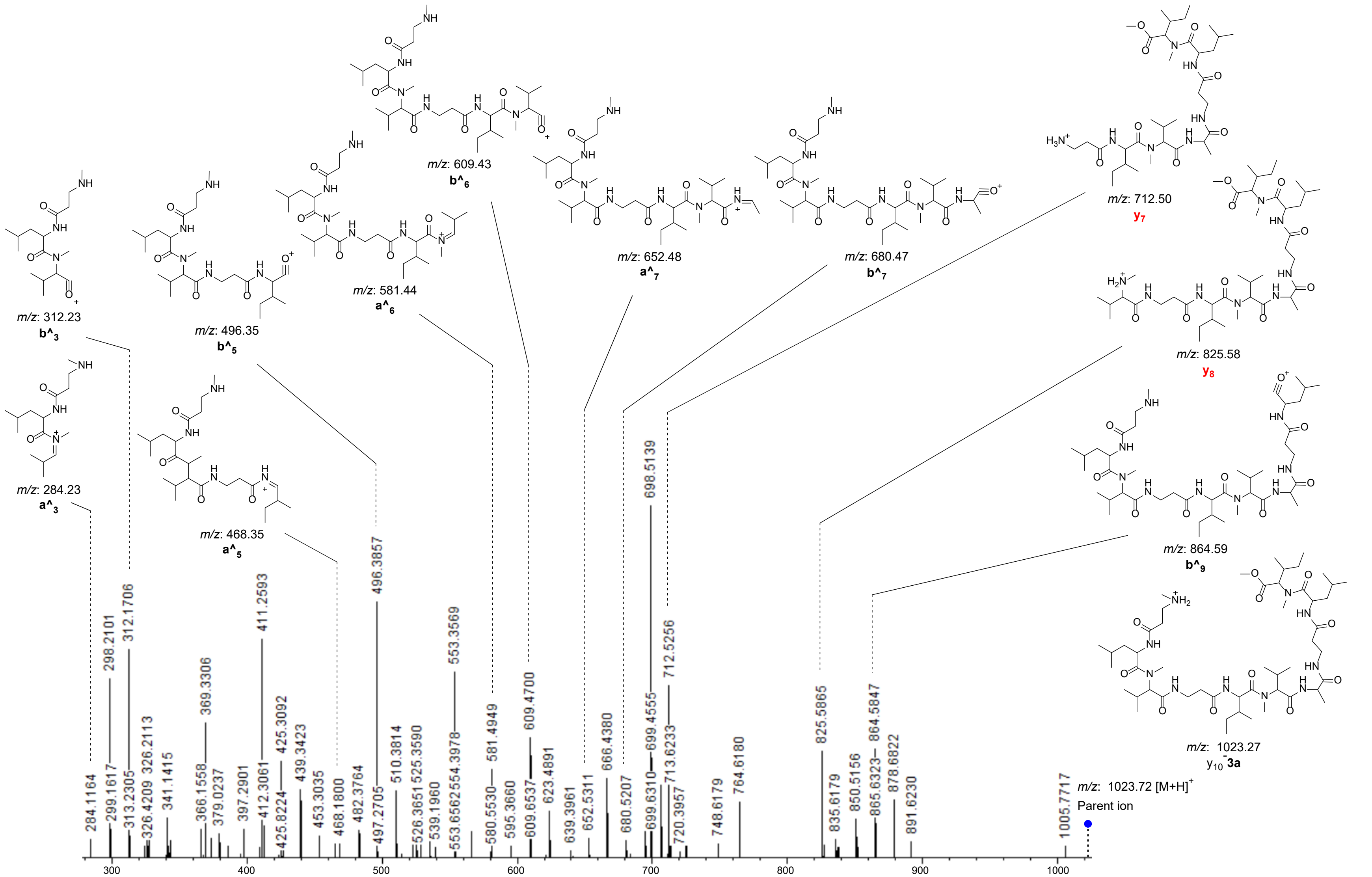

Figure S12. MS/MS/MS spectrum of fragment ion y_{10} of **3a**, *m/z* 1023.27 $[M+H]^+$, (red colored letter denoted fragment ions generated toward C-terminus, black colored letter denoted fragment ions toward N-terminus). The other detected fragment ions are listed in Table S6.

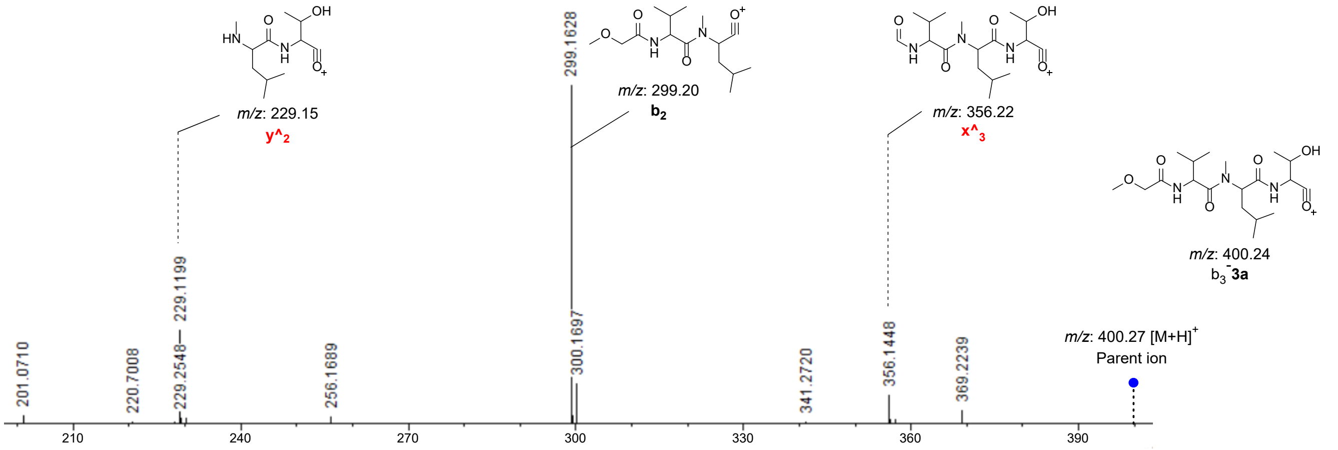

Figure S13. MS/MS/MS spectrum of fragment ion b3 of **3a**, *m/z* 400.42 $[M+H]^+$, (red colored letter denoted fragment ions generated toward C-terminus, black colored letter denoted fragment ions toward N-terminus).

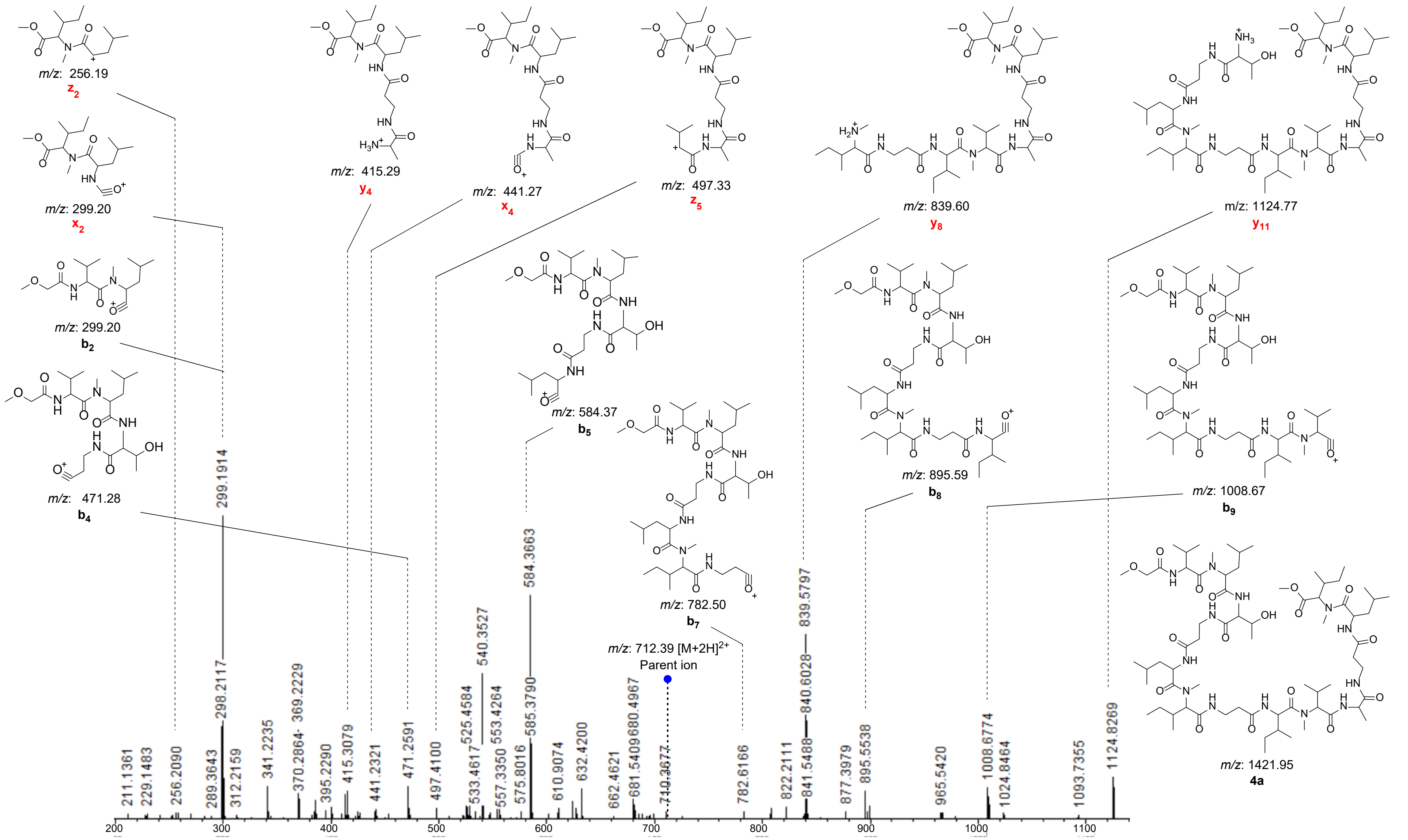

Figure S14. MS/MS spectrum of *seco*-acid methyl ester peptide **4a**, *m/z* 712.39 $[M+2H]^{2+}$, (red colored letter denoted fragment ions generated toward C-terminus, black colored letter denoted fragment ions toward N-terminus). The other detected fragment ions are listed in Table S7.

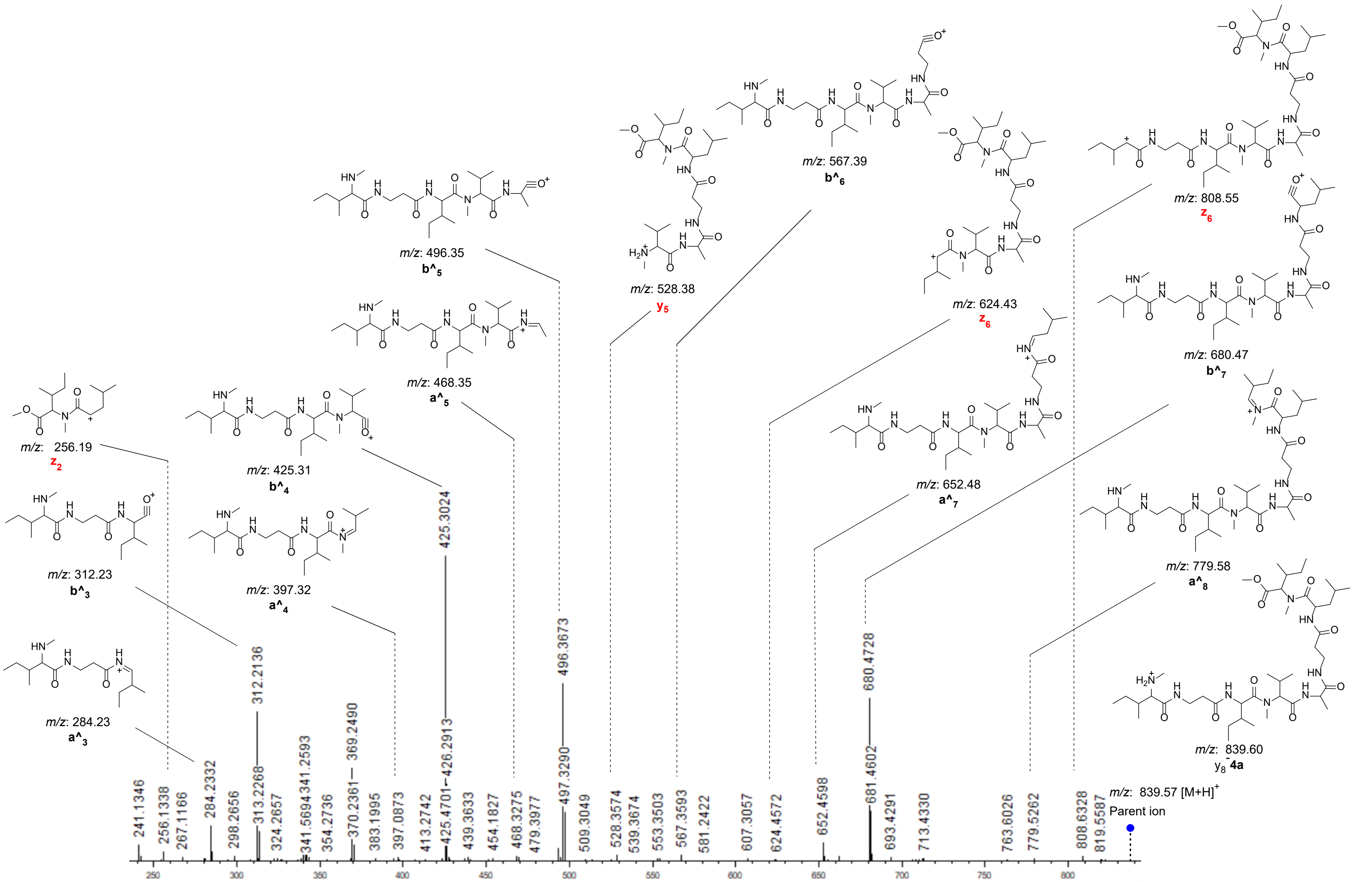

Figure S15. MS/MS/MS spectrum of fragment ion y_8 of **4a**, *m/z* 839.57 $[M+H]^+$, (red colored letter denoted fragment ions generated toward C-terminus, black colored letter denoted fragment ions toward N-terminus). The other detected fragment ions are listed in Table S8.

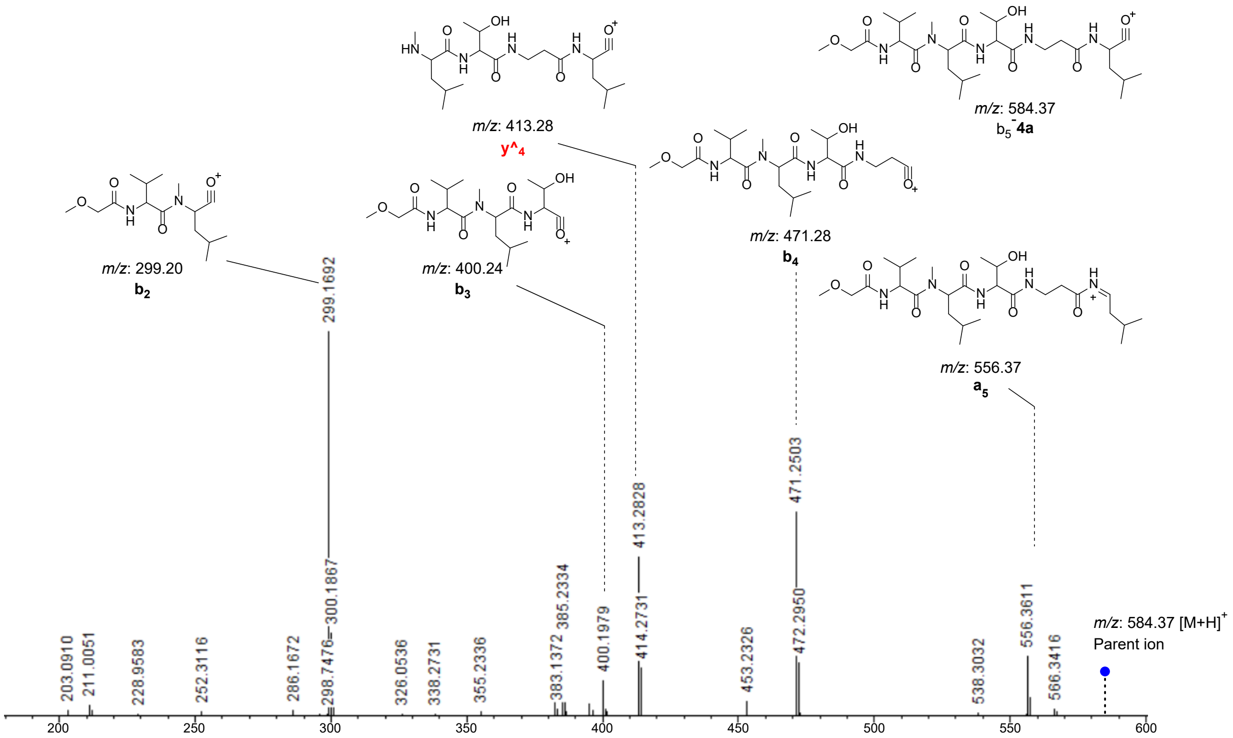

Figure S16. MS/MS/MS spectrum of fragment ion b_5 of **4a**, *m/z* 584.37 $[M+H]^+$, (red colored letter denoted fragment ions generated toward C-terminus, black colored letter denoted fragment ions toward N-terminus).

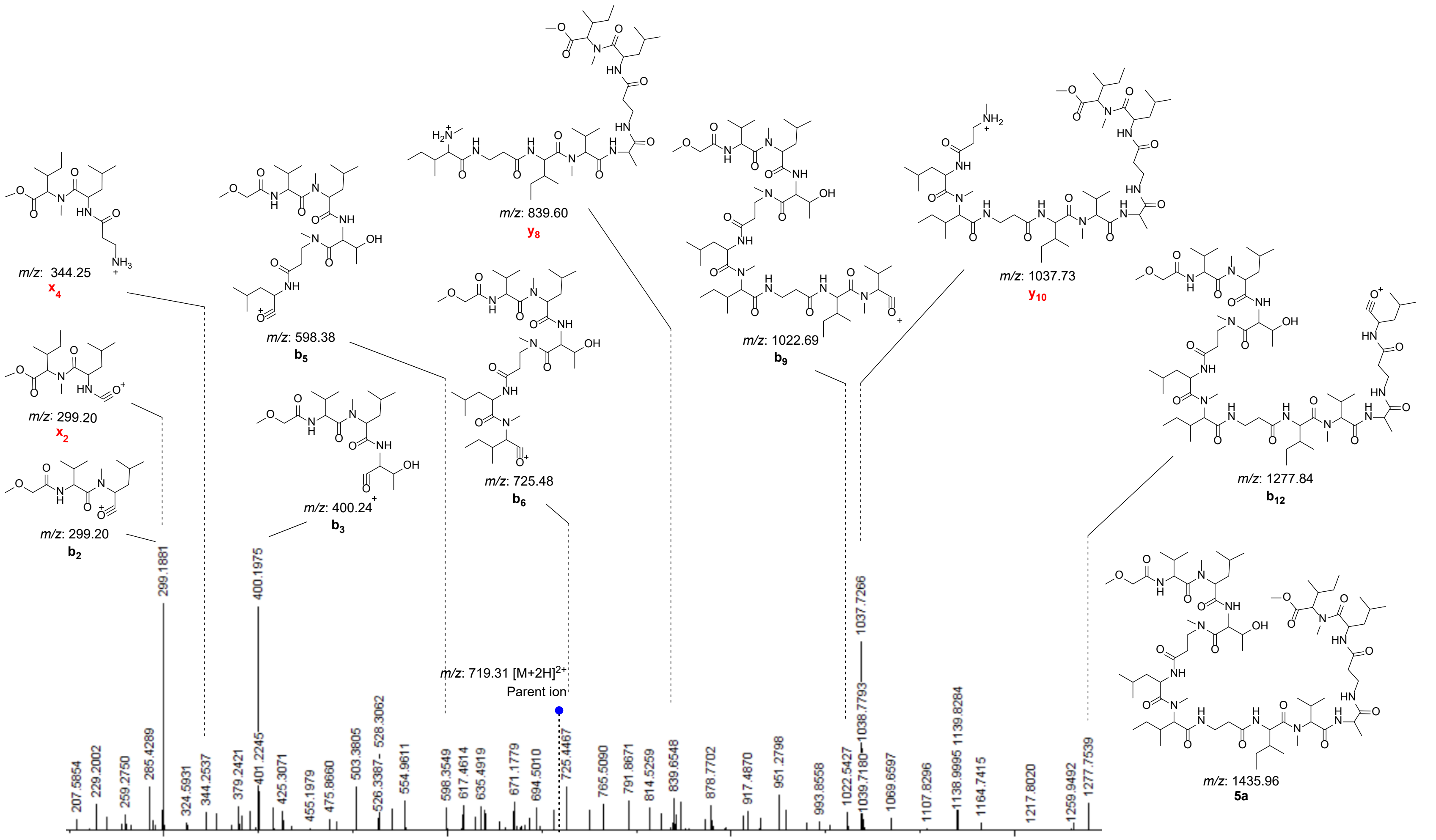

Figure S17. MS/MS spectrum of *seco*-acid methyl ester peptide **5a**, *m/z* 719.31 $[M+2H]^{2+}$, (red colored letter denoted fragment ions generated toward C-terminus, black colored letter denoted fragment ions toward N-terminus). The other detected fragment ions are listed in Table S9.

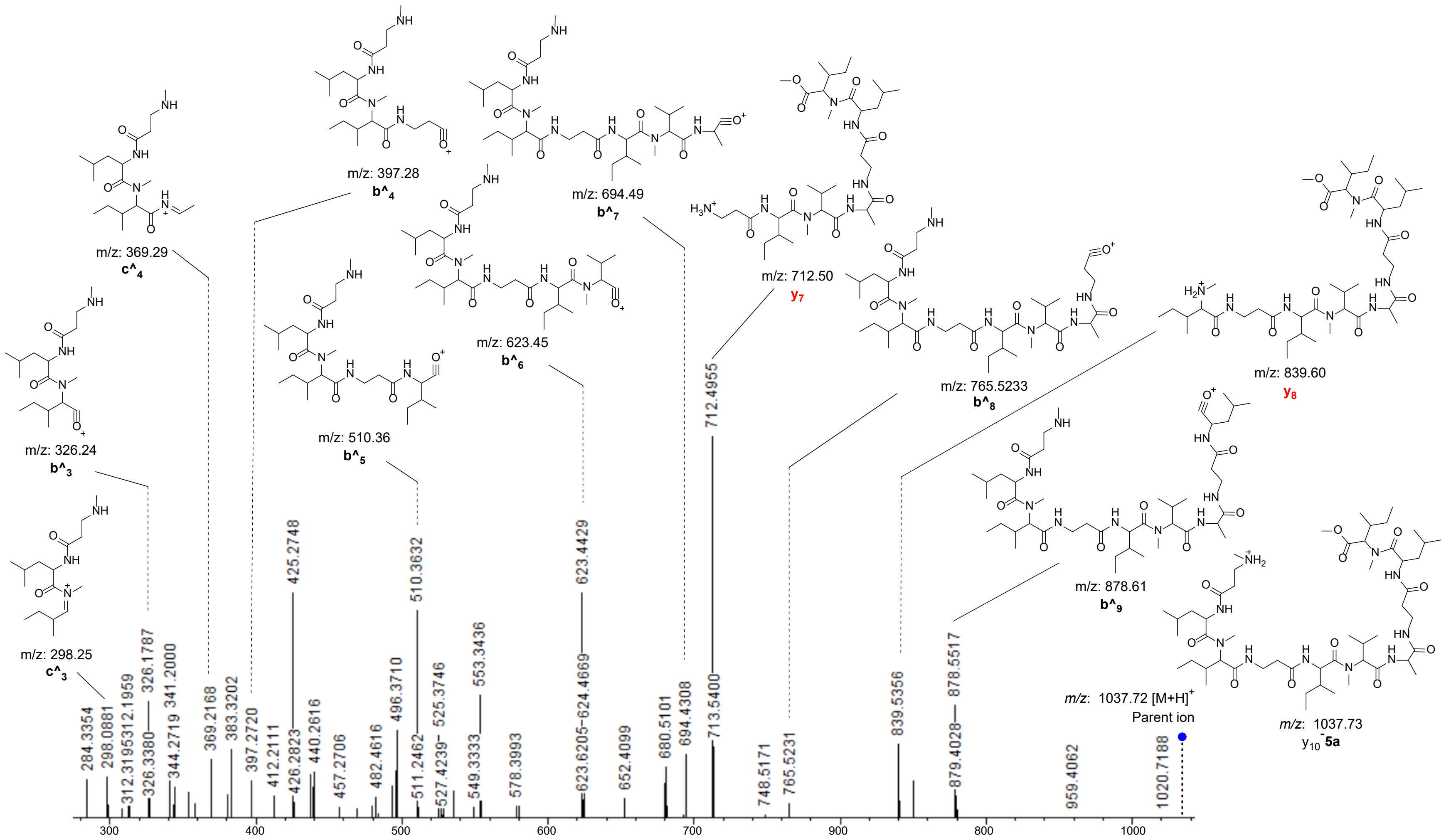

Figure S18. MS/MS/MS spectrum of fragment ion y_{10} of **5a**, *m/z* 1037.72 $[M+H]^+$, (red colored letter denoted fragment ions generated toward C-terminus, black colored letter denoted fragment ions toward N-terminus). The other detected fragment ions are listed in Table S10.

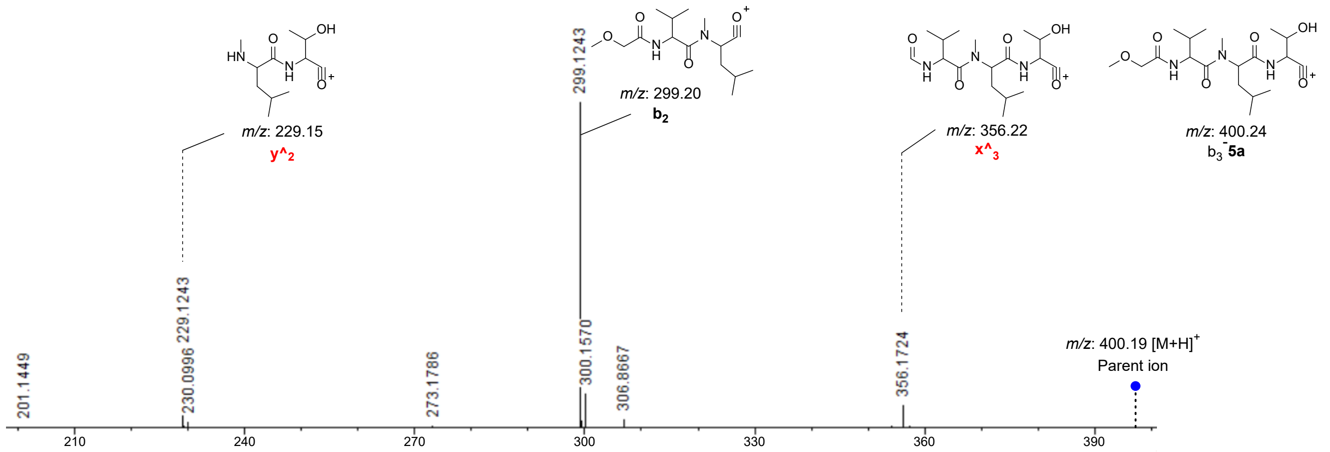

Figure S19. MS/MS/MS spectrum of fragment ion b3 of **5a**, *m/z* 400.19 $[M+H]^+$, (red colored letter denoted fragment ions generated toward C-terminus, black colored letter denoted fragment ions toward N-terminus).

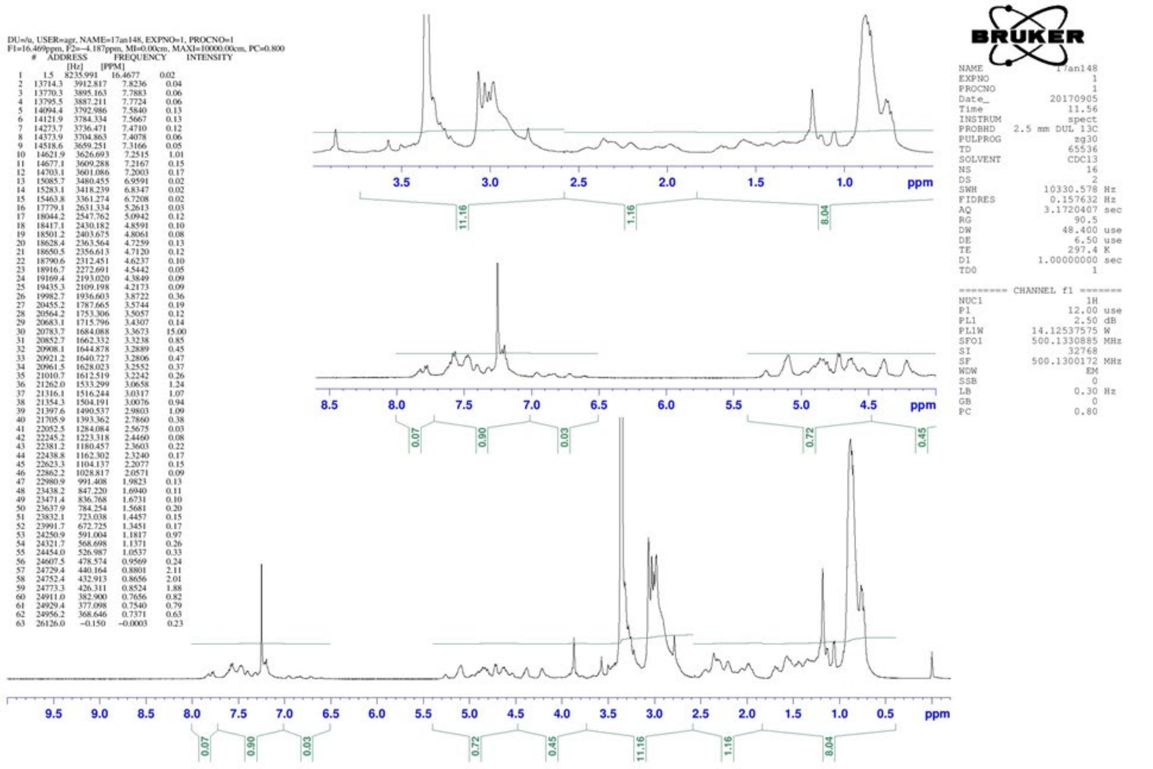

Figure S20. ^{1}H NMR spectrum ($CDCl_3$, 500 MHz) of compound **1**.

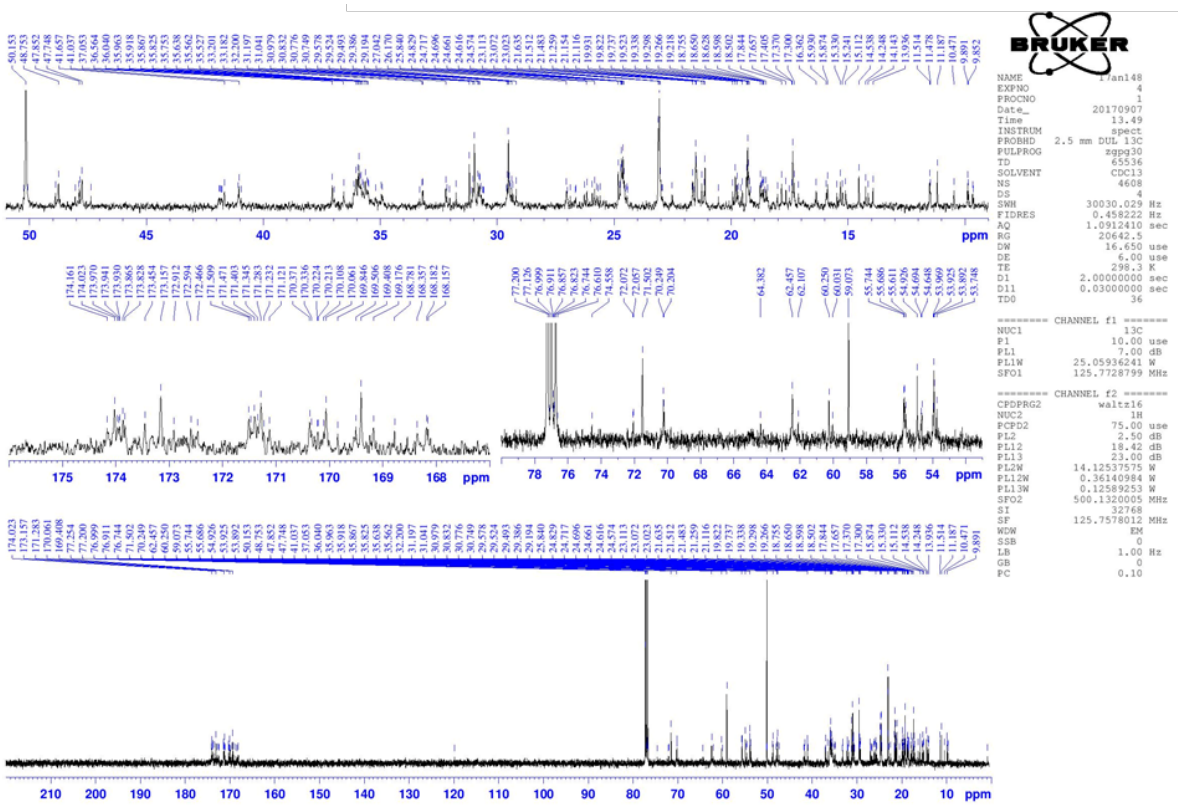

Figure S21. ^{13}C NMR spectrum ($CDCl_3$, 125 MHz) of compound **1**.

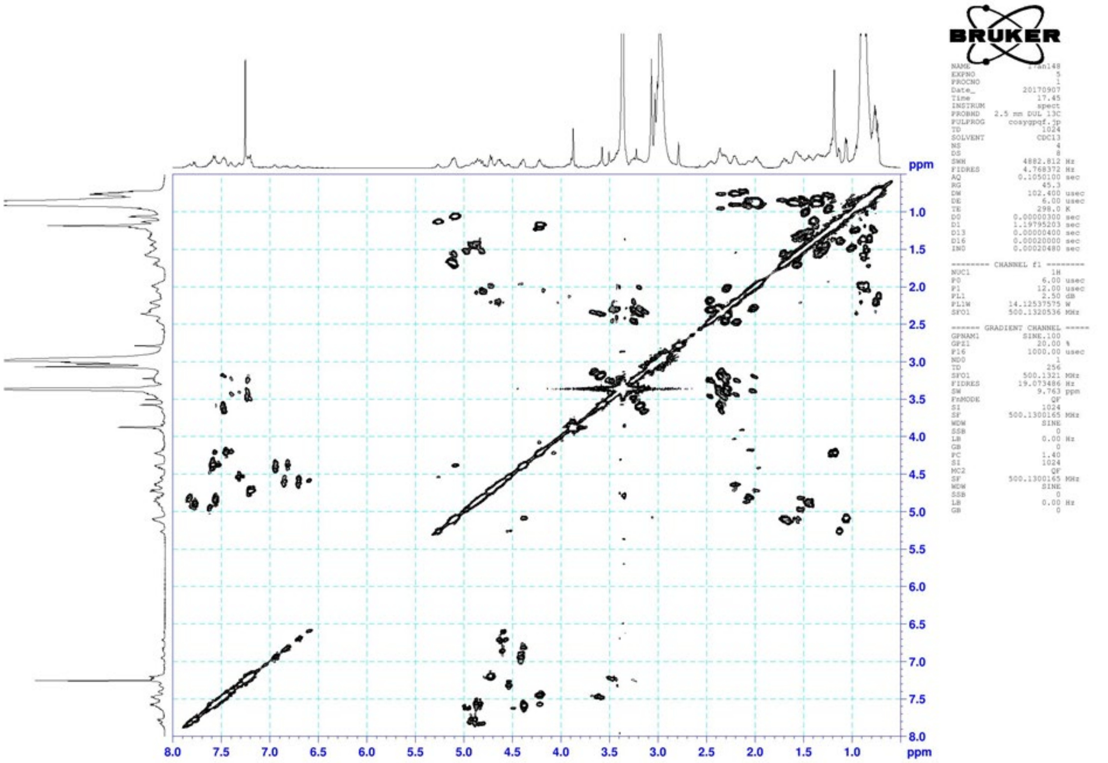

Figure S22. COSY NMR spectrum ($CDCl_3$, 500 MHz) of compound **1**.

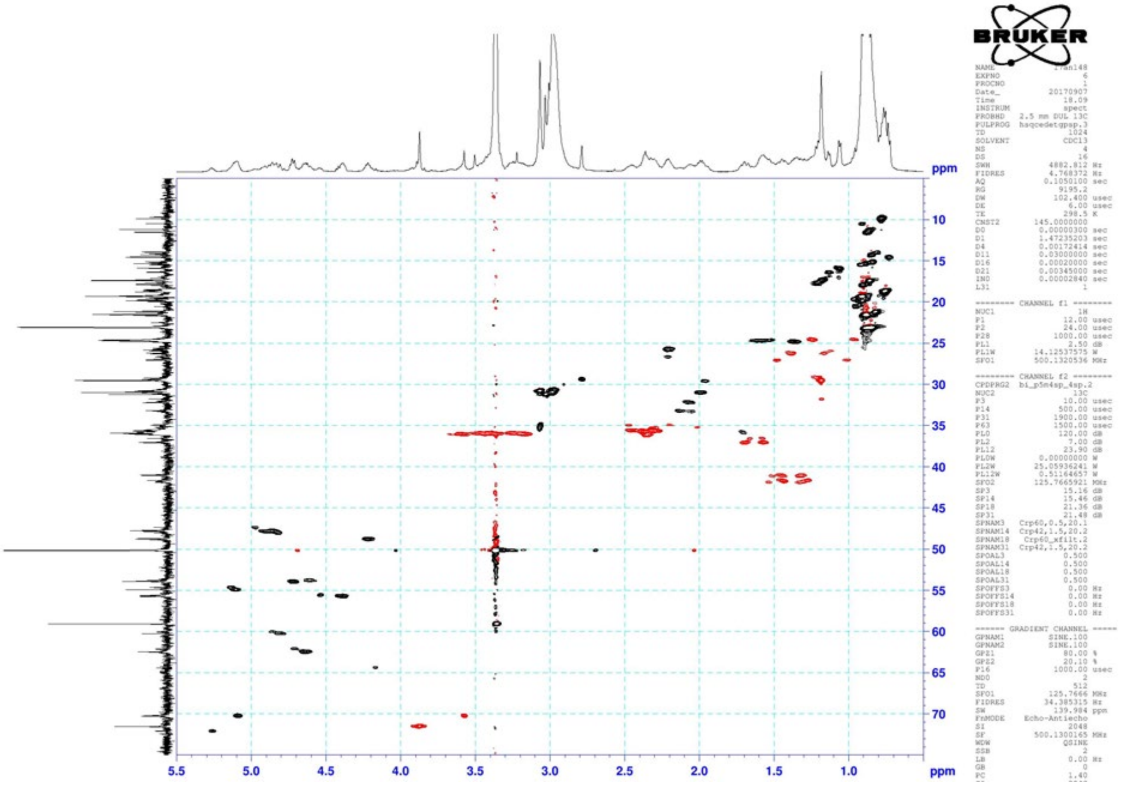

Figure S23. HSQC NMR spectrum ($CDCl_3$, 500 MHz) of compound **1**.

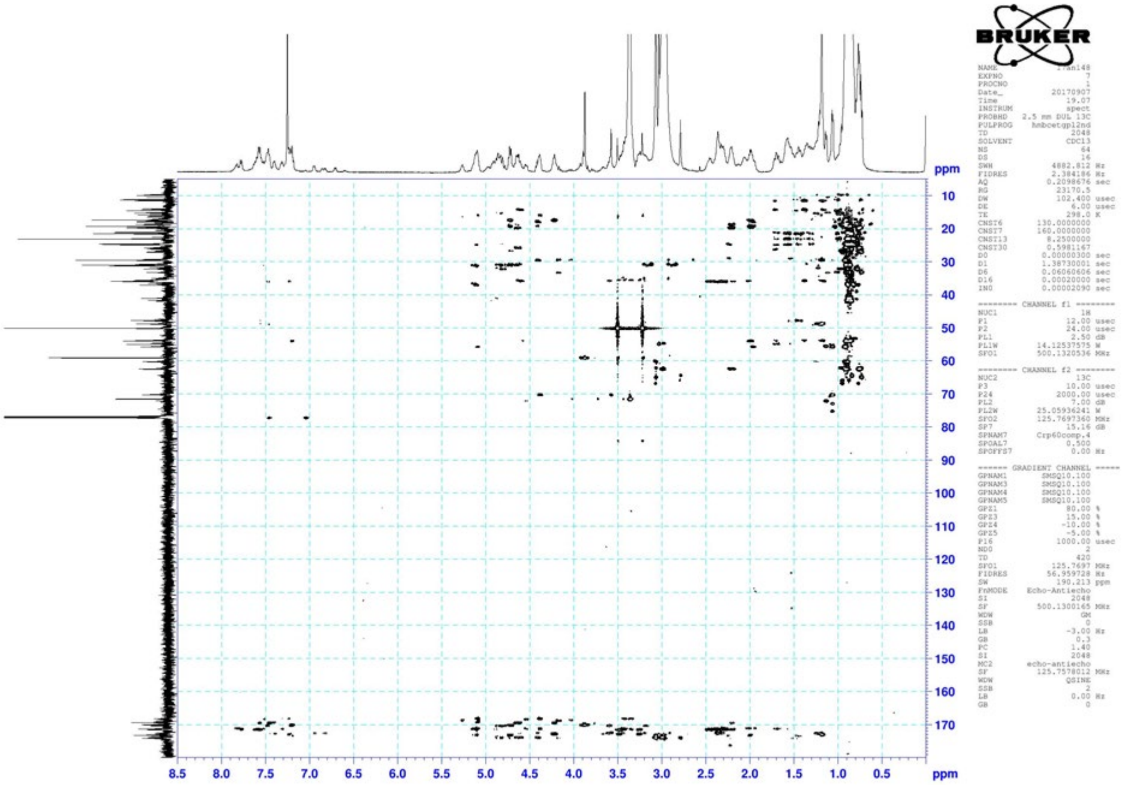

Figure S24. HMBC NMR spectrum ($CDCl_3$, 500 MHz) of compound **1**.

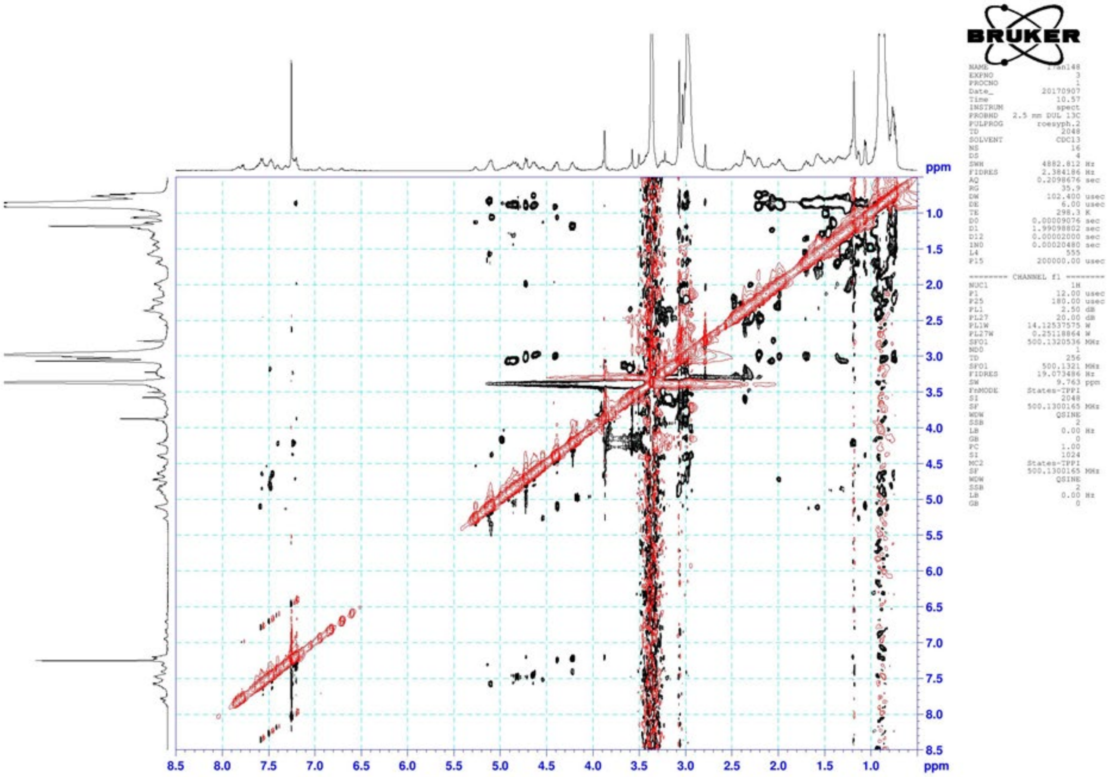

Figure S25. ROESY NMR spectrum ($CDCl_3$, 500 MHz) of compound **1**.

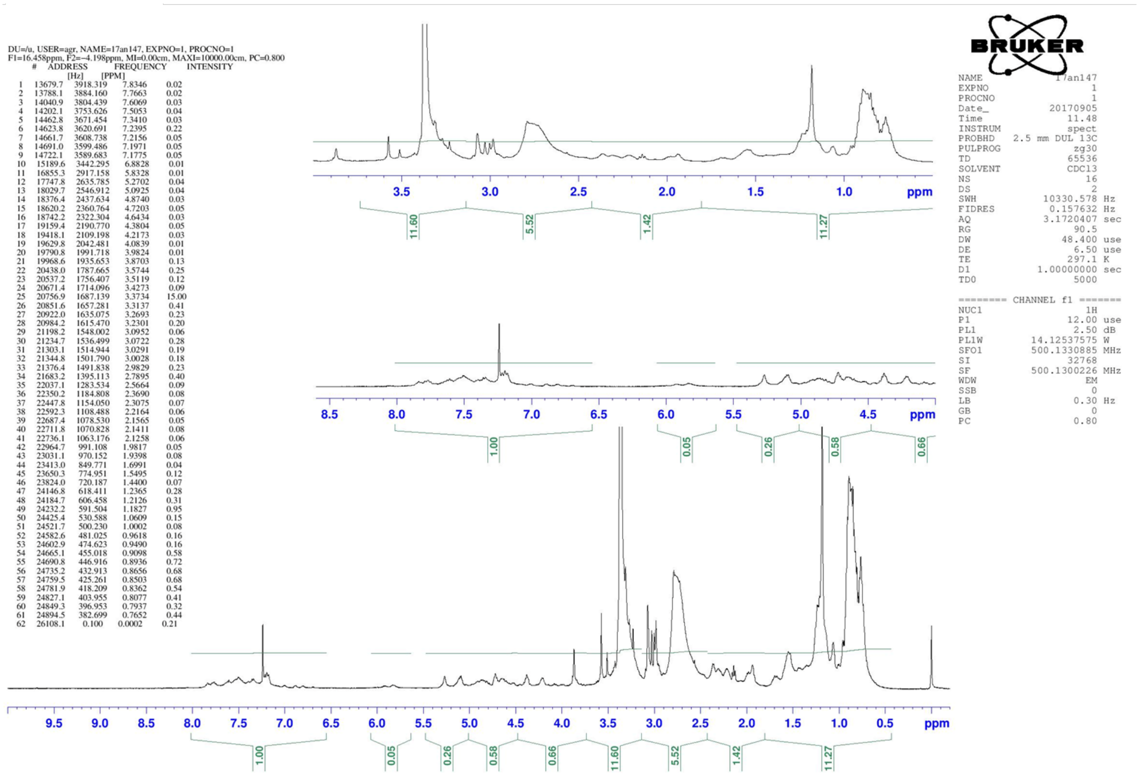

Figure S26. ^{1}H NMR spectrum ($CDCl_3$, 500 MHz) of compound **2**.

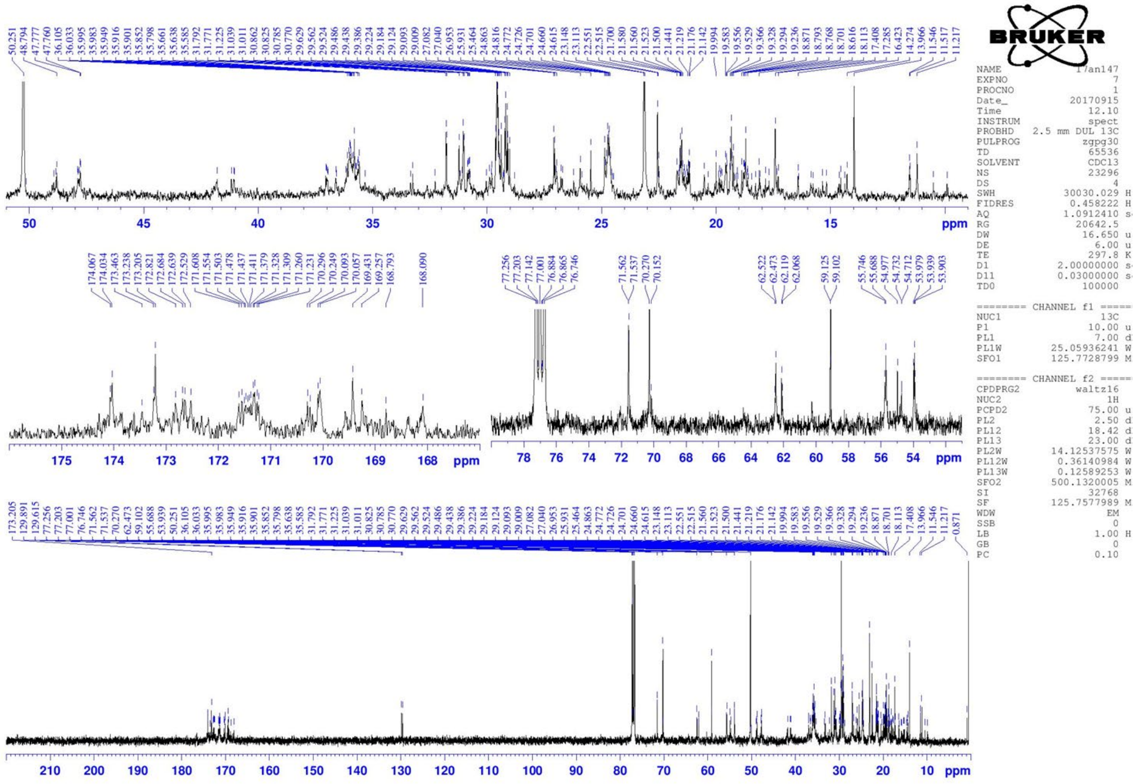

Figure S27. ^{13}C NMR spectrum ($CDCl_3$, 125 MHz) of compound **2**.

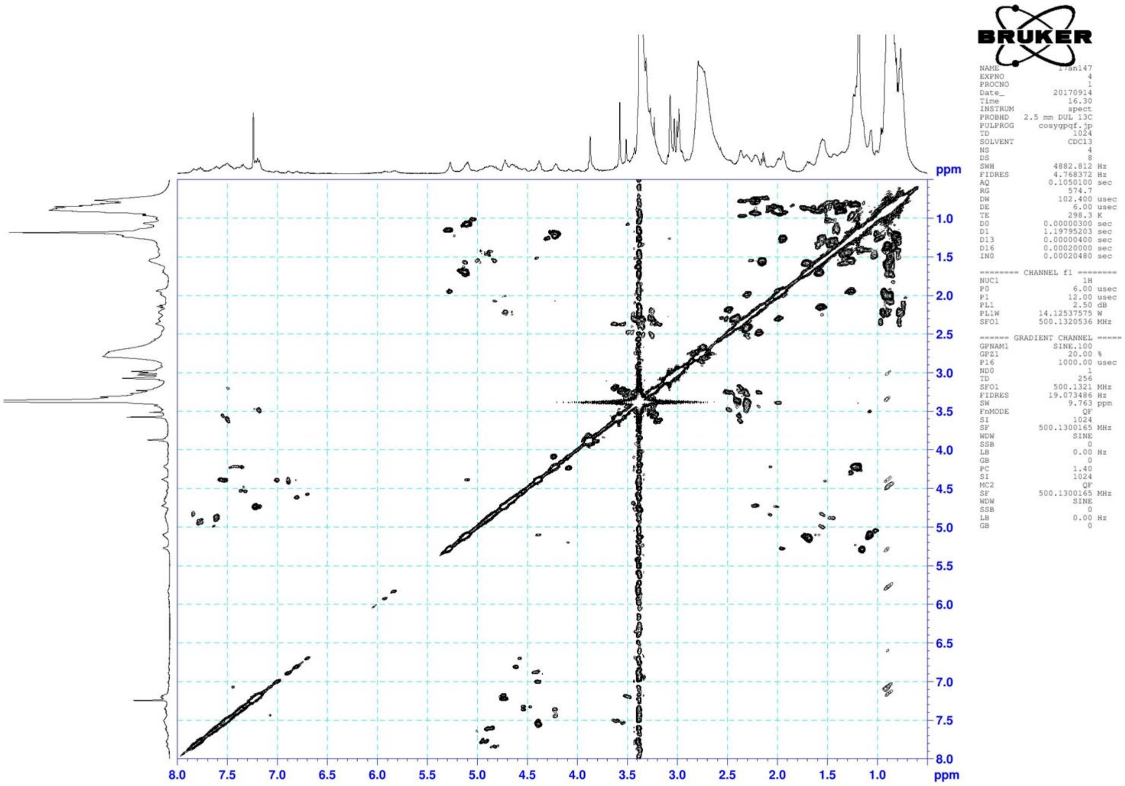

Figure S28. COSY NMR spectrum ($CDCl_3$, 500 MHz) of compound **2**.

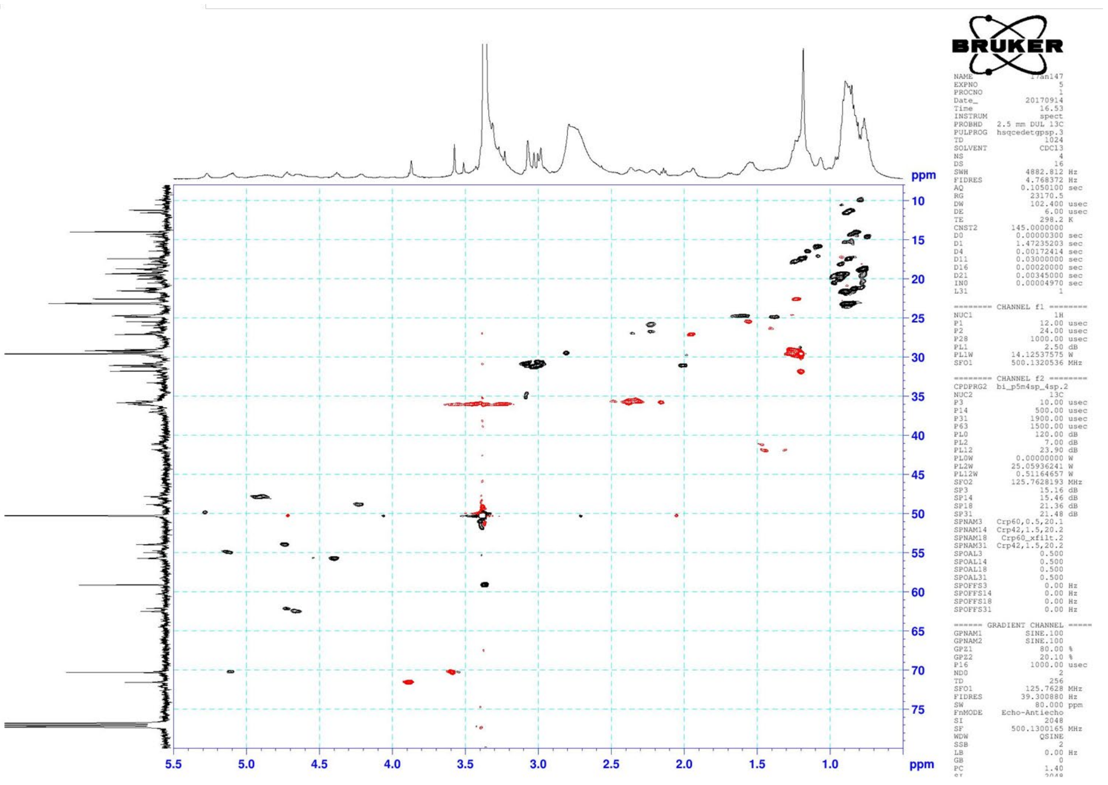

Figure S29. HSQC NMR spectrum ($CDCl_3$, 500 MHz) of compound **2**.

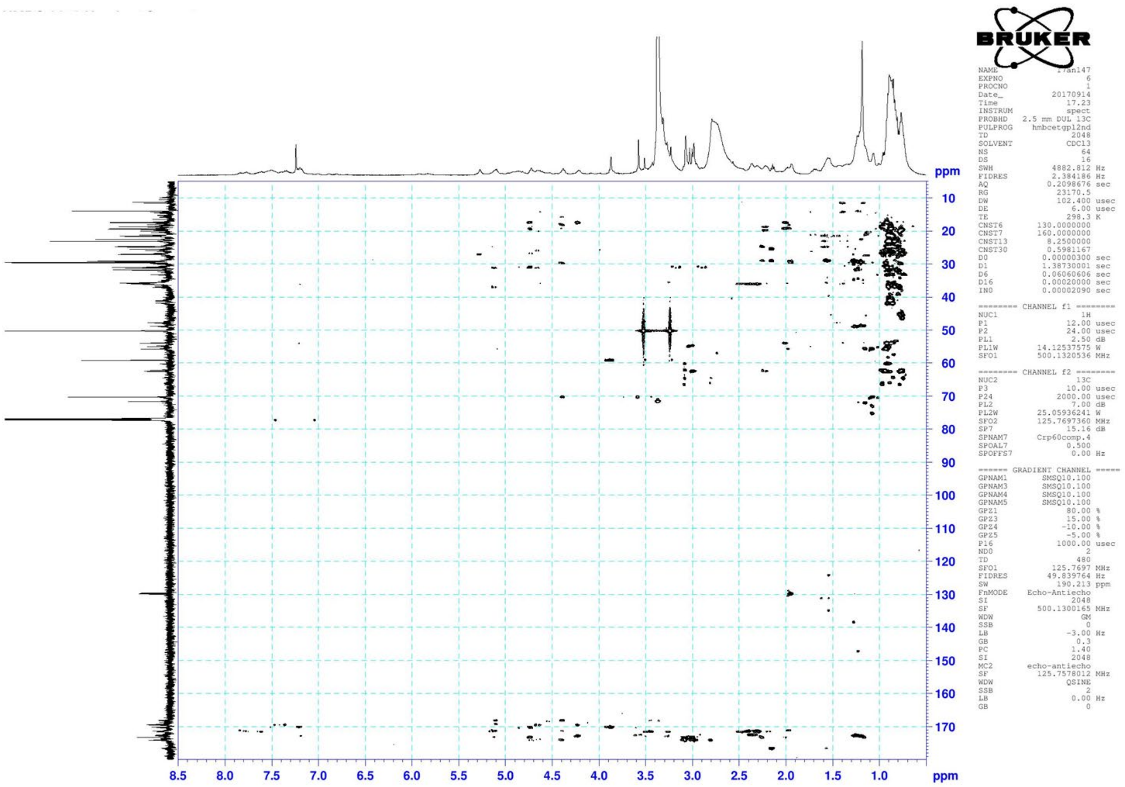

Figure S30. HMBC NMR spectrum ($CDCl_3$, 500 MHz) of compound **2**.

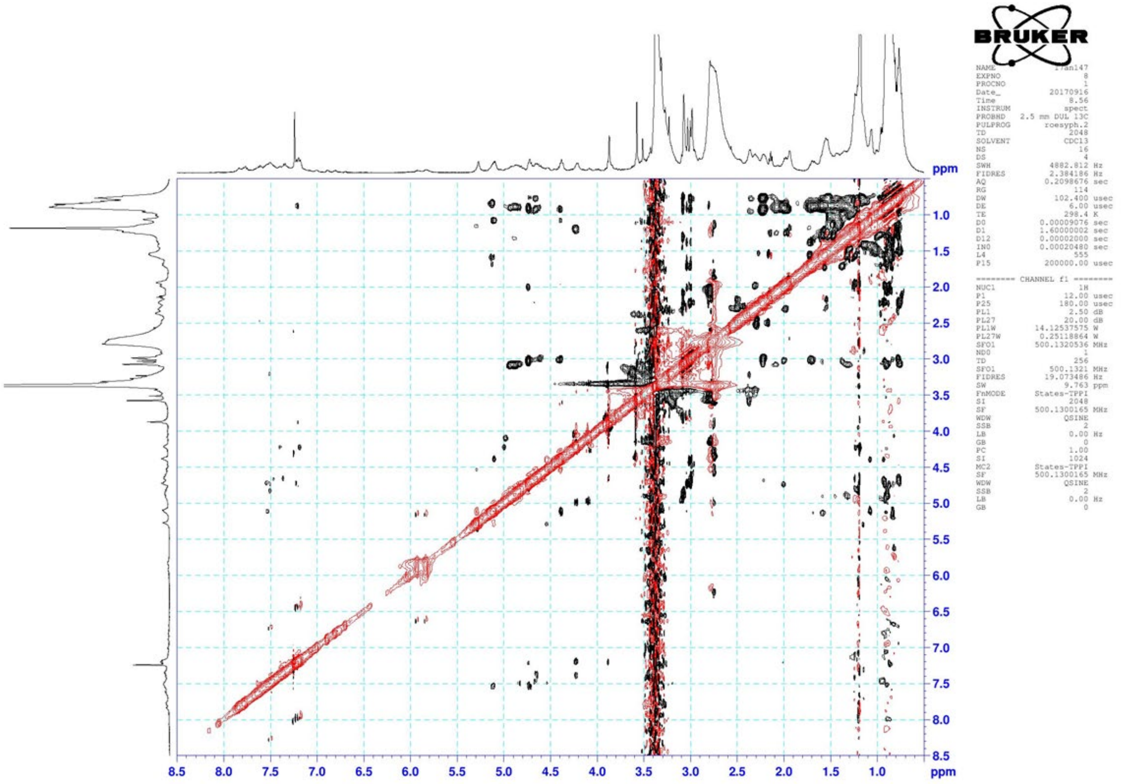

Figure S31. ROESY NMR spectrum ($CDCl_3$, 500 MHz) of compound **2**.

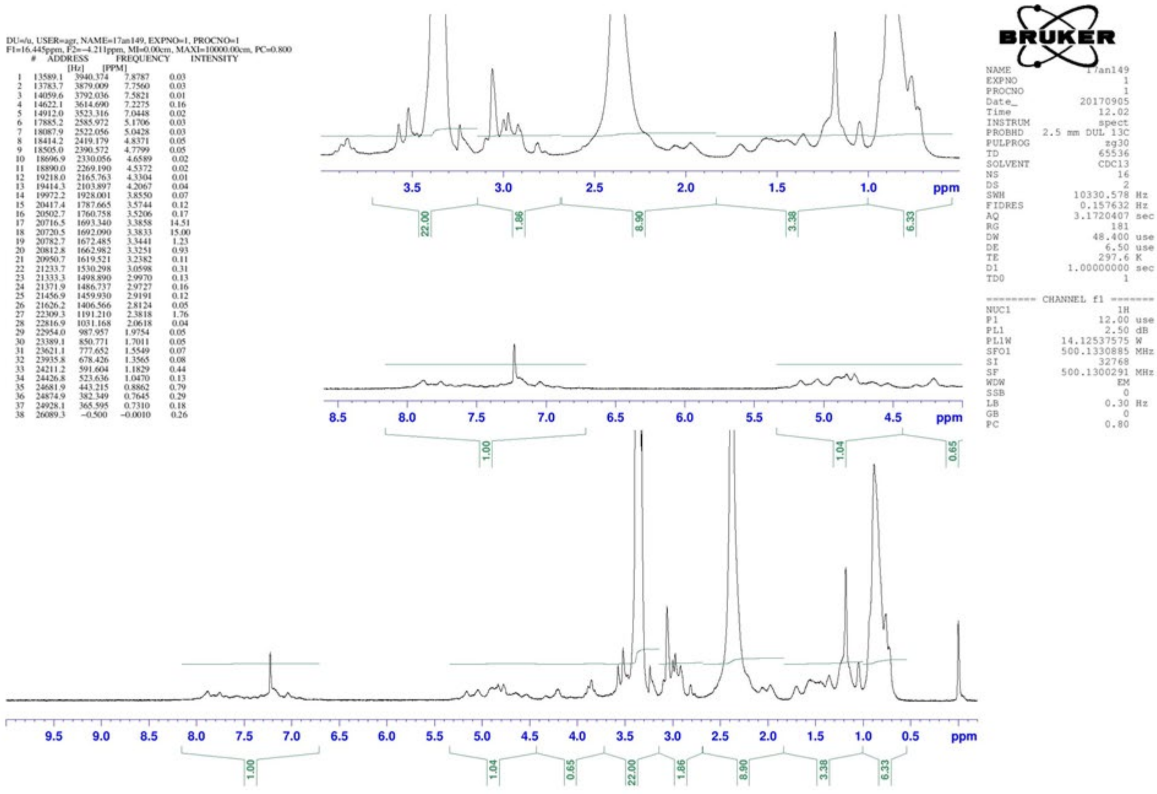

Figure S32. ^{1}H NMR spectrum ($CDCl_3$, 500 MHz) of compound **3**.

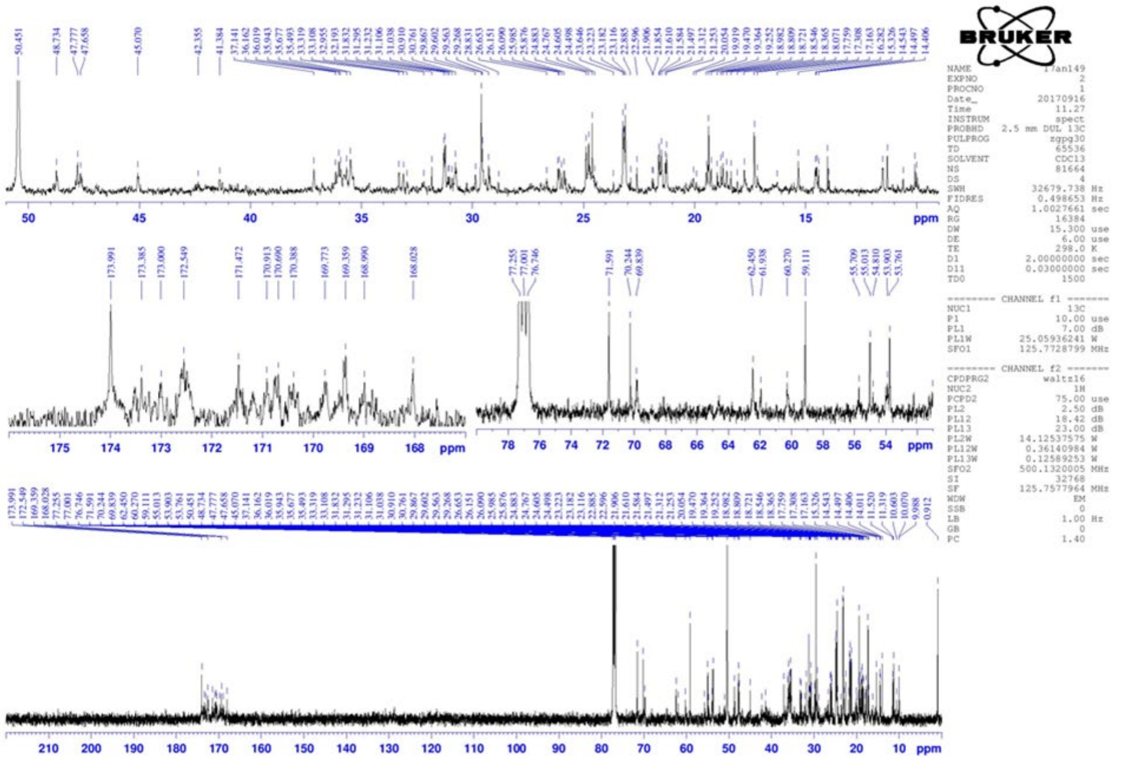

Figure S33. ^{13}C NMR spectrum ($CDCl_3$, 125 MHz) of compound **3**.

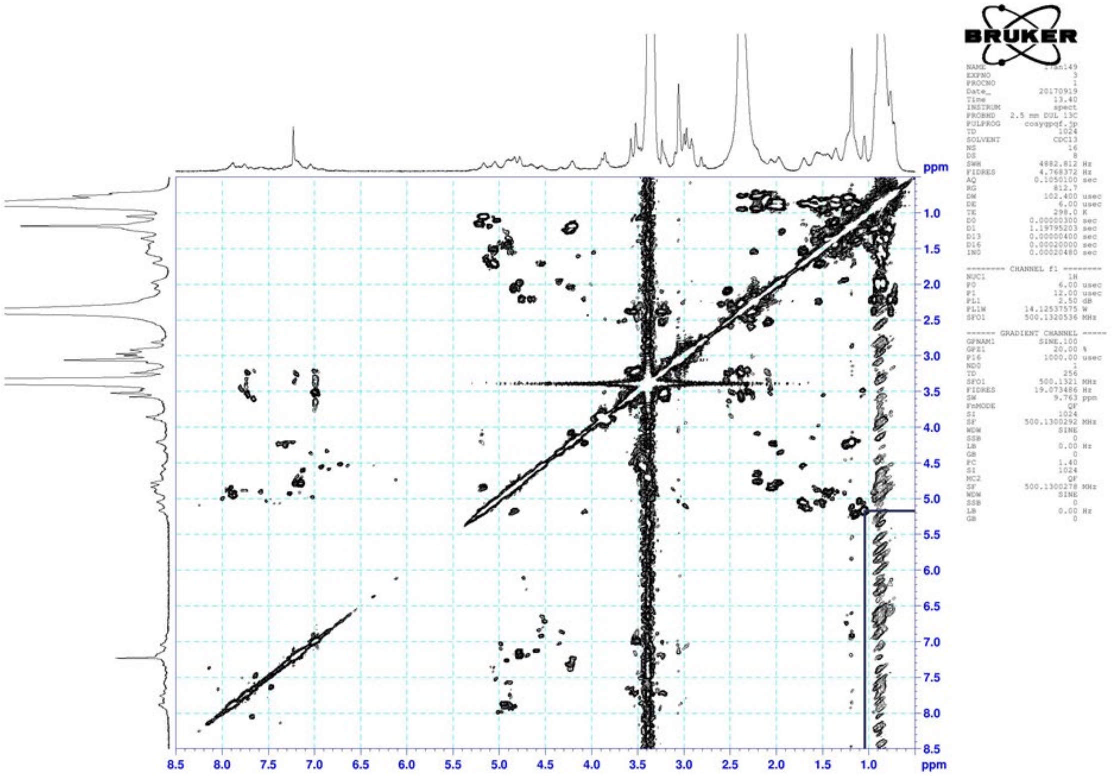

Figure S34. COSY NMR spectrum ($CDCl_3$, 500 MHz) of compound **3**.

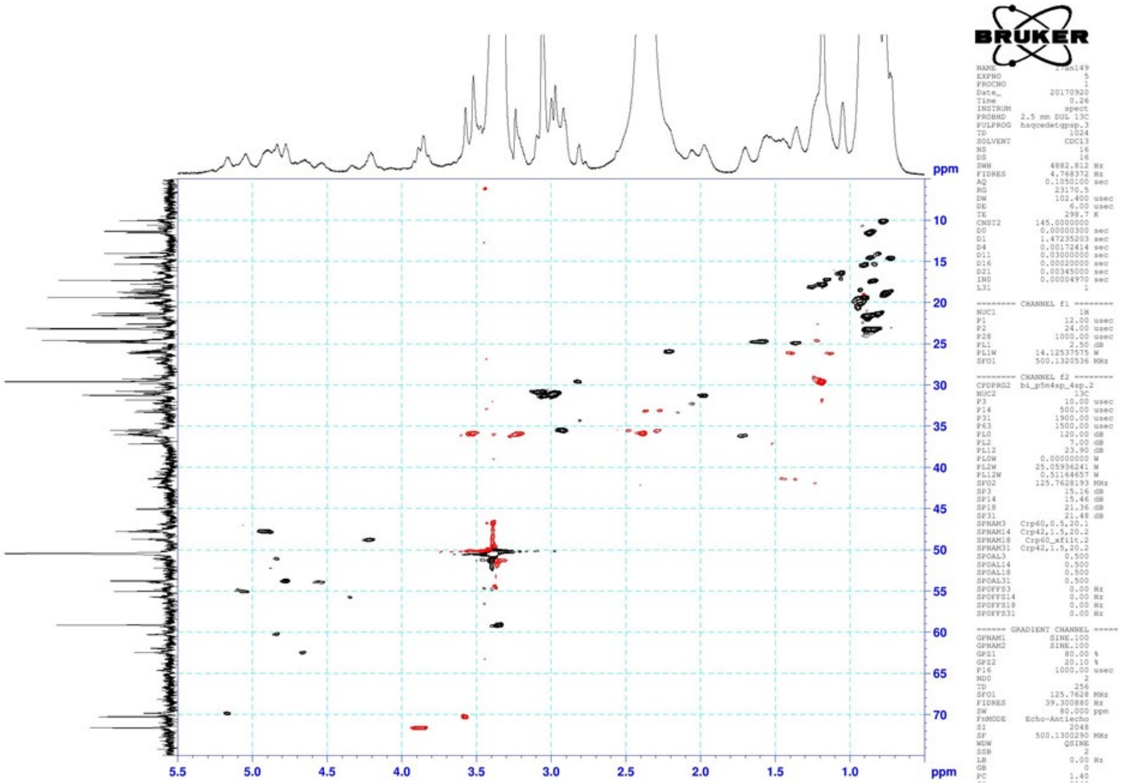

Figure S35. HSQC NMR spectrum ($CDCl_3$, 500 MHz) of compound **3**.

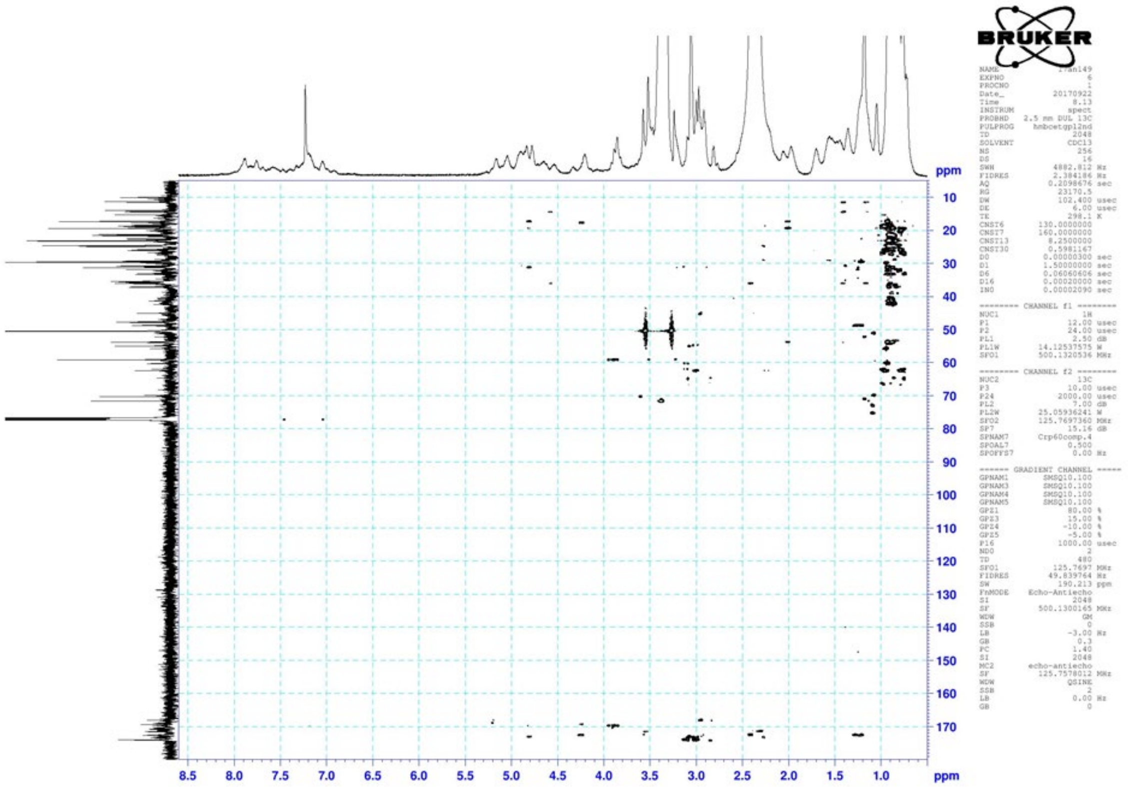

Figure S36. HMBC NMR spectrum ($CDCl_3$, 500 MHz) of compound **3**.

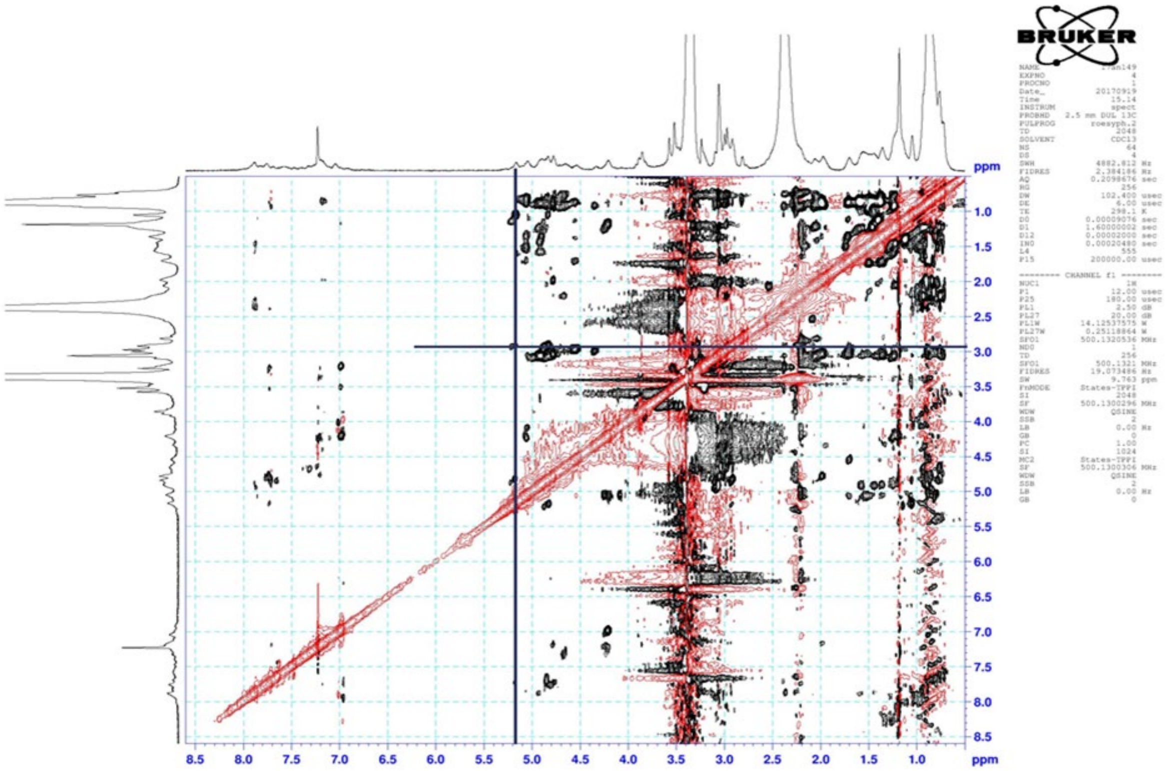

Figure S37. ROESY NMR spectrum ($CDCl_3$, 500 MHz) of compound **3**.

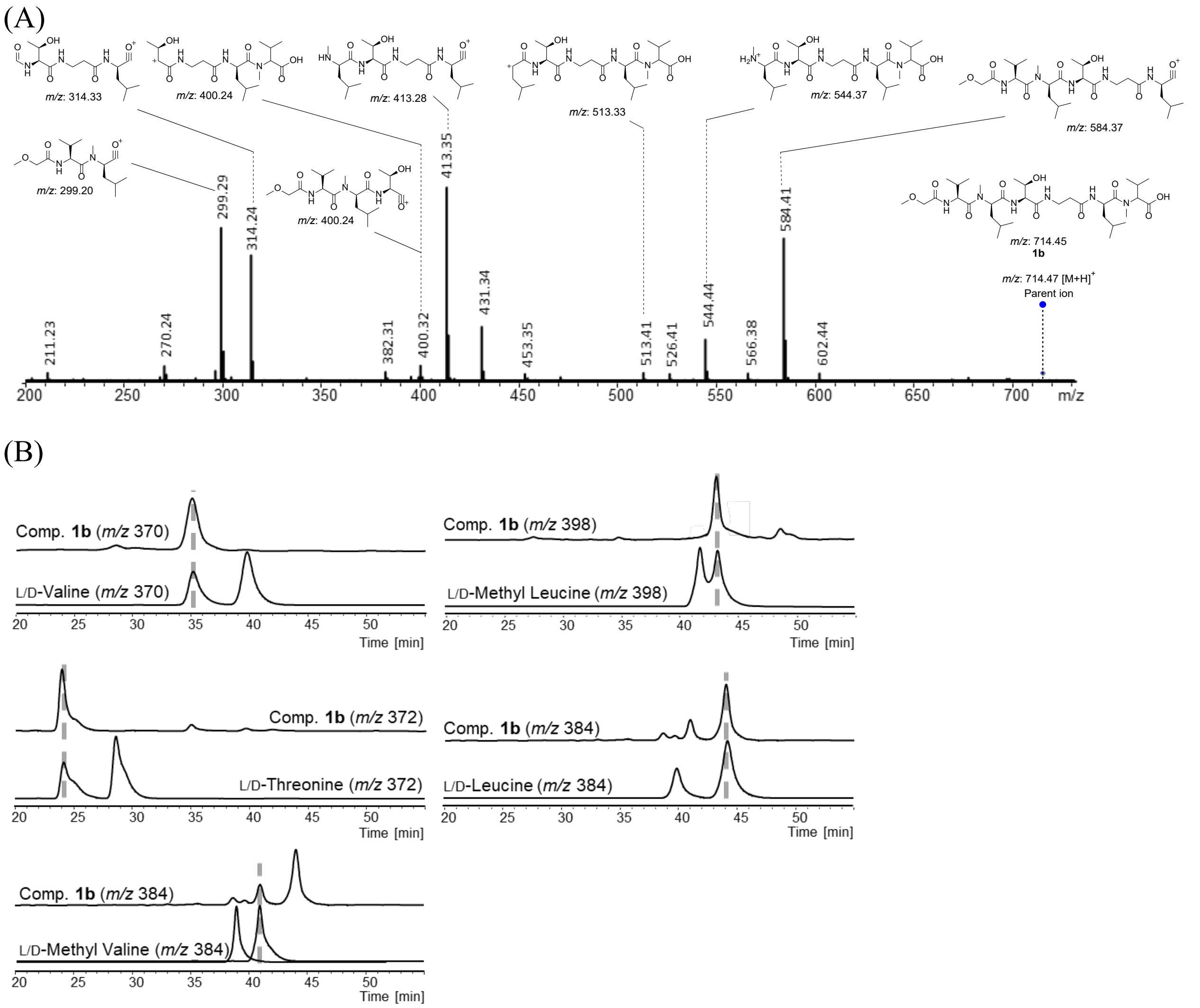

Figure S38. Tandem MS analysis of the targeted fragment **1b** derived from the N-terminal part of compound **1** (A), followed with Marfey's analysis to determine its absolute configuration (B).

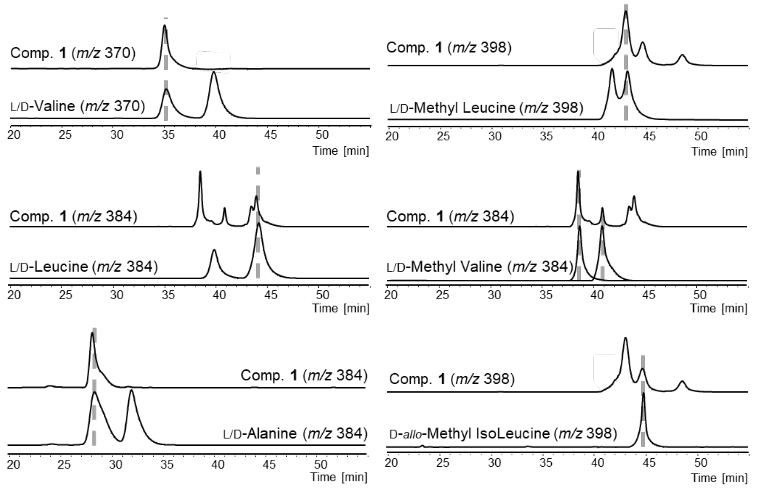

Figure S39. Marfey's analysis of the total hydrolysate of compound **1** in comparison with the amino acid standards.

(A)

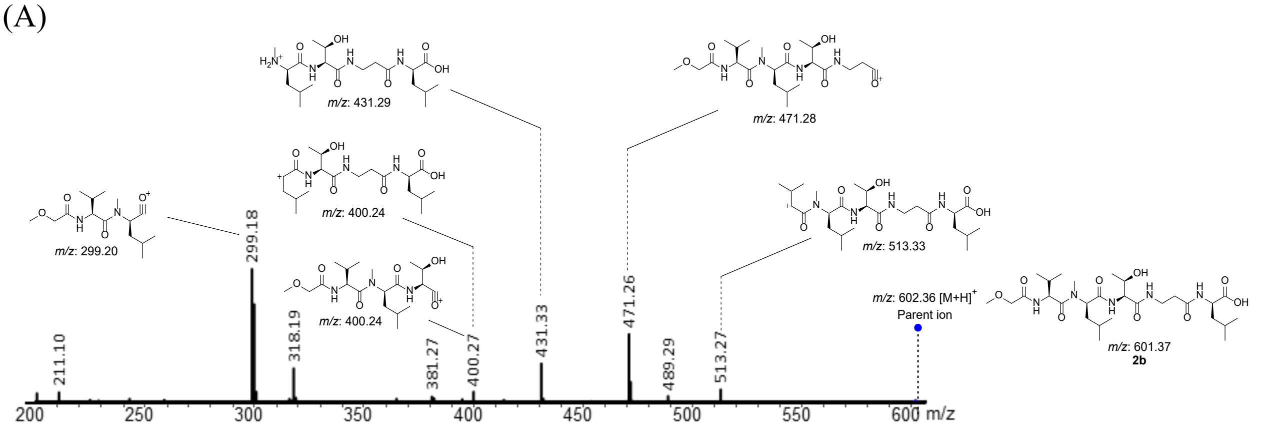

(B)

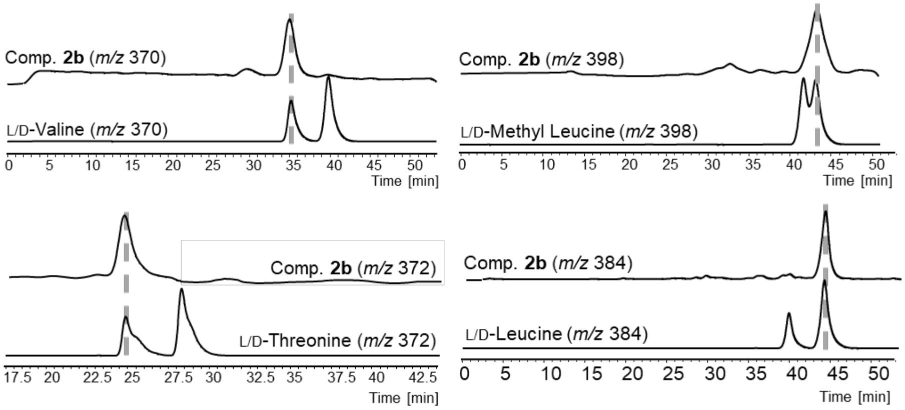

Figure S40. Tandem MS analysis of the targeted fragment **2b** derived from the N-terminal part of compound **2** (A), followed with Marfey's analysis to determine its absolute configuration (B).

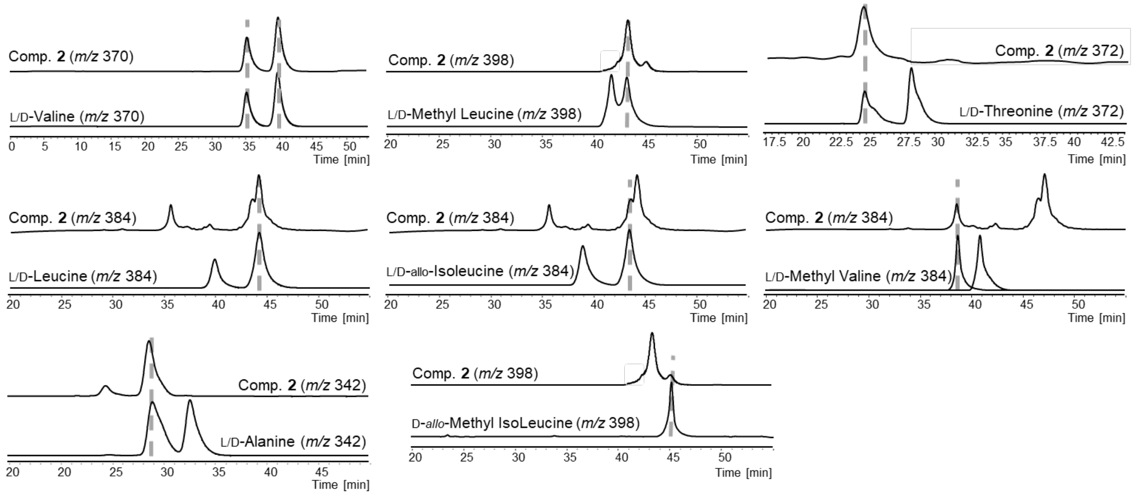

Figure S41. Marfey's analysis of the total hydrolysate of compound **2** in comparison with the amino acid standards.

(A)

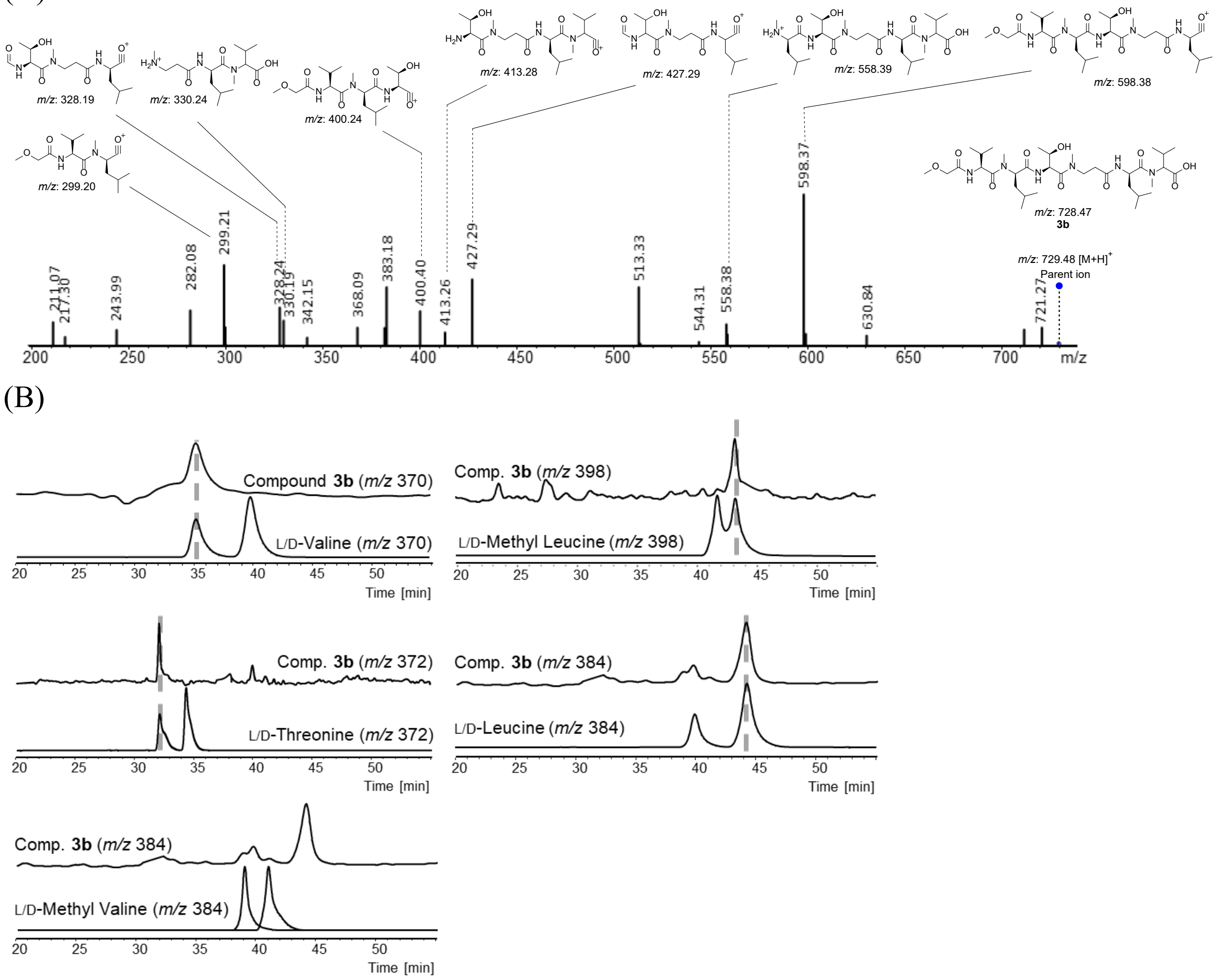

Figure S42. Tandem MS analysis of the targeted fragment **3b** derived from the N-terminal part of compound **3** (A), followed with Marfey's analysis to determine its absolute configuration (B).

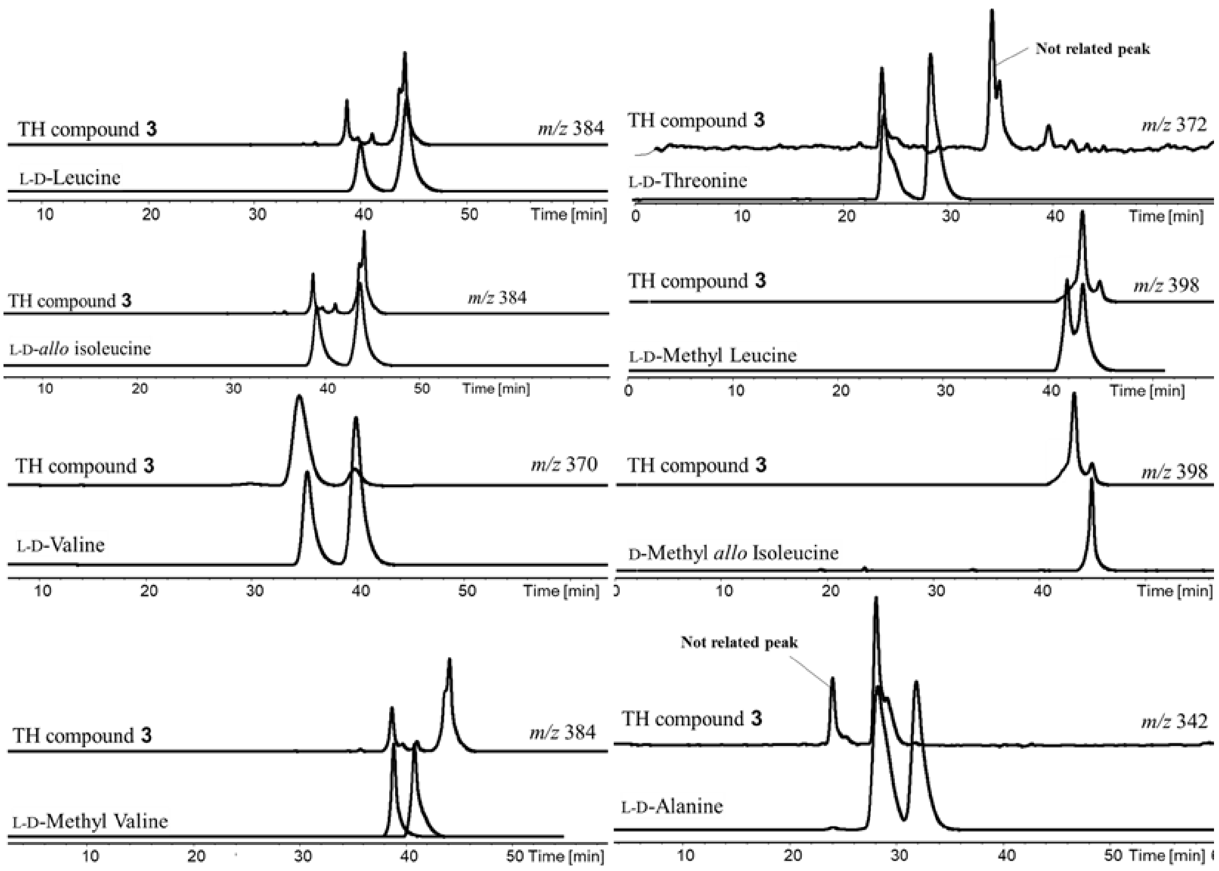

Figure S43. Marfey's analysis of the total hydrolysate of compound **3** in comparison with the amino acid standards.

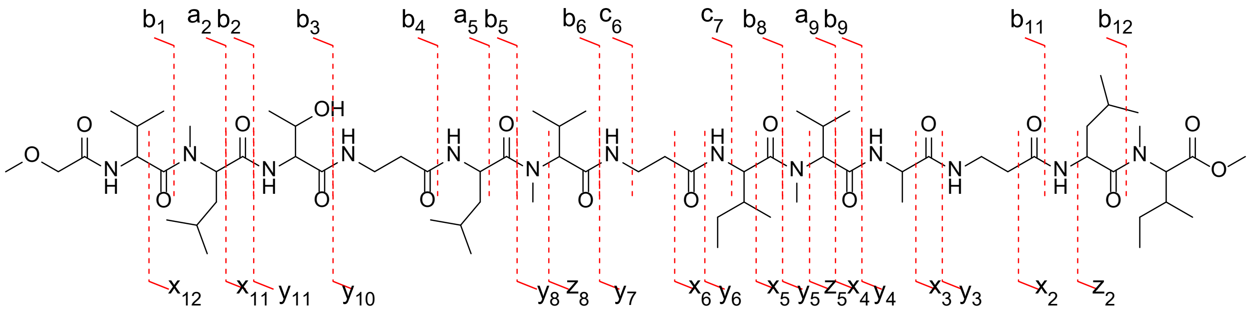

1a

Table S1. List of obtained fragment ions from MS/MS analysis of **1a**.

Ion	Calc. (Da)	Obs. (Da)	Δdelta (Da)	Ion	Calc. (Da)	Obs. (Da)	Δdelta (Da)
b_1	172.0968	172.0716	0.0253	z_2	256.1907	256.1165	0.0742
a_2	271.2016	271.1479	0.0537	x_2+H_2O	317.1965	317.2708	0.0742
b_2+H_2O	317.1965	317.2708	0.0742	x_2	299.1965	299.1774	0.0192
b_2	299.1965	299.1774	0.0192	y_3	344.2544	344.3039	0.0495
b_3	400.2442	400.0228	0.2214	x_3	370.2337	370.2658	0.0322
b_4	471.2813	471.2503	0.0310	z_4+H_2O	416.2650	416.3188	0.0539
a_5	556.3705	556.3348	0.0356	y_4	415.2915	415.2365	0.0550
b_5	584.3654	584.3572	0.0082	x_4+H_2O	459.2708	459.2392	0.0316
b_6	697.4495	697.0222	0.4273	x_4	441.2708	441.2595	0.0112
c_6	714.4760	714.6461	0.1701	z_5+H_2O	515.3334	515.9225	0.5891
b_7+H_2O	786.4866	786.0298	0.4568	z_5	497.3334	497.3955	0.0622
c_7	785.5131	785.9608	0.4477	y_5	528.3756	528.4362	0.0607
b_8	881.5706	881.6166	0.0460	x_5+H_2O	572.3548	572.3600	0.0052
a_9	966.6598	966.5709	0.0889	x_5	554.3548	554.5071	0.1523
b_9	994.6547	994.7037	0.0490	z_6+H_2O	642.4331	642.0222	0.4109
b_{11}	1136.7289	1136.7332	0.0042	y_6	641.4596	641.3369	0.1227
b_{12}	1249.8130	1249.8666	0.0536	x_6	667.4389	667.4990	0.0601
				z_7+H_2O	713.4702	713.6125	0.1423
				y_7	712.4967	712.4990	0.0023
				z_8	794.5386	794.5554	0.0168
				y_8	825.5805	825.6157	0.0352
				z_9+H_2O	939.6383	939.3075	0.3308
				$z_{10}+H_2O$	1010.6754	1010.5914	0.0841
				y_{10}	1009.7020	1009.6266	0.0754
				$z_{11}+H_2O$	1111.7231	1111.6885	0.0346
				y_{11}	1110.7497	1110.7552	0.0056
				x_{11}	1136.7289	1136.7332	0.0042
				x_{12}	1263.8286	1263.8693	0.0406

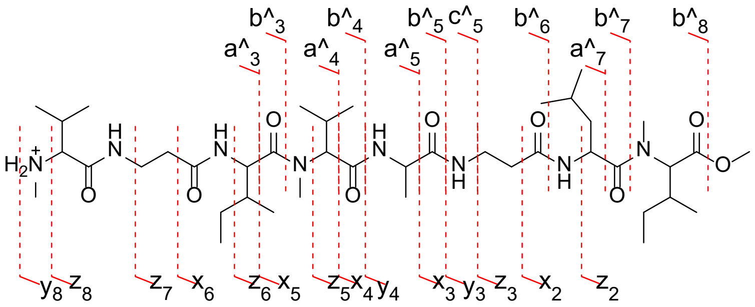

y_8-**1a**

Table S2. List of obtained fragment ions from MS/MS/MS analysis of y_8-**1a**.

Ion	Calc. (Da)	Obs. (Da)	Δdelta (Da)	Ion	Calc. (Da)	Obs. (Da)	Δdelta (Da)
a^$_3$	270.2176	270.1913	0.0263	z_2	256.1907	256.0798	0.1109
b^$_3$	298.2125	298.1420	0.0705	x_2	299.1965	299.1702	0.0263
a^$_4$	383.3017	383.3098	0.0081	z_3	327.2278	327.2412	0.0134
b^$_4$	411.2966	411.2961	0.0005	y_3	344.2544	344.2126	0.0418
a^$_5$	454.3388	454.3857	0.0469	x_3	370.2337	370.2788	0.0452
b^$_5$	482.3337	482.3195	0.0142	y_4	415.2915	415.2700	0.0215
c^$_5$	499.3603	499.3214	0.0388	x_4	441.2708	441.3353	0.0645
b^$_6$	553.3708	553.3790	0.0082	z_5+H_2O	515.3334	515.2952	0.0381
a^$_7$	638.4600	638.5156	0.0556	z_5	497.3334	497.3034	0.0300
b^$_7$	666.4549	666.4556	0.0007	y_5	528.3756	528.4121	0.0365
b^$_8$	793.5546	793.5571	0.0025	x_5	554.3548	554.3976	0.0428
				z_6	624.4331	624.4590	0.0260
				x_6	667.4389	667.4561	0.0172
				z_8	794.5386	794.6066	0.0680

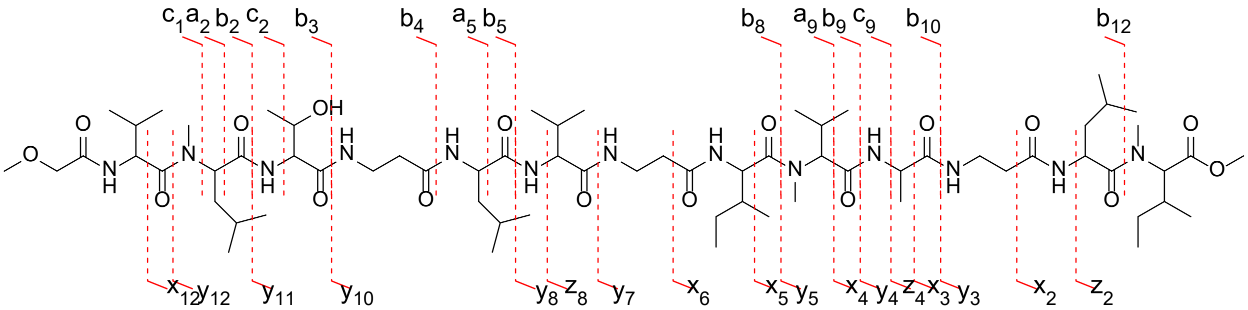

2a

Table S3. List of obtained fragment ions from MS/MS analysis of **2a**.

Ion	Calc. (Da)	Obs. (Da)	Δdelta (Da)	Ion	Calc. (Da)	Obs. (Da)	Δdelta (Da)
c_1	203.1390	203.0924	0.0467	z_2	256.1907	256.1545	0.0362
a_2	271.2016	271.1439	0.0578	x_2	299.1965	299.2136	0.0171
b_2	299.1965	299.2136	0.0171	y_3	344.2544	344.3035	0.0491
c_2	316.2231	316.2105	0.0125	x_3	370.2337	370.2604	0.0268
b_3	400.2442	400.2528	0.0086	z_4	398.2650	398.2693	0.0044
b_4	471.2813	471.3384	0.0570	y_4	415.2915	415.3503	0.0588
a_5	556.3705	556.3973	0.0268	x_4	441.2708	441.3018	0.0310
b_5	584.3654	584.3900	0.0246	y_5	528.3756	528.4907	0.1151
$b_5 + H_2O$	602.3654	602.4204	0.0550	x_5	554.3548	554.3870	0.0322
$a_6 + H_2O$	673.4389	673.5240	0.0851	x_6	667.4389	667.5293	0.0904
b_8	867.5550	867.7025	0.1475	y_7	712.4967	712.5329	0.0362
a_9	952.6441	952.8411	0.1969	z_8	794.5386	794.6181	0.0795
b_9	980.6391	980.8881	0.2491	y_8	811.5652	811.6312	0.0660
c_9	997.6656	997.3965	0.2690	x_8+H_2O	855.5444	855.4921	0.0523
b_{10}	1051.6762	1051.7500	0.0738	$z_{10}+H_2O$	996.6598	996.7625	0.1027
$a_{11}+H_2O$	1112.7184	1112.7456	0.0272	y_{10}	995.6863	995.7158	0.0295
b_{12}	1235.7973	1235.7866	0.0107	$z_{11}+H_2O$	1097.7075	1097.8020	0.0945
				y_{11}	1096.7340	1096.8569	0.1229
				y_{12}	1223.8337	1223.7208	0.1129
				x_{12}	1249.8130	1249.8978	0.0848

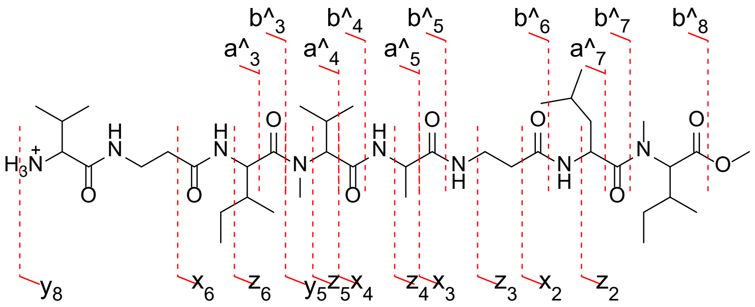

y8-**2a**

Table S4. List of obtained fragment ions from MS/MS/MS analysis of y8-**2a**.

Ion	Calc. (Da)	Obs. (Da)	Δdelta (Da)	Ion	Calc. (Da)	Obs. (Da)	Δdelta (Da)
a^3	256.2020	256.2199	0.0180	z_2	256.1907	256.2199	0.0292
b^3	284.1969	284.1956	0.0013	x_2	299.1965	299.1811	0.0155
a^4	369.2860	369.2751	0.0109	z_3	327.2278	327.2722	0.0444
b^4	397.2809	397.2563	0.0247	x_3	370.2337	370.2709	0.0373
a^5	440.3231	440.2814	0.0418	z_4	398.2650	398.3205	0.0556
b^5	468.3181	468.3733	0.0553	x_4	441.2708	441.3669	0.0962
b^6	539.3552	539.2705	0.0847	z_5	497.3336	497.3200	0.0136
a^7	624.4443	624.3962	0.0481	y_5	528.3756	528.3950	0.0195
b^7	652.4392	652.4911	0.0519	z_6	624.4331	624.3962	0.0368
b^8	779.5389	779.5640	0.0250	x_6	667.4389	667.4293	0.0096

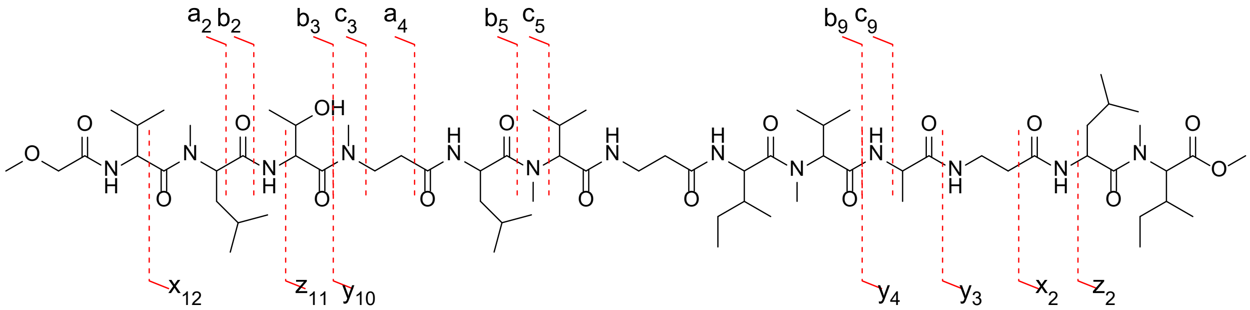

3a

Table S5. List of obtained fragment ions from MS/MS analysis of **3a**.

Ion	Calc. (Da)	Obs. (Da)	Δdelta (Da)	Ion	Calc. (Da)	Obs. (Da)	Δdelta (Da)
a_2	271.2016	271.1167	0.0850	z_2	256.1907	256.2096	0.0189
b_2	299.1965	299.1906	0.0060	x_2	299.1965	299.1906	0.0060
b_3	400.2442	400.2771	0.0329	y_3	344.2544	344.1664	0.0880
c_3+H_2O	449.3864	449.9526	0.5662	z_4+H_2O	416.2650	416.5485	0.2836
c_3	431.3864	431.1473	0.2391	y_4	415.2915	415.2530	0.0385
a_4	457.3021	457.1173	0.1848	y_8+H_2O	843.5808	843.9647	0.3839
b_5	598.3810	598.4725	0.0915	y_{10}	1023.7176	1023.7256	0.0080
c_5	629.4232	629.4316	0.0083	$z_{11}+H_2O$	1125.7388	1125.7288	0.0100
b_9	1008.6704	1008.6068	0.0635	y_{11}	1124.7653	1124.7495	0.0158
c_9	1025.6969	1025.7659	0.0690	x_{12}	1277.8443	1277.8545	0.0102

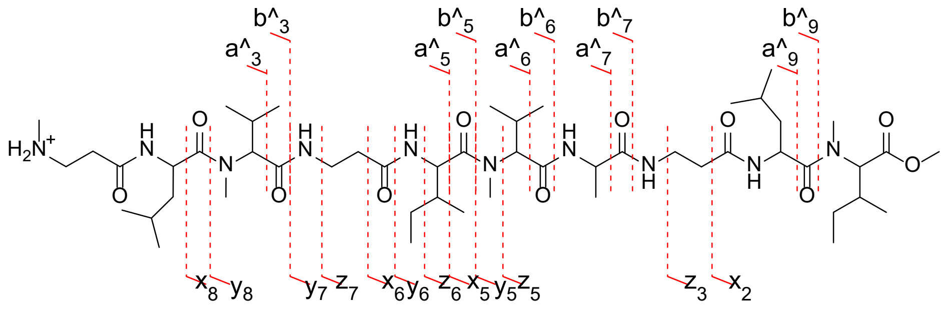

y10-**3a**

Table S6. List of obtained fragment ions from MS/MS/MS analysis of y10-**3a**.

Ion	Calc. (Da)	Obs. (Da)	Δdelta (Da)	Ion	Calc. (Da)	Obs. (Da)	Δdelta (Da)
a^$_3$	284.2333	284.1164	0.1169	x_2	299.1965	299.1617	0.0348
b^$_3$	312.2282	312.1706	0.0576	z_3	327.2278	327.1898	0.0381
a^$_4$+H_2O	373.2704	373.2407	0.0296	z_5	497.3334	497.2705	0.0629
a^$_5$	468.3544	468.1800	0.1744	y_5	528.3756	528.3364	0.0392
b^$_5$+H_2O	514.3494	514.2982	0.0511	x_5	554.3548	554.3978	0.0430
b^$_5$	496.3494	496.3857	0.0364	z_6	624.4331	624.4921	0.0590
a^$_6$	581.4385	581.4949	0.0564	y_6	641.4596	641.5109	0.0512
b^$_6$	609.4334	609.4700	0.0366	x_6	667.4389	667.4294	0.0094
a^$_7$	652.4756	652.5311	0.0555	z_7+H_2O	713.4702	713.3705	0.0996
b^$_7$+H_2O	698.4705	698.5139	0.0434	z_7	695.4702	695.4602	0.0100
b^$_7$	680.4705	680.5207	0.0502	y_7	712.4967	712.5256	0.0289
a^$_9$	836.5968	836.5648	0.0320	y_8	825.5808	825.5865	0.0057
b^$_9$	864.5917	864.5847	0.0070	x_8	851.5601	851.4989	0.0612

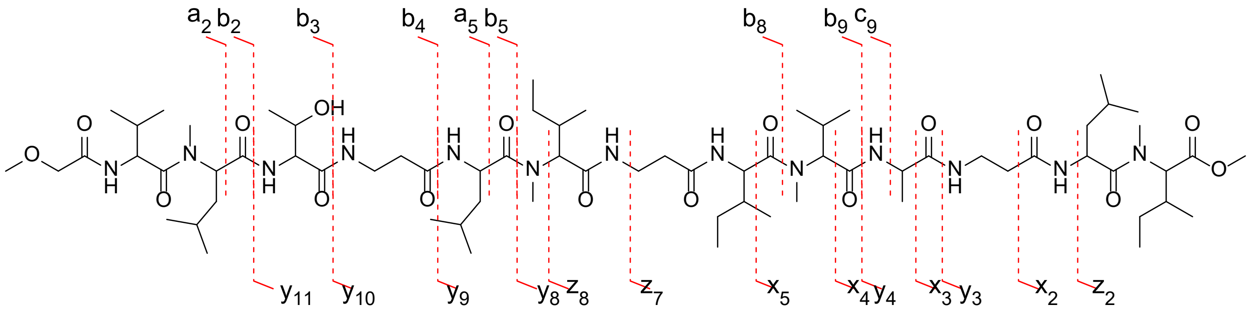

4a

Table S7 List of obtained fragment ions from MS/MS analysis of **4a**.

Ion	Calc. (Da)	Obs. (Da)	Δdelta (Da)	Ion	Calc. (Da)	Obs. (Da)	Δdelta (Da)
a_2	271.2016	271.1837	0.0180	z_2	256.1907	256.2704	0.0797
b_2	299.1965	299.1750	0.0215	x_2	299.1965	299.1750	0.0215
b_3	400.2442	400.2280	0.0162	y_3	344.2544	344.2978	0.0434
b_4	471.2813	471.2309	0.0505	x_3	370.2337	370.2358	0.0022
a_5	556.3705	556.3392	0.0312	z_4+H_2O	416.2650	416.3665	0.1016
b_5	584.3654	584.3403	0.0251	y_4	415.2915	415.3169	0.0254
b_8	895.5863	895.8677	0.2814	x_4	441.2708	441.3416	0.0709
b_9	1008.6704	1008.5639	0.1064	y_5+H_2O	546.3756	546.8662	0.4907
c_9	1025.6969	1025.7136	0.0167	x_5	554.3548	554.3998	0.0450
				x_6+H_2O	685.4389	685.5997	0.1608
				z_7	695.4702	695.7993	0.3291
				z_8	808.5543	808.5745	0.0203
				y_8	839.5965	839.5953	0.0012
				x_8+H_2O	883.5757	883.8066	0.2309
				y_9	952.6805	952.6453	0.0353
				z_{10}+H_2O	1024.6911	1024.6700	0.0210
				y_{10}	1023.7176	1023.6793	0.0384
				z_{11}+H_2O	1125.7388	1125.7618	0.0231
				y_{11}	1124.7653	1124.8483	0.0830

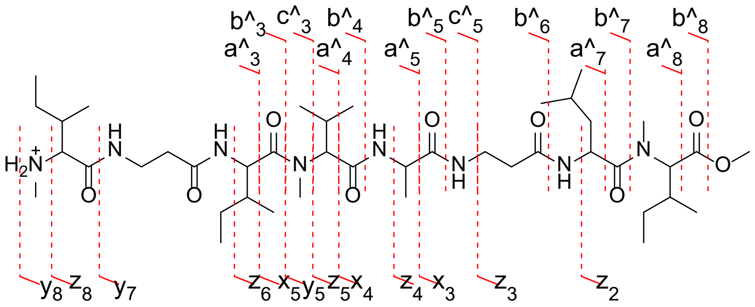

y_8-**4a**

Table S8. List of obtained fragment ions from MS/MS/MS analysis of y_8-**4a**.

Ion	Calc. (Da)	Obs. (Da)	Δdelta (Da)	Ion	Calc. (Da)	Obs. (Da)	Δdelta (Da)
$a^\wedge_3$	284.2333	284.2332	0.0000	z_2	256.1907	256.1338	0.0569
$b^\wedge_3$	312.2282	312.2136	0.0146	z_3	327.2278	327.2726	0.0448
$c^\wedge_3$	343.2704	343.1992	0.0712	x_3	370.2337	370.2361	0.0025
$a^\wedge_4$	397.3173	397.3780	0.0607	z_4	398.2650	398.2657	0.0007
$b^\wedge_4$	425.3122	425.3024	0.0098	x_4	441.2708	441.3000	0.0292
$a^\wedge_5$	468.3544	468.3275	0.0270	z_5	497.3334	497.3290	0.0044
$b^\wedge_5$	496.3494	496.3673	0.0179	y_5	528.3756	528.3574	0.0181
$c^\wedge_5$	513.3759	513.3659	0.0100	x_5	554.3548	554.4479	0.0930
$b^\wedge_6$	567.3865	567.3593	0.0272	z_6+H_2O	642.4331	642.5131	0.0800
$a^\wedge_7$	652.4756	652.4598	0.0158	z_6	624.4331	624.4572	0.0241
$b^\wedge_7$	680.4705	680.4728	0.0023	z_7+H_2O	713.4702	713.4330	0.0372
$a^\wedge_8$	779.5753	779.5262	0.0491	y_7	712.4967	712.4854	0.0114
$b^\wedge_8$	807.5702	807.5375	0.0328	z_8	808.5543	808.6328	0.0786

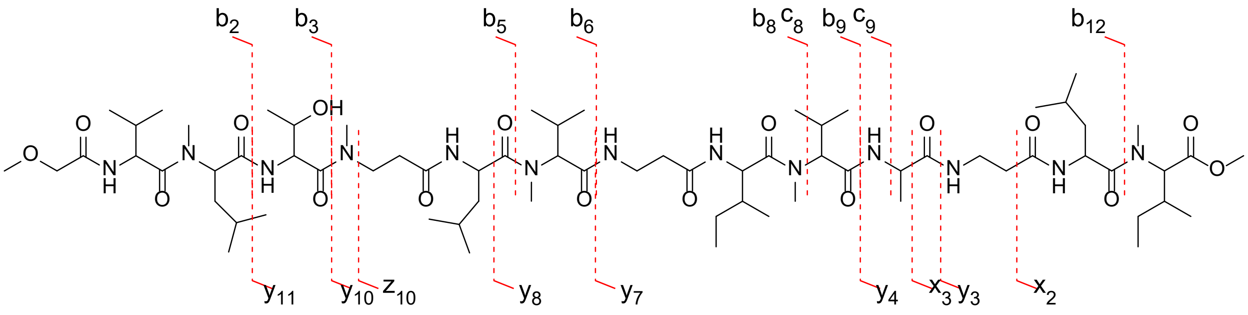

5a

Table S9. List of obtained fragment ions from MS/MS analysis of **5a**.

Ion	Calc. (Da)	Obs. (Da)	Δdelta (Da)	Ion	Calc. (Da)	Obs. (Da)	Δdelta (Da)
c_1+H_2O	221.1390	221.0938	0.0453	y_2+H_2O	291.2173	291.7729	0.5557
b_2	299.1965	299.1843	0.0123	x_2	299.1965	299.1843	0.0123
c_2+H_2O	334.2231	334.4922	0.2692	y_3	344.2544	344.2374	0.0170
b_3	400.2442	400.1840	0.0602	x_3	370.2337	370.3042	0.0705
b_4+H_2O	503.2970	503.3360	0.0390	y_4	415.2915	415.3901	0.0986
a_5+H_2O	588.3861	588.3979	0.0118	y_7	712.4967	712.4849	0.0118
b_5	598.3810	598.3754	0.0057	y_8	839.5965	839.5500	0.0465
b_6	725.4808	725.4555	0.0252	z_{10}	1006.6911	1006.7667	0.0756
c_6+H_2O	760.5073	760.4808	0.0265	y_{10}	1037.7333	1037.7349	0.0016
b_8	909.6019	909.5986	0.0034	z_{11}+H_2O	1139.7810	1139.8339	0.0529
c_8	940.6441	940.6146	0.0296	y_{11}	1138.7810	1138.7908	0.0098
b_9	1022.6860	1022.6556	0.0304				
c_9	1039.7125	1039.7324	0.0199				
b_{12}	1277.8443	1277.9799	0.1356				

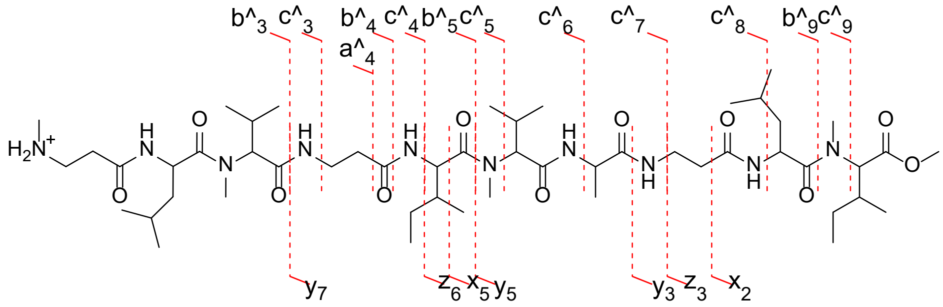

y$_{10}$-**5a**

Table S10. List of obtained fragment ions from MS/MS/MS analysis of y$_{10}$-**5a**.

Ion	Calc. (Da)	Obs. (Da)	Δdelta (Da)	Ion	Calc. (Da)	Obs. (Da)	Δdelta (Da)
$b^\wedge_3$	299.2562	299.1168	0.1394	x_2	299.1965	299.1168	0.0797
$c^\wedge_3$	327.2511	327.2304	0.0207	z_3	327.2278	327.2304	0.0025
$a^\wedge_4$	344.2776	344.2719	0.0057	y_3	344.2544	344.2719	0.0175
$b^\wedge_4$	369.2860	369.2168	0.0692	y_5	528.3756	528.3298	0.0457
$c^\wedge_4$	397.2809	397.2720	0.0089	x_5	554.3548	554.3840	0.0292
$b^\wedge_5$	482.3701	482.4616	0.0916	z_6	624.4331	624.4669	0.0338
$c^\wedge_5+H_2O$	528.3650	528.3298	0.0352	z_7+H_2O	713.4702	713.5400	0.0698
$c^\wedge_5$	510.3650	510.3632	0.0018	y_7	712.4967	712.4955	0.0012
$c^\wedge_6$	623.4491	623.4429	0.0062				
$c^\wedge_7+H_2O$	712.4862	712.4955	0.0094				
$c^\wedge_7$	694.4862	694.4308	0.0554				
$c^\wedge_8$	765.5233	765.5231	0.0002				
$b^\wedge_9$	850.6124	850.6707	0.0583				
$c^\wedge_9$	878.6074	878.5517	0.0557				

Table S11. Biological activity of the isolated compounds.

Comp.	MIC (μM)[a]			IC_{50} (μM) in DMEM[a]			PC_{50} (μM) in NDM[a]		
	EC[b]	BC[b]	KR[b]	HepG2	MIA PaCa-2	MCF 7	HepG2	MIA PaCa-2	MCF 7
**1**	>66	>66	>66	>100	>100	>100	>100	42.8	>100
**2**	>66	>66	>66	>100	>100	>100	>100	33.4	>100
**3**	>66	>66	>66	>100	73.2	>100	>100	10.0	>100
**4**	>66	>66	>66	>100	38.2	>100	>100	7.8	>100
**5**	>66	>66	>66	>100	40.4	>100	>100	3.5	>100
**6**	>66	>66	>66	>100	42.7	>100	>100	8.2	>100

[a]MIC (Minimum Inhibitory Concentration); PC (Preferential Cytotoxic); IC (Inhibitory Concentration).

[b]Tested bacteria (EC : *Escherichia coli* JW5503), and gram-positive (BC : *Bacillus cereus* NBRC 15305), and (BC : *Kocuria rhizophila* NBRC 12708).
